# Widespread Genome Reorganization of an Obligate Virus Mutualist

**DOI:** 10.1371/journal.pgen.1004660

**Published:** 2014-09-18

**Authors:** Gaelen R. Burke, Kimberly K. O. Walden, James B. Whitfield, Hugh M. Robertson, Michael R. Strand

**Affiliations:** 1 Department of Entomology, University of Georgia, Athens, Georgia, United States of America; 2 Department of Entomology, University of Illinois, Urbana-Champaign, Champaign, Illinois, United States of America; Fred Hutchinson Cancer Research Center, United States of America

## Abstract

The family Polydnaviridae is of interest because it provides the best example of viruses that have evolved a mutualistic association with their animal hosts. Polydnaviruses in the genus *Bracovirus* are strictly associated with parasitoid wasps in the family Braconidae, and evolved ∼100 million years ago from a nudivirus. Each wasp species relies on its associated bracovirus to parasitize hosts, while each bracovirus relies on its wasp for vertical transmission. Prior studies establish that bracovirus genomes consist of proviral segments and nudivirus-like replication genes, but how these components are organized in the genomes of wasps is unknown. Here, we sequenced the genome of the wasp *Microplitis demolitor* to characterize the proviral genome of *M. demolitor* bracovirus (MdBV). Unlike nudiviruses, bracoviruses produce virions that package multiple circular, double-stranded DNAs. DNA segments packaged into MdBV virions resided in eight dispersed loci in the *M. demolitor* genome. Each proviral segment was bounded by homologous motifs that guide processing to form mature viral DNAs. Rapid evolution of proviral segments obscured homology between other bracovirus-carrying wasps and MdBV. However, some domains flanking MdBV proviral loci were shared with other species. All MdBV genes previously identified to encode proteins required for replication were identified. Some of these genes resided in a multigene cluster but others, including subunits of the RNA polymerase that transcribes structural genes and integrases that process proviral segments, were widely dispersed in the *M. demolitor* genome. Overall, our results indicate that genome dispersal is a key feature in the evolution of bracoviruses into mutualists.

## Author Summary

Microorganisms form obligate associations with multicellular organisms that range from antagonistic (parasitic) to beneficial (mutualists). Although numerous examples of obligate, mutualistic bacteria, fungi, and protozoans exist, viruses are thought to usually form parasitic associations. An exception is the family Polydnaviridae, which consists of large DNA viruses that have evolved into mutualists of insects called parasitoid wasps. Each wasp species relies on its associated polydnavirus to parasitize hosts while each polydnavirus relies on its wasp for transmission. Polydnaviruses in the genus *Bracovirus* evolved approximately 100 million years ago from a group of viruses called nudiviruses, which are closely related to another large family of viruses called baculoviruses that are virulent pathogens of insects. Bracoviruses are of interest, therefore, because they provide a study system for examining how evolution into mutualists affects the structure and function of viral genomes. In this study, we sequenced the genome of the wasp *Microplitis demolitor* to characterize the proviral genome of *M. demolitor* bracovirus (MdBV). While the viral ancestor of bracoviruses possessed an independent circular genome, the proviral genome of MdBV is widely dispersed in the genome of *M. demolitor*. Our results also provide new insights into how the MdBV genome functions to produce virus particles that wasps rely upon to parasitize host insects.

## Introduction

Long-term associations between multicellular organisms and microbes are widespread. In the case of bacteria and fungi, several taxa contain species that have evolved into vertically transmitted, obligate mutualists or pathogens [1]–[3]. Traits inherited from ancestors and acquired by horizontal gene transfer have both contributed to the initiation and maintenance of these obligate associations [4]–[6]. In contrast, small effective population size associated with vertical transmission and increased levels of genetic drift appear to differentially affect genome size and architecture. Bacteria consistently exhibit size reductions due to mutational bias that causes deletions [7]–[9], while fungi trend toward genome expansion due to gains in mobile elements, intronic sequences, and other types of non-coding DNA [5], [10]–[13].

All viruses require another organism to persist and propagate with most species being horizontally transmitted by virions that are produced through replication. This lifestyle results in viruses often being pathogenic and having compact genomes with evolutionary rates that are usually much higher than the host organisms they infect [14]. Obligate associations occur when a viral genome integrates into the host germline to form a vertically transmitted endogenous viral element (EVE) [15], [16]. EVEs deriving from many types of viruses have been identified including some of ancient origin that have reached fixation in host populations and can no longer remobilize. Most EVEs are subject to their host's neutral rate of evolution, which results in persistence as fragments of the ancestral viral genome rendered non-functional by mutation [15], [17]. A few single genes or regulatory domains of viral origin have also been identified where natural selection has led to new, non-viral host functions [15], [18], [19]. The family Polydnaviridae is of interest because it is the only known example of viruses that have evolved into vertically transmitted agents that benefit their hosts yet do so by continuing to function in many respects like the viruses they evolved from [20]–[22]. As such, polydnaviruses (PDVs) have evolved into mutualists and provide a study system for understanding how their obligate associations with hosts affect viral genome architecture and function.

All PDVs are associated with insects called parasitoid wasps (Hymenoptera), which reproduce by laying eggs into other arthropods (hosts) their offspring consume [23]. The genus *Bracovirus* (BV) is associated with ∼50,000 wasp species in the family Braconidae: forming a monophyletic assemblage called the microgastroid complex that evolved ∼100 million years ago (Mya) [24]–[26]. Each wasp species in the complex carries a unique BV that persists in every cell of every individual as a provirus. However, BVs only replicate in calyx cells that are located at the base of the ovaries near the lateral oviducts [27]–[30]. BV virions package multiple circular, double-stranded (ds) DNAs of large aggregate size, which are released by lysis of calyx cells and stored in the oviducts [31], [32]. Females inject eggs containing the integrated provirus plus a quantity of virions into host insects they parasitize. The DNAs delivered by these virions integrate into the genome of infected host cells, while expression of virulence genes on these DNAs generates products that alter the physiology of hosts in ways that wasp offspring depend upon for survival [32]–[35]. BVs differ from other known EVEs of ancient origin because they retain the ability to replicate in wasps and produce infectious virions. However, BVs also differ from most viruses because replication in wasps produces virions that are incapable of replicating in the host insects wasps parasitize. The net result is that each BV relies on its associated wasp for vertical transmission as a provirus while each wasp relies on its BV to produce replication-defective virions for delivery of virulence genes needed to successfully parasitize hosts.

The monophyly of the microgastroid complex strongly suggests BVs evolved from an ancient virus that integrated into the germline of an ancestral braconid. Insights into the identity of this ancestor come from transcriptome studies of ovaries from three wasp species (*Cotesia congregata, Chelonus inanitus, Microplitis demolitor*), which identify more than 30 homologs of genes with predicted functions in replication from another group of insect-infecting DNA viruses called nudiviruses [27], [28], [36]. The family Nudiviridae is also the sister taxon to the family Baculoviridae that likewise infects insects. Most nudiviruses and baculoviruses are virulent pathogens that package a single large, circular dsDNA (>90 kb) genome into virions containing all genes required for infection of hosts and replication [37]–[39]. This suggests the nudivirus ancestor of BVs initially integrated into the germline of a wasp as a linear proviral DNA [20]. In contrast, BV genomes have since changed in a manner that has resulted in: 1) all of the nudivirus-like genes being integrated in the genomes of wasps and transcribed in calyx cells but none residing on the DNAs that are packaged into virions [27], [28], [36], 2) the DNAs packaged into virions encoding multiple virulence genes [40]–[44], and 3) almost none of these virulence genes being transcribed in wasps but most being transcribed in the hosts that wasps parasitize [33], [45], [46].

The circular, dsDNAs in BV virions are referred to as the encapsidated form of the genome [20], [33]. In turn, these DNAs are referred to as proviral segments when integrated in the genome of wasps, while the proviral segments and nudivirus-like genes together constitute the BV proviral genome [41], [47], [48]. Screening and sequencing of BAC genomic clones from four species in two genera (*Glyptapanteles indiensis, G. flavicoxis, Cotesia congregata, C. sesamiae*) have previously shown that BV proviral segments reside in multiple loci in the genomes of wasps [41], [47], [48]. Sequencing of BAC clones from *C. congregata* further show that 10 nudivirus-like genes reside in an 18 kb domain referred to as the nudivirus gene cluster [27]. These data combined with evidence that all BVs evolved from a common nudivirus ancestor have further led to the suggestion that proviral segment loci and the nudivirus cluster are physically linked in the genomes of wasps [22], [27], [47]. BAC clone sequence data, however, are too limited to provide direct evidence such linkages exist. In addition, many of the nudivirus-like genes identified in transcriptome studies [27], [28], [36] do not reside in the nudivirus cluster identified from *C. congregata*. As a result, the location of most nudivirus-like genes in the genomes of wasps, including several experimentally shown to be essential for replication [49], is also unknown.

Taken together then, prior studies clearly establish that BV proviral genomes consist of two components: proviral segments organized into loci and nudivirus-like genes [20], [22], [27], [28], [32], [41]–[43], [47]–[49]. In contrast, how these components are organized in relation to one another and where in the genomes of wasps most nudivirus-like genes identified from transcriptome studies reside is unknown for any species. Such information is important to issues ranging from understanding how BVs function to how genome content and architecture compares to nudiviruses and baculoviruses. The only means of addressing these questions though is through whole genome sequencing. In this study, we sequenced the microgastrine braconid *Microplitis demolitor*, which carries *M. demolitor* bracovirus (MdBV) and diverged from wasps in the genera *Cotesia* and *Glyptapanteles* ca. 53 Mya [24]. Assembly of the *M. demolitor* genome shows that MdBV proviral segments reside in multiple loci and that some nudivirus-like genes are clustered as found previously in *Glyptapanteles* and *Cotesia* wasps. However, we also determined that the MdBV nudivirus-like cluster is much larger than previously found in *C. congregata* and that a number of nudivirus-like genes that are functionally essential for replication are widely dispersed in the *M. demolitor* genome. Finally, our results provide direct evidence that MdBV proviral segment loci are not closely linked physically to one another or to the nudivirus-like cluster.

## Results

### Genome sequencing

We generated a draft genome sequence for *M. demolitor* using Illumina technology. The haploid genome size was estimated to be 241±6 Mbp by flow cytometry using wasp cell nuclei normalized to nuclei of *Drosophila virilis*. Based on this estimate, the *M. demolitor* genome was sequenced to 26× using haploid male genomic DNA from a lab culture maintained for more than 20 years with no introduction of additional field material. Sequencing of multiple small and large insert paired-end libraries (180 bp, 1.5 kb, 5 kb, and 10 kb) produced 1.04 billion raw reads. SOAPdenovo v2.04 (3) was employed with K = 49 to assemble the 180 bp-insert library reads into 357,737 contigs greater than 100 bp, totaling 195,839,919 bp with a contig N50 of 1,585 bp. Scaffolding with iteratively longer-insert mate-pair libraries followed by GapCloser v1.12 resulted in 5524 scaffolds consisting of two or more contigs in appropriate order and orientation separated by regions of approximately known lengths of unknown nucleotides. Remaining sequence data consisted of 47727 contigs greater than 100 bp. The scaffold N50 was 323,181 bp, the singleton contig N50 173 bp, and the total assembled genome sequence including intra-scaffold gaps was 258,751,082 bp. As discussed below, a total of 40 scaffolds with a cumulative size of 19.3 Mb contained elements of the MdBV proviral genome. Evidence supporting gene models (see Methods) identified 1,737 genes in the 40 scaffolds containing MdBV components of which 1,713 were predicted protein-coding sequences and 24 were tRNAs (Table S1). In addition, the estimated aggregate size of all components of the MdBV proviral genome accounted for less than 1% of the *M. demolitor* genome.

### MdBV proviral segment analysis

Our next goal was to identify regions of the *M. demolitor* genome that contained MdBV proviral segments. This was accomplished using a combination of previously published and newly generated data. As background, each MdBV virion produced during replication contains only one circular dsDNA segment [50]. Thus, the total complement of DNAs in the encapsidated genome of MdBV are not present in each virion but instead are distributed among the total population of virions produced during replication. In addition, the circular, dsDNAs in MdBV virions are non-equimolar in abundance, which results in delivery of a higher copy number of some DNAs to parasitized hosts than others [50]. Non-equimolar abundance of DNAs in virions from other wasp species [summarized by 20]–[23], [32], [33] combined with data showing that *Chelonus inanitus* BV also packages a single DNA per virion [51] suggests these features apply to BVs generally.

The encapsidated form of the MdBV genome was previously analyzed by isolating DNA from virions followed by construction of plasmid libraries that were Sanger sequenced. These data assembled into 15 circular dsDNAs (named A through O), which had an aggregate size of 190 kb [43]. However, this approach can lead to misassembly or omission of segments due to their non-equimolar abundance and the presence of repetitive DNA [40], [47]. Segments are also easily missed in the absence of having a reference proviral genome, which was a central goal of this study. Thus, we re-sequenced the circular, dsDNAs in MdBV virions by Illumina and then mapped these reads to the assembly of *M. demolitor* genome. This approach produced 50 million 100 bp read pairs. After quality filtering, 99% of 37 million read pairs were successfully mapped to *M. demolitor* genome scaffolds. The number of MdBV reads mapped to *M. demolitor* scaffolds containing proviral segments ranged from 281,000 to 28 million.

**Figure 1:**
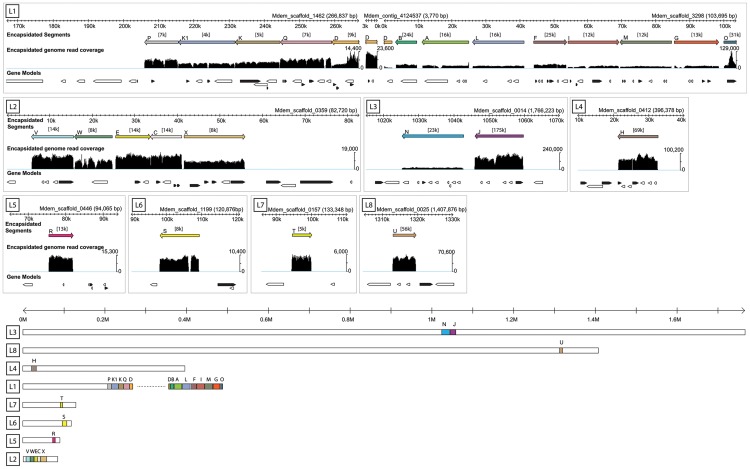
Genomic organization of MdBV proviral segment loci. The upper part of the figure presents the eight proviral loci identified (L1–L8) and the corresponding *M. demolitor* genome scaffolds where they are located. Only the portion of the scaffold where the proviral locus resides is shown. For each locus, the upper scale bar in kilobases (k) names the scaffold(s). Below the scale bar, colored bars indicate the segments identified by deep sequencing DNA from MdBV virions (Encapsidated segments) and their corresponding orientation and location in a given locus as a proviral segment in the *M. demolitor* genome. Average coverage in thousands of reads is indicated above each segment in brackets, while below each segment is shown read coverage per nucleotide relative to the scale indicated to the right of the graph. Gaps in read coverage indicate regions flanking individual proviral segments that are not amplified, excised and packaged into virions. The gap seen in segment S (L6) is due to a region of N's in the reference sequence. Below each MdBV proviral segment is shown predicted genes in forward (black) and reverse (white) orientation. Individual introns, exons and untranslated regions are not shown. The lower part of the figure shows each scaffold containing MdBV proviral loci in their entirety. Scaffolds are drawn to scale and organized from largest (L3) to smallest (L5). Scale bar is in megabases (M).

This data set recovered the 15 segments identified previously with the exception of segment O, which was only partially recovered (Figure 1). Segment O contains large repetitive regions [43], which likely prevented its successful assembly in this study. Read mapping identified the location of these DNAs as proviral segments in the *M. demolitor* genome (Figure 1). Segments A, E, G, I, and K were slightly larger than previously reported [43], while segment D was split between two scaffolds and one contig totaling 13,691 bp compared to the previously published size of 7,823 bp [43] (Figure 1). We also identified 10 previously unknown segments named K1, owing to sequence similarity with Segment K, and P through X. Thus a total of 25 proviral segments with an aggregate size of 278 kb are amplified and packaged into MdBV virions. In comparison, BVs from wasps in the genera *Cotesia* and *Glyptapanteles* have 30 to 35 proviral segments with aggregate sizes ranging from 517 to 731 kb [40], [41], [47].

#### Proviral segments are organized into eight loci dispersed in the *M. demolitor* genome

MdBV proviral segments resided in 8 loci on 11 scaffolds in the *M. demolitor* genome assembly. Figure 1 shows 10 of these scaffolds in the same orientation as reported for *Glyptapanteles* and *Cotesia* proviral segments [41], [47]. One scaffold not shown is Mdem_scaffold_3350, which contains genes from segment O but has very uneven read mapping coverage and no clear excision boundaries. Locus 1 contained 13 tandemly arrayed proviral segments, Locus 2 contained 5 segments, Locus 3 contained 2 segments and loci 4–8 contained one segment each (Figure 1). Domains ranging from 49–2861 bp separated tandemly arrayed segments in the same locus. Similar to *Glyptapaneles* and *Cotesia* proviral segment loci [41], [47], segments clustered in MdBV proviral Locus 1 were also noted to be primarily in the reverse orientation. Unlike prior studies though, assembly of the *M. demolitor* genome provided much larger scaffolds, which viewed in their entirety showed that most MdBV proviral loci are flanked by large amounts of wasp genomic DNA (Figure 1). This finding indicated that most if not all MdBV proviral segments are not closely linked physically in the *M. demolitor* genome (Figure 1).

#### Proviral segments share conserved wasp and host integration motifs

BV proviral segments are delineated by short, direct repeat junctions containing the tetramer AGCT that we previously named in MdBV as wasp integration motifs (WIMs) [35], [49]. Circularization of the DNAs packaged into virions results in segments that contain one WIM [35], [49], [52], [53], while functional studies indicate two nudivirus-like genes belonging to the tyrosine recombinase family (see below) exhibit recombinase activity required for these events to occur [49]. In contrast, most circularized segments MdBV virions deliver to parasitized hosts integrate into the genome of infected cells through a different conserved inverted repeat domain named the host integration motif (HIM) [35].

**Figure 2:**
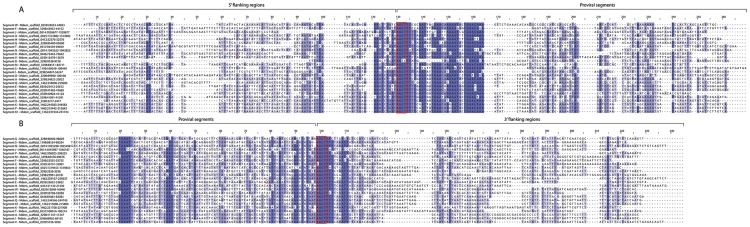
Wasp Integration Motifs (WIMs) for all MdBV proviral segments. (A) Alignment of 200 nucleotides (nt) surrounding the 5′ WIM site of each segment with similarity for each site colored in shades of blue. Red box highlights the tetramer AGCT. 31 nt are conserved in each proviral segment following the AGCT motif (red box), while the 5′ flanking region upstream of this motif is AT rich and not well conserved. (B) Alignment of 200 nt surrounding the 3′ WIM site for proviral segments shows that the flanking region preceding the WIM site is AT rich but not conserved, whereas the first 100 nt of each proviral segment downstream of the WIM site shows high conservation. Maximum likelihood analyses indicated that the relationships between segments for the WIM sites (A) and 3′ flanking regions (B) cannot be resolved.

**Figure 3:**
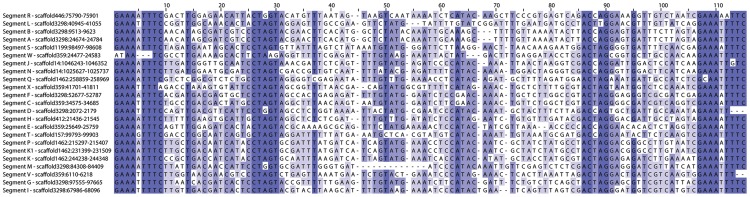
Relatedness of Host Integration Motif (HIM) sequences for all MdBV proviral segments. Alignment of HIM sequences with similarity for each site colored in shades of blue. Maximum likelihood analysis shows that three segment groups contain HIMs that form monophyletic groups (segments N and J; segments P, K1 and K; segments A and B) but other relationships could not be resolved.

In the current study, we confirmed that each MdBV proviral segment was flanked by WIMs (Figure 2). Read mapping sequence data from the circularized DNAs in MdBV virions to the *M. demolitor* genome identified boundaries that corresponded exactly to the WIMs flanking each proviral segment (Figure 1). We also validated that each new proviral segment was packaged into virions as a circularized DNA by PCR amplifying across the WIM in each segment using specific primers (Figure S1). We identified a HIM in each proviral segment except O and U, and also noted that several of these inverted repeats were imperfect palindromes (Figure 3).

The presence of flanking WIMs together with functional evidence that nudivirus-like tyrosine recombinases are required for processing and circularization of the DNAs packaged into virions suggest all MdBV proviral segments share a common ancestor which derives from the ancestral nudivirus [20], [22], [35], [49]. In contrast, sequence divergence limited insights into how different proviral segments were related to one another. Segments K and K1 reside next to one another in locus 1 (Figure 1), and contained regions of high identity indicative of a recent tandem duplication event (Figure S2). No other proviral segments, however, showed evidence of recent duplication although a few segments showed short regions of similarity suggestive of less recent duplication events (Figure S2). Sequence analysis of WIMs and HIMs also suggested five sets of segments were more related to each other than to other segments: 1) N and J, 2) P, K1, K, and Q, 3) A and B, 4) F and I, and 5) X, E and W (Figure 2, 3). Notably, the segments in each of these sets reside in close proximity to one another (Figure 1), which together with data presented in Figure S2 suggest segments near one another in the same locus tend to be more related than segments in different loci. We also noted that segments N, J, H and U (loci 3, 4 and 8) contain protein tyrosine phosphatase genes and are high abundance (see below), which also may suggest ancient shared ancestry [54], [55]. No other insights on how MdBV proviral segments were related to each other were identified. Overall, these data together with results from *Glyptapanteles* and *Cotesia* wasps [41], [47], [48], [54] suggest the greater similarity seen between adjoining proviral segments reflects tandem duplication events over evolutionary time followed by sequence divergence. It is also possible gene conversion could partly explain the greater similarity between WIMs and HIMs from adjoining proviral segments in the same locus versus segments on different loci.

#### Read mapping indicates proviral segments are differentially amplified

Mapping sequencing reads from the circularized DNAs in virions to the proviral segments in the *M. demolitor* genome allowed us to generate coverage calculations and also to estimate the relative abundance of each segment produced during replication (Figure 1). Coverage ranged from a low of 4,000× for segment K1 to 175,000× for segment J. These values correlated with abundance estimates determined previously by quantitative PCR for segments A-O [50] (Figure S3). We therefore concluded coverage estimates accurately estimate the relative abundance of the new segments we identified.

Segment abundance in BV virions reflects in large measure the degree to which a given proviral segment is amplified in calyx cells during replication [28], [50], [51]. Our abundance estimates suggested most proviral segments in locus 1 are similarly amplified (Figure 1). The exceptions to this trend were segments D and O. However, the repetitive nature of sequences within segments D and O clearly resulted in assembly problems and condensed read mapping, which reduced confidence in interpreting this result. In locus 2, proviral segments V, E, and C were more abundant than W and X, while in locus 3, segment J was dramatically more abundant than segment N suggesting these segments belong to different replication units (Figure 1). The remaining loci containing single segments also varied in abundance (Figure 1). Some of these new proviral segments were low abundance but others, such as segment U, were not (Figure 1).

#### Proviral segment gene content

Like other BVs [40]–[42], MdBV DNAs that are packaged into virions exhibit low gene coding densities and contain a mixture of single copy genes and genes that form multimember gene families [43]. A number of these genes also contain small introns but other genes on the DNAs in virions are intronless [43]. Previous sequencing of segments A-O identified 61 genes coding for predicted proteins ≥100 amino acids. These included 21 protein-coding single copy genes, four protein-coding gene families named the protein tyrosine phosphatase (PTP) family (13 genes), ankyrin-repeat (ANK) genes (12 genes), EGF genes (6 genes), and GLC genes (2 genes), and 7 tRNA genes for the cognate serine [43]. Some of these genes including members of the PTP, EGF, and GLC families contain introns of 60–120 bp while other genes are intronless. Searching for genes ≥83 amino acids, the current study identified all previously known genes plus several new protein coding genes. These genes are summarized in Table S1.

Figure S4 represents these data by showing which genes are present on each proviral segment, whether they contain introns or are intronless, and whether they are single copy or members of a gene family. Genes identified on the new segments (K1, P-X) included four new PTP family members located on segments U (2), N (1), and V (1) and two new viral ankyrin genes on Segment V. The number of BEN-domain containing genes also increased from 1 to form a 3-member family located on segments A, B and X. A total of 37 new hypothetical proteins related to genes present in other BV genomes were identified, while 8 hypotheticals unknown from other BVs were also identified (). No protein-coding genes ≥83 amino acids were identified on segments S and T although both possess flanking WIMs and are packaged into MdBV virions (Figure S4). However, it is possible these segments could encode genes that produce smaller proteins or peptides.

#### Domains of the *M. demolitor* genome flanking proviral segments share features with other BV-carrying wasps

As previously noted, BAC clone sequencing was previously used to characterize the proviral segment loci of two BVs (GfBV and GiBV) associated with *Glyptapanteles flavicoxis* and *G. indiensis*, and two BVs (CcBV and CsBV) associated with *Cotesia congregata* and *C. sesamiae* [41], [47]. The genera *Glyptapanteles* and *Cotesia* diverged from each other 17 Mya and from the genus *Microplitis* 53 Mya [24]. Global organization of proviral domains for these BVs and MdBV are broadly similar with each having two loci containing multiple segments in tandem array plus several smaller loci with 1–3 segments. However, most BV-carrying wasps are specialists that parasitize only 1 or a few host species [56]–[58]. This in turn is thought to result in arms race interactions and associated rapid evolution of the proviral segments packaged into virions and the genes on these segments [20], [22]. Thus, while some homologous segments are recognizable between BVs from closely related wasps, such as GfBV and GiBV, or CcBV and CsBV [41], [47], [54], but no segment homologies are recognizable between these BVs and MdBV.

**Figure 4:**
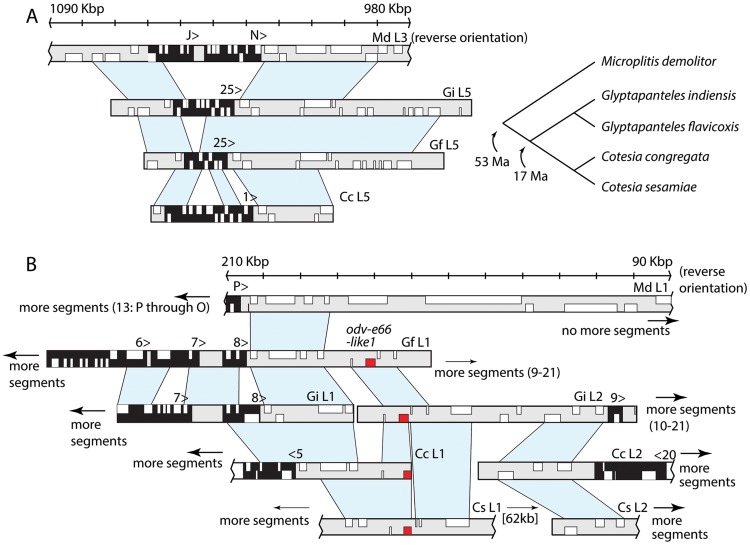
Two genomic regions flanking proviral segments are conserved among BV-carrying wasps. (A) The area surrounding segments N and J in *M. demolitor* Locus 3 is homologous to the regions surrounding segment 25 in Locus 5 of *G. flavicoxis* and *G. indiensis* and segment 1 in Locus 5 of *C. congregata.* *M. demolitor* loci are oriented to match the orientation of sequence scaffolds and similarity between flanking regions reported in prior studies. Although the genes in the proviral segments themselves are usually not recognizably homologous (black background with genes in white), the regions flanking these proviral segments (gray background with genes in white) are conserved as indicated by regions of synteny shaded in light blue. To the right of the figure is shown the phylogenetic relationships between these wasp species and estimated divergence times. (B) The downstream region flanking segment P in *M. demolitor* Locus 1 is similar to the region between Locus 1 and 2 in *G. flavicoxis, G. indiensis, C. congregata* and *C. sesamiae Kitale*. Genes and segments are depicted as in (A) except for *odv-e66-like1* homologs are indicated in red in the *Glyptapanteles* and *Cotesia* genomes. Although the *odv-e66* homologs are located in an area of synteny, no *odv-e66* homologs exist in this region for *M. demolitor*. Accession numbers for sequences are MdL3 (KK043340), GiL5 (EF710656), GfL5 (EF710650), CcL5 (HF586476), MdL1 (KK044729), GfL1 (EF710644), GiL1 (EF710657), GiL2 (AC191960), CcL2 (HF586473), CcL1 (HF586472), CsL2 (EF710635), CsL1 (EF710629).

Regions of the wasp genome flanking proviral segments in contrast should evolve more slowly because they are not involved in arms race interactions with host insects. This feature revealed conservation in flanking wasp DNA between *Glyptapanteles* and *Cotesia* BVs [47], while in this study we identified two regions of the *M. demolitor* genome flanking proviral loci with homology to *Glyptapanteles* and *Cotesia* species (Figure 4). The first of these flank segments N and J (locus 3) in *M. demolitor*, and were conserved with wasp DNA flanking segment 25 (locus 5) in *G. flavicoxis* and *G. indiensis*, and segment 1 (locus 5) in *C. congregata* (Figure 4A). The segments themselves shared little or no homology with one another but the most parsimonious explanation for conservation of the flanking regions suggests locus 3 in *M. demolitor* either gained a segment or loci 5 in *Glyptapanteles* and *Cotesia* lost one. The second example showed synteny between the upstream region flanking multi-segment locus 1 in *M. demolitor* and the domain between loci 1 and 2 in *Glyptapanteles* and *Cotesia* species, which also contain multiple tandemly arrayed segments (Figure 4B). Synteny extended ∼30 kb upstream from MdBV segment P followed by at least 170 kb of wasp DNA, which contained no additional proviral segments. Locus 1 in the *Glyptapanteles* and *Cotesia* genomes in contrast is followed by ∼100 kb of intervening wasp sequence and then locus 2 [41]. This intervening region in *Glyptapanteles* and *Cotesia* also contains one nudivirus-like gene (*odv-e66-like1*) (Figure 4B). No *odv-e66-like1* gene was detected upstream of locus 1 in *M. demolitor* although several other copies of this gene were identified elsewhere in the *M. demolitor* genome (see below).

### MdBV nudivirus-like genes

Comparative analyses of baculovirus genomes suggest all species share 37 core genes of which approximately half are required for replication [38], [59]. Nudiviruses, which diverged from baculoviruses ∼300 Mya [60], share 20 baculovirus core genes [38], [39], including a DNA polymerase predicted to replicate the viral genome, a DNA dependent RNA polymerase comprised of four subunits (*lef-4, lef-8, lef-9, p47*), and several structural genes with unique promoter features that are specifically recognized and transcribed by the viral RNA polymerase. Previous transcriptome sequencing by Illumina of *M. demolitor* ovaries during MdBV replication identified 41 genes with homology to nudivirus genes [28]. These include the four RNA polymerase subunits, several structural genes, and multiple tyrosine recombinases named *integrases (int)*, unknown from baculoviruses, but related to a baculovirus gene named *vlf-1*. A nudivirus/baculovirus-like *helicase* with putative roles in DNA replication was identified but a nudivirus/baculovirus-like DNA polymerase was not, which suggested that amplification of proviral segments during replication requires a wasp DNA polymerase [28]. The remaining nudivirus-like genes included 11 proteins unknown from baculoviruses [27], [28].

MdBV replication in calyx cells is extremely high with virion production exceeding replication levels for baculoviruses [28]. Experimental studies show the predicted MdBV RNA polymerase subunits form a functional holoenzyme that transcribes the nudivirus-like structural genes [49]. Nudivirus-like genes with predicted roles in capsid and envelope formation are also required for virion formation, while *vlf-1* and *int-1* are required for circularization of MdBV proviral segments [49]. Proteomic analysis further shows most predicted nudivirus-like structural proteins are present in MdBV virions [49], while studies with *C. congregata* and *C. inanitus* indicate homologs of these structural proteins are present in CcBV and CiBV virions [27], [36]. Thus, several lines of evidence strongly support that the BV RNA polymerase and several structural genes that are nudivirus/baculovirus homologs retain ancestral functions essential for replication and virion assembly.

To identify the nudivirus-like genes in the *M. demolitor* genome, we searched our assembly using BLASTN and TBLASTN with previously identified transcript and protein sequences as queries [28], [36]. This resulted in identification of all previously identified nudivirus-like genes plus a few unrecognized genes of potential nudivirus origin located on 29 scaffolds (Table S1). None of the new genes were homologs of a viral DNA polymerase. In addition, none of the nudivirus-like genes contained introns or were flanked by WIMs. Annotation indicated some of these genes resided in a multigene cluster, others were duplicated genes arrayed in tandem, and the balance were single genes separated by large stretches of intervening wasp DNA. The size and number of scaffolds together with large intervening regions of wasp genes collectively indicated MdBV nudivirus-like genes were widely dispersed in the *M. demolitor* genome.

#### Nudivirus-like gene cluster

**Figure 5:**
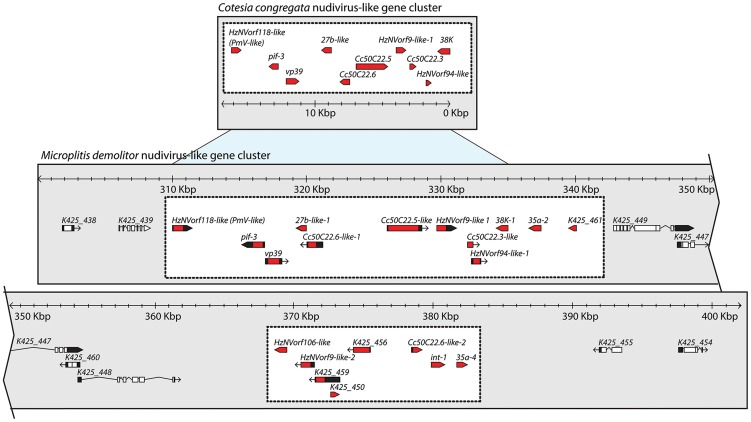
The nudivirus-like gene cluster in the genome of *M. demolitor* shares features with the partial nudivirus-like gene cluster from *C. congregata*. The *M. demolitor* nudivirus-like gene cluster is indicated in the lower portion of the figure by the two white boxes, while flanking domains containing predicted *M. demolitor* genes are indicated by the gray background. Regions of synteny between the *M. demolitor* nudivirus-like gene cluster and *C. congregata* nudivirus-like cluster are indicated by light blue. Black regions in gene models represent UTRs, black lines indicate introns, while red and white regions represent exons for nudivirus-like genes and wasp genes respectively. Scale bar indicates the size of these clusters in kilobase pairs (Kbp). Accession numbers are FM212911 for the *C. congregata* nudivirus-like cluster, and KK043480 for the *M. demolitor* cluster.

As noted above, 10 nudivirus-like genes were previously identified in an 18 kb domain of the *C. congregata* genome [27]. Assembly of the *M. demolitor* genome identified a ∼75 kb domain containing orthologs of these genes in the same order and orientation as the *C. congregata* cluster plus 10 additional nudivirus-like genes (Figure 5). Predicted wasp genes containing large introns demarcated the outer boundary of the *M. demolitor* nudivirus-like gene cluster. The *M. demolitor* nudivirus-like gene cluster was also interrupted by four intron-containing genes, which may represent an insertion or boundary that divides the *M. demolitor* nudivirus-like gene cluster in half. Many of the nudivirus-like genes in the *M. demolitor* cluster code for structural proteins previously identified in MdBV virions [49]. These include VP39, 38K, and PIF-3. Four genes in the *M. demolitor* cluster (K425_459, K425_456, K425_450, K425_461) lacked introns, which suggest they may have derived from the nudivirus ancestor of BVs but none were previously recognized as such because no homologs of these genes exist in other known nudiviruses or baculoviruses [28].

#### Duplicated genes arrayed in tandem

Three nudivirus-like genes in the *M. demolitor* genome were duplicated one or more times to form tandem arrays along scaffolds (Table S1). The most extreme was *odv-e66*, which is present in many places in the *M. demolitor* genome, and has also duplicated in arrays on Mdem_scaffold_0004 (2 copies), Mdem_scaffold_1559 (4 copies), Mdem_scaffold_0844 (3 copies), and Mdem_scaffold_0919 (5 copies). The gene *35a* was also present in different locations and duplicated on Mdem_scaffold_0004 (2 copies) and Mdem_scaffold_0919 (7 copies). Finally, *pif-5* was duplicated once on Mdem_scaffold_0040.

#### Loosely associated and single genes

**Figure 6:**
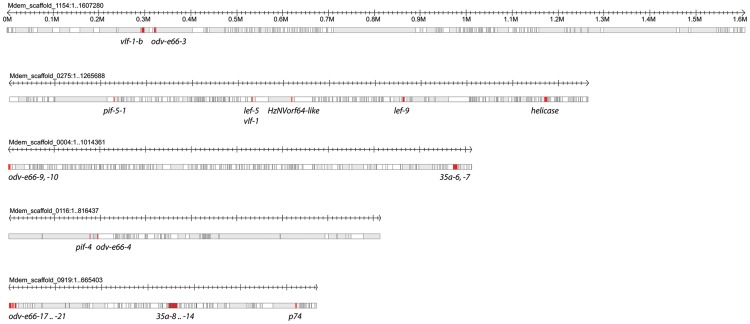
Distribution of nudivirus-like genes in loosely associated clusters in the *M. demolitor* genome. Five scaffolds containing nudivirus-like genes arranged from largest (1.6 Mb) to smallest (660 Kb) are shown in in gray. Named nudivirus-like genes (red) are separated by large stretches of DNA containing predicted *M. demolitor* genes (white). Wasp genes are depicted without indicating introns, exons, and untranslated regions.

Five scaffolds ranging from 0.7–1.6 Mbp contained 2 to 13 nudivirus-like genes but each of these genes was separated from the other by multiple wasp genes (Figure 6). Among these was Mdem_scaffold_0275, which contained *vlf-1*, the RNA polymerase subunit *lef-9*, and *helicase*. The remaining nudivirus-like genes including the three other RNA polymerase subunits (*lef-4, lef-8, p47*) and *int-1* were located as single genes on 18 scaffolds ranging from 1 kbp to 1.95 Mbp in size (Table S1).

#### Updating the MdBV conserved gene set

Compared to previous transcriptome sequencing of *M. demolitor* ovaries [28], assembly of the *M. demolitor* genome allowed identification of one more homolog of *odv-e56* and *HzNVorf128*, 16 more variants of *odv-e66* (up from 5 full-length copies to a total of 21), and 11 more variants of *35a*. The hypotheticals *k425_459, k425_456, k425_450*, and *k425_461* in the multigene cluster were all present in our previously generated ovary transcriptome data set as genes upregulated during MdBV replication [28]. Another nudivirus-like gene of unknown function was identified on a scaffold carrying Segment T, which is similar to *HzNVorf93* and a homolog from the shrimp *Penaeus monodon* nudivirus. Although unrecognized, this gene was upregulated during MdBV replication as evidenced by its presence in our previously published ovary transcriptome data set [28]. Finally, three pseudogenes of *vlf-1-b1, vlf-1* and *pif-1* were identified that are truncated and have very short regions of homology with the active full-length forms of these genes.

#### Nudivirus-like gene promoters

Baculovirus genes with functions in replication are sequentially transcribed and thus referred to as early, late and very late genes [38]. This is also true for MdBV and other BVs where the nudivirus-like genes can be divided into early and late categories [27], [28]. Early genes include the RNA polymerase subunits (*p47, lef-4, lef-8, lef-9*) and the initiation factor *lef-5*, which based on the baculovirus literature are thought to be transcribed by wasp RNA polymerase II [49]. Most other nudivirus-like genes are late genes that code for virion structural components. The RNA polymerase holoenzyme of baculoviruses transcribes late and very late genes by specific recognition of unique promoter sequences [38], [61]. This finding also suggests MdBV late genes possess baculovirus-like late gene promoter elements given evidence late genes are specifically transcribed by the MdBV RNA polymerase holoenzyme [49]. However, our analysis of upstream sequence for MdBV nudivirus-like genes yielded complex patterns. Three of 6 early MdBV genes lacked signatures of early promoters recognized by RNA polymerase II (TATAA box, followed by CA[G/T]T), but 4 of 6 had baculovirus-like late promoter sequences ([A/T/G]TAAG) [38], [61], [62]. In addition, only 64% of MdBV late genes had baculovirus-like late promoter motifs upstream of coding sequences. Among the late genes lacking such motifs were *vp39*, which is the most abundant component of capsids, and *p74*, which is an envelope protein [49].

### Linkages between proviral segments and nudivirus-like genes

While proviral segment loci and nudivirus-like genes have been suggested to be physically linked in the genomes of wasps [21], [22], [27], our assembly of the *M. demolitor* genome indicated most nudivirus-like genes, including those in the nudivirus-like gene cluster, reside in locations distant from proviral segments. The only direct physical linkage we identified was in Mdem_scaffold_0157, which contained the nudivirus-like gene *HzNVorf93* and 5.4 kb away MdBV proviral segment T (Table S2).

We identified 18 wasp gene families shared between scaffolds containing nudivirus-like genes and proviral segments (Table S2). These included members of a gene family with an EB module present in five scaffolds containing nudivirus-like genes and also the scaffold containing proviral segment U. Several members of a protein tyrosine kinase family were also present on a scaffold (Mdem_scaffold_0407) containing *lef-4*, and the scaffolds containing proviral locus 3 (Mdem_scaffold_0014) and proviral locus 8 (Mdem_scaffold_0025). A family of histone genes was shared among the scaffolds containing proviral loci 1, 3, 5 and 7, which suggested these genes may link the genomic neighborhoods of these proviral segments (Table S2). Members of 13 other wasp gene families were detected on scaffolds containing nudivirus-like genes. Among these was a family of MFS sugar transporter domain-containing proteins present on two scaffolds (Mdem_scaffold_0004, Mdem_scaffold_0938) containing nudivirus-like genes (*odv-e66*, *lef-4*). This finding is of potential interest because two MFS sugar transporter genes reside on homologous proviral segments of GiBV and GfBV, which provides an example of duplication and transfer of wasp genes into BV proviral segments [41].

## Discussion

This study advances our understanding of BVs by providing the first overall picture of proviral genome organization. Unlike the compact, circularized genomes of nudiviruses and baculoviruses, our results show that proviral segment loci and nudivirus-like genes are highly dispersed in the *M. demolitor* genome. Our results also show that none of the MdBV proviral segment loci are physically closely linked to the nudivirus-like gene cluster. Parasitoid wasps are among the most species-rich animal groups on Earth with estimates suggesting more than 1,000,000 species worldwide [23], [25] yet only one parasitoid wasp genome (*Nasonia vitripennis*) has been sequenced, assembled and annotated [63]. *N. vitripennis* belongs to a taxon of Hymenoptera that is distantly related to microgastroid braconids and has no association with polydnaviruses. Thus more broadly our results provide a genome for a second parasitoid wasp and the first species that is a polydnavirus carrier.

Illumina sequencing the DNAs in MdBV virions and mapping these reads back to the *M. demolitor* genome identified several DNA segments an earlier study failed to detect [43]. The 25 proviral segments now identified in 8 loci likely represent most if not all of the DNAs packaged into MdBV virions. We also identified all nudivirus-like genes found previously by transcriptome analysis of *M. demolitor* ovaries plus several unrecognized variants of these genes. In our view, the most important new findings from these data are: a) the nudivirus-like gene cluster of MdBV contains many more genes and overall is much larger than recognized from earlier data generated from *C. congregata* [27], b) most of these genes are structural components of BV virions, and c) the four nudivirus-like RNA polymerase subunits previously shown to regulate expression of BV structural genes reside outside of the nudivirus-like gene cluster as single genes that are widely dispersed in the *M. demolitor* genome.

Like other large DNA viruses, baculoviruses and nudiviruses exhibit high diversity in gene content outside of their core gene sets [37], [38]. It is also well known that different lineages of baculoviruses and nudiviruses have acquired many genes from their arthropod hosts and other organisms by horizontal gene transfer and other mechanisms. Given this, it is fully possible some genes from the nudivirus ancestor of BVs remain unidentified given our reliance on sequence similarity for recognition of genes of nudivirus origin [37], [38]. Primary structure together with proximity to conserved nudivirus-like genes identified four hypotheticals in the MdBV nudivirus-like gene cluster that potentially derive from the nudivirus ancestor. In contrast, identifying genes outside the nudivirus-like gene cluster derived from the nudivirus ancestor will be very difficult in the absence of data linking a given product to replication or other virus-related activities.

The dispersed architecture of the MdBV proviral genome is remarkable in light of the very high levels of replication that occur in calyx cells following pupation of female wasps. Although viral genomes are typically viewed as a contiguous stretch of DNA or RNA, our results clearly show that dispersal of BV genomes does not functionally impede high-level amplification of a portion of the proviral genome or production of virions. We suggest the physical separation of nudivirus-like genes required for virion formation from the proviral segments containing virulence genes is selectively advantageous for wasps because it assures vertical transmission of the entire proviral genome but prevents any replication machinery from escaping, which could be deleterious to wasp offspring developing in a host. Our results also suggest two *trans*-acting factors play critical roles in linking the physically separated components of the MdBV proviral genome together. First, the two nudivirus-like integrases (*int-1, vlf-1*), once transcribed and translated in calyx cells, likely use WIMs to recognize all proviral segments for processing regardless of their location in the wasp genome. Second, the MdBV RNA polymerase holoenzyme, once made, specifically transcribes the nudivirus-like structural genes through promoter recognition, which is similar to baculovirus RNA polymerases that also specifically transcribe structural and other late viral genes [49]. Our analysis of upstream sequence indicates some MdBV structural genes have baculovirus-like late gene promoter motifs but others do not, which suggests the promoter sequences BV RNA polymerases recognize differ somewhat from those of their nudivirus/baculovirus ancestors. Identification of these recognition sequences is not amenable to computational analysis and will require experimental studies.

Based on the baculovirus literature [38], we hypothesize wasp RNA polymerase II transcribes the MdBV integrase and RNA polymerase genes, but the factors responsible for restricting transcription to only calyx cells are unknown. The absence of any baculovirus/nudivirus-like DNA polymerase in the *M. demolitor* genome further strengthens earlier conclusions that a wasp DNA polymerase(s) amplifies MdBV proviral DNAs prior to their excision, circularization, and packaging [28], [49]. However, the specific polymerase responsible also remains unidentified. In contrast, it has long been known the DNA segments in BV virions are non-equimolar in abundance [40]–[42], [50], which our read mapping data indicate is due to proviral segments in different loci being differentially amplified. Similar levels of MdBV proviral segment amplification in loci 1 and 2 are also broadly similar with recent findings for CcBV where multiple adjoining proviral segments are co-amplified before processing into circularized DNAs [64].

Although most nudiviruses and baculoviruses establish systemic lytic infections that are fatal to hosts, one nudivirus has been identified that in vitro establishes long-term persistent infections associated with integration into the host genome [65], [66]. Such latent infections can also be reactivated. This suggests the nudivirus ancestor of BVs may have established a latent infection following integration of one or more copies of its genome into the germline of the braconid ancestor of microgastroids. This integration event was then followed by a series of modifications and rearrangements to arrive at the current dispersed architecture shown here for MdBV in *M. demolitor*. Reconstruction of these events for BVs generally is theoretically possible through comparative data of wasp species in the microgastroid complex with different divergence times. However, with near complete data on proviral genome architecture limited to *M. demolitor* and partial data available for just 4 other species [41], [47], only a few suggestive patterns are currently possible.

First, experimental studies in *M. demolitor* combined with conservation of these nudivirus-like genes in two other microgastroid wasps (*C. congregata, C. inanitus*) [27], [36], [49] strongly suggest natural selection has maintained the ancestral functions of these factors in virion formation despite their dispersal in the genomes of wasps. Second, the conserved synteny of predominantly structural genes in the nudivirus-like cluster of *M. demolitor* and *C. congregata* suggests this domain represents an initial integration site for the nudivirus ancestor, and that maintenance of these genes in a cluster is functionally important for virion formation. These data also indicate the MdBV and CcBV nudivirus-like clusters have remained stable since divergence 53 Mya, which suggests dispersal of the other nudivirus-like genes occurred relatively early in BV evolution. Comparative sequence data from additional BV-carrying wasps will reveal whether dispersal is highly variable or dispersed genes reside in similar locations in the genomes of different wasps. Third, the distribution of MdBV proviral segment loci indicates these domains are also not clustered in the *M. demolitor* genome, while the presence of only one nudivirus-like gene near a proviral locus indicates that proviral loci and nudivirus-like replication genes reside distantly with respect to one another. We do identify a few wasp gene families shared between scaffolds containing nudivirus-like genes and/or proviral segments but without additional comparative data it remains unclear whether these genes are indicative of physical linkages that are conserved among microgastroid wasps generally. Future assemblies of the *M. demolitor* genome will eventually identify linkages between at least some of the MdBV proviral segment loci and nudivirus-like genes. However, based on the assembly used for this study, we conclude these linkages will not be in near proximity to each other.

The finding that all BV proviral segments are flanked by WIM sequences together with evidence that nudivirus-like tyrosine recombinases recognize these motifs to produce circularized segments suggest both of these elements derive from the ancestral nudivirus genome [35], [41], [49], [52], [53], [64]. Despite rapid evolution obscuring proviral segment homology [20]–[22], the similarities in architecture of proviral segment loci between MdBV and GfBV, GiBV and CcBV also suggest shared ancestry. No data currently exist regarding motifs associated with integration of nudiviruses into the genomes of insects. On the other hand, if integration motifs with homology to WIMs or HIMs were identified from nudiviruses, it could provide important insights into the relationship between BV proviral segments and the nudivirus-like genes required for virion formation, proviral segment excision from the wasp genome, or segment integration into the genome of parasitized host insects.

Duplication of genes into families is a recurring theme that serves as a key source of novelty in the molecular arms races that occur between parasites and hosts [51], [67]. For MdBV and other BVs, previous studies show the virulence genes on proviral segments have diverse origins. Previously conducted evolutionary analyses show that some gene families on proviral segments, such as the sugar transporter genes identified from BVs associated with *Glyptapanteles* wasps and EGF gene family in MdBV are relatively recent acquisitions from wasps [41], [58]. In contrast, other families show evidence of acquisition from other organisms or in the case of the PTP and Ank genes are of uncertain ancestry including possibly deriving from the nudivirus ancestor [54], [55], [68]. Prior studies also indicate that BV virulence gene family diversification has occurred through duplications plus rearrangements within and between segments, while also showing that some gene family members exhibit signatures of positive selection in response to arms race interactions with hosts [20]–[22], [54], [55], [68]–[70]. In contrast, it currently is not possible to analyse how BV proviral segments have evolved among microgastroid braconids as a group because data are available for only five species in three genera including *M. demolitor*. Addressing this issue will require data from far more taxa.

For *M. demolitor*, venom glands and teratocytes secrete large amounts of proteins that females introduce together with MdBV to parasitize hosts [58]. These products exhibit almost no overlap with MdBV genes, but notably many derive from gene families that have also diversified by duplication for selective expression in venom glands or teratocytes. In the current study, we find that some nudivirus-like replication genes have also duplicated more extensively than previously recognized with *odv-e66* and *35a* in particular being potentially significant in parasitism of hosts because products from both families are present in virions [49].

Variation in the size and contents of bacterial and eukaryotic genomes is thought to result from differences in effective population size and degree of genetic drift [7], [11]. Genome evolution for vertically transmitted entities like BVs is largely unexplored, but is subject to different evolutionary processes compared to bacterial and eukaryotic symbionts, which retain their own cellular architecture and whose genomes are physically separated from that of their host. What we can conclude is that first, persistence as EVEs results in BV proviral genomes being inherited like other alleles in the wasp genome, which in turn subjects each BV to the effective population size of its associated wasp species. Second, on a broad scale BV proviral genomes show clear evidence of expansion relative to baculoviruses and nudiviruses. This is especially the case for proviral segments where decreases in gene density, increases in intron frequency, and gene acquisition from different sources followed by duplication are clearly apparent [40]–[44]. Interestingly, the direct repeat boundary regions recognized by viral integrases and tRNA loci, often associated with integration events, are features of proviral segments that are shared with pathogenicity islands in the genomes of disease-causing prokaryotes [71]. Pathogenicity islands initially evolve by horizontal gene transfer followed by site-specific recombination. Such processes could in part underlie the evolution of BV proviral segments as wasps adapt to parasitism of particular host species or guilds of closely related host species, and hosts reciprocally evolve to resist parasitism. Other studies have noted similarities between duplication of BV virulence genes and amplification of genes in insects associated with resistance to insecticides [47].

Many nudivirus-like genes in contrast show extreme dispersal throughout the *M. demolitor* genome but only a subset of these genes, also with potential roles in parasitism of hosts by wasps, have duplicated. Dispersal itself could occur through random processes of genome flux in wasps, similar to the rapid loss of microsynteny found by comparison of 12 species genomes in the genus *Drosophila*. Little is known currently about rates of genome flux in hymenopterans, although sufficient comparative genome data should eventually become available for BV-carrying species to perform a microsynteny analysis to see if proviral segments and nudivirus-like genes are similarly or more dispersed in the genome than wasp genes.

Besides BVs, the Polydnaviridae currently contains a second genus, the *Ichnovirus* (IVs), associated with parasitoid wasps in the family Ichneumonidae [20], [21]. Recent evidence strongly suggests that IVs evolved independently of BVs from a still unknown virus ancestor(s) [72]. Nonetheless, while IV proviral segments encode largely different virulence genes, they exhibit many of the same organizational features as BV proviral segments, which suggests convergent evolution driven by the similar roles BVs and IVs play in parasitism [20], [21]. Thus, the Polydnaviridae was originally recognized as a family because of the similarities in how the encapsidated form of BV and IV genomes are organized and their similar functions in parasitism of hosts by their associated wasps [20]–[22], [33]. However, current data also now indicate this is a non-natural taxon that will be revised in the future.

Most EVEs in animal genomes are non-functional fragments but a few cases are known of single viral genes or regulatory elements that have been exapted by hosts for new beneficial functions. Among these are mammalian syncytins derived from endogenized retrovirus *env* genes which function in placental development, and the *Fv4* and *Fv1* genes, also of retroviral origin, which function in antiviral defense [reviewed in 15], [73]. Such EVEs are appropriately viewed as no longer being viruses because they no longer function as such [15], [16], [18]. BVs have also evolved to benefit wasps yet differ from the previous examples because their beneficial roles in parasitism depend on many genes whose functions remain the same as those of their ancestor [49]. BVs also retain much of the ancestral replication machinery and produce infectious particles that are quite similar to nudiviruses and baculoviruses [20]. BVs are thus ancient EVEs that benefit wasps but do so by continuing to function in many respects like a virus. As such, BVs also share features with other microbes that are viewed as obligate mutualists in which neither the symbiont nor host can survive without the other.

BV genes required for replication are clearly of nudivirus origin. Genes on proviral segments on the other hand have a mixture of origins that include acquisition by horizontal transfer from wasps or other eukaryotes plus genes and motifs like WIMs that are ancient and exhibit features at least suggestive they too originated from the nudivirus ancestor [20]–[22]. Nudivirus and baculovirus genomes likewise consist of genes that are ‘viral’ in the sense they produce products required for replication, yet also contain genes and motifs of ancient origin and uncertain ancestry, plus genes acquired from arthropod hosts or other eukaryotes that function as virulence factors [38]. So what differs between BVs and their ancestors is not so much the types of genes they encode and their origins, but rather how their genomes are organized. Nudivirus and baculovirus genes, like those of most viruses, reside on a contiguous stretch of nucleic acid that is packaged into virions, whereas BV genes are organized in a manner that prevents all of them from being packaged into virions, which in turn also prevents BVs from existing independently of wasps. Other non-viral microbes that have evolved into vertically transmitted obligate mutualists do not persist by integrating into the genome of their host, but they too often exhibit profound alterations in genome organization and function that result in them no longer being genetically independent entities. The bacterium *Buchnera* in aphids, for example, was acquired in a single event that occurred more than 100 Mya, yet strict co-speciation thereafter has resulted in the phylogenies of these symbionts and aphids mirroring one another [74]. Thus, similar to discussions regarding whether entities like *Buchnera* are organelles or bacteria [9], [75], BVs will be viewed by some as EVEs that have been exapted by wasps to produce a novel organelle and others, including ourselves, as viruses that have evolved into wasp mutualists. Future studies will undoubtedly further advance our understanding of these fascinating associations.

## Materials and Methods

### Ethics statement

All studies were approved by the Biological Safety and Animal Care and Use Committee of the University of Georgia and were performed in compliance with relevant institutional policies, National Institutes of Health regulations, Association for the Accreditation of Laboratory Animal care guidelines, and local, state, and federal laws.

### DNA isolation, library preparation and sequencing

*M. demolitor* genomic DNA was isolated from single and pooled male wasps stored at −80 C. Briefly, 50 frozen males were pooled and ground in liquid nitrogen with mortar and pestle before lysing in a SDS solution overnight with Proteinase K. The homogenate was treated with RNaseA, and proteins/debris were collected after high-salt precipitation and centrifugation. After ethanol precipitation, the DNA was resuspended in 10 mM Tris and evaluated on an agarose gel and by Qubit quantification.

The W.M. Keck Center for Comparative and Functional Genomics at the University of Illinois at Urbana-Champaign generated the following libraries for sequencing: a 180 bp-insert library from a single wasp, and 1.5 kb, 5 kb, and 10 kb-insert mate-pair libraries from pooled wasp DNA. The 180 bp and 1.5 kb-insert shotgun libraries were prepared with Illumina's TruSeq DNAseq Sample Prep Kit. The 5 kb and 10 kb mate-pair libraries were prepared similarly except a custom linker was ligated between the read-ends to facilitate mate-pair recovery. All libraries were sequenced for 100 cycles on a HiSeq2000 using the TruSeq SBS Sequencing Kit v.3. Data were analyzed with pipeline versions 1.8 and 1.9. An additional 5 kb mate-pair library was constructed and sequenced by the Beijing Genome Institute using pooled wasp genomic DNA with all reads trimmed to 36 bp before assembly.

The custom 5 kb and 10 kb mate-pair libraries were filtered for reads containing properly-oriented reads of the appropriate insert size and uniqueness using in-house custom pipeline scripts. Raw Illumina reads were 5′- and 3′-trimmed for nucleotide-bias and low-quality bases using the FASTX Toolkit (http://hannonlab.cshl.edu/fastx_tookit/). Trimmed reads were error-corrected by library with Quake [76] counting 19-mers. SOAPdenovo v2.04 [76] was employed with K = 49 to assemble the 180 bp-insert library reads followed by scaffolding with iteratively longer-insert mate-pair libraries and use of GapCloser v1.12 to close gaps generated in the scaffolding process with short paired read data [77].

MdBV DNA was isolated from virions as described previously [28]. The DNA pellet was resuspended in 10 µl of H_2_O and 1 ul was used as template in four phi29 amplification reactions as performed previously [78]. To resolve amplified DNA, the amplified product was incubated with S1 nuclease (NEB) at 37°C for 20 min. Precipitation and re-suspension in H_2_O yielded a total of 5 µg for Illumina sequencing. The sequencing library was prepared by the University of Georgia Genomics Facility using the Illumina TruSeq DNA sample preparation kit and the standard low-throughput protocol, and sequenced with the Illumina HiSeq system housed at the HudsonAlpha Institute for Biotechnology (Huntsville, AL).

### Scaffold selection for annotation

Illumina sequenced reads from MdBV virions were filtered to retain read pairs with PHRED score equivalents >30 for >90% of nucleotides. Paired reads were mapped to the *M. demolitor* scaffolds using the bwa sampe algorithm and samtools [79], [80]. Tablet was used to view the mapped reads relative to the reference genome, and scaffolds with clear read coverage boundaries indicating the presence of segments were selected [81]. These scaffolds had >280,000 mapped reads and were all >1 kb in size. The remaining scaffolds were identified by BLAST, indicating the presence of nudivirus-like genes. The longest translated ORFs from transcripts previously identified as nudivirus-like genes expressed in *M. demolitor* ovaries [28] were queried against the whole genome scaffolds and contigs BLAST database. BLAST results were manually filtered to retain real hits, which were filtered to have minimum 30% identity and at least 200 amino acids in alignment length with queries. These scaffolds were selected for annotation along with those identified by MdBV read mapping.

### RNA isolation, library preparation, and transcriptome sequencing

Wasp transcriptome reads were generated and assembled previously from ovaries, teratocytes, venom glands, and wasp larvae [58], and also from *Pseudoplusia includens* cells infected with MdBV. The wasp derived reads were assembled *de novo* using Trinity with the jaccard clip option, resulting in a total of 216,988 transcripts from 173,925 loci [82]. Reads were re-mapped to the assembled transcripts using bwa bwasw, successfully mapping 89–97% of reads for each tissue type [80]. The overall reads per kilobase per million reads mapped (RPKM) values were used to filter out low abundance transcripts (<5 RPKM), in addition to the length of transcripts (<500 bp removed) [83]. The resulting filtered transcripts were used as evidence in gene model predictions described below. Forty eight million *P. includens* read pairs were generated and also used as described below.

### PCR verification of segment circularization

DNA was prepared from virus extracted from ovaries as above with DNAse treatment or with whole ovaries or animals without DNAse treatment. PCR was performed in a 10 µl reaction containing segment specific primers specific for amplifying across the WIM domain in a circularized segment (2.5 pmol) (Table S3), 0.25 units of Hotmaster *Taq* polymerase (5 Prime) and 1 µl of DNA as template. Reactions were run in a Bio-Rad thermocycler for 35 cycles with the following cycling conditions: initial denaturation at 94° for 2 min, followed by 35 cycles of denaturation at 94°C for 20 s, annealing for 20 s at 58°C, and extension at 65°C for 30 s with a final extension at 65°C for 7 min.

### Gene model predictions

Several forms of evidence were used as input into the MAKER annotation pipeline, including previous annotation of MdBV genes on proviral segments [43], assembled transcripts from *M. demolitor* and *P. includens* (see above) [28], [51], unassembled read mapping information, and protein sequences from other insect species [84]. The assembled transcripts were generated from *M. demolitor* ovaries, whole larvae, teratocytes and venom gland transcriptomes as described above. These data sets generated 113, 106, 107, and 147 million 100 bp paired reads respectively, which assembled into 36,891 transcripts with lengths greater than 500 bp and overall abundance greater than 5 Reads Per Kilobase of exon model per Million reads mapped (RPKM). Of 51 million quality filtered Illumina read pairs from ovaries, 11% were successfully mapped to the 40 scaffolds containing MdBV proviral genome elements. Unassembled read mapping information was generated via mapping wasp ovary and *C. includens* hemocyte transcriptome reads against the scaffolds of interest using tophat and cufflinks [85]. The tophat read junctions file (mapping splice sites) and cufflinks transcripts file were converted into gff3 format using scripts bundled with MAKER, tophat2gff3 and cufflinks2gff3. Protein sequences combined all coding sequences from the *Nasonia vitripennis* and *Apis mellifera* genomes, all known MdBV coding sequences, BV nudivirus-like coding sequences from *M. demolitor, Cotesia congregata*, and *Chelonus inanitus*, as well as all predicted ORFs from the genome scaffolds (without introns) >500 bp in size [27], [28], [36], [63], [86].

The *de novo* predictors GeneMark-ES, Augustus, and SNAP were also used within MAKER [87]–[89]. A GeneMark-ES model file was made using gm_es.pl and the scaffolds of interest as input. For Augustus prediction, a set of training genes was made by running Augustus on the scaffolds of interest with the species model “Nasonia” in addition to an intron position “hints” file generated with bam2hints from ovary transcriptome reads mapped by tophat. A “Microplitis” species file was made using the Augustus training gene set and the set of assembled transcripts described above with the autoAug.pl script.

SNAP training was performed by iteratively running MAKER according to the “Training *ab initio* Gene Predictors” section of the MAKER tutorial, using assembled transcripts in the first iteration as evidence for gene models. The final MAKER run used the GeneMark-ES model file, the Augustus species file “Microplitis” and a SNAP model file generated by two rounds of training. Repeat masking was performed with default options.

The resulting MAKER gff3 files were loaded into Apollo genome browser and gene models were manually edited if necessary [90]. All gene models were exported into Genbank table format. The coding sequences were assigned putative functional roles based upon BLAST results from the NCBI nr database, the *Drosophila melanogaster* set of protein-coding genes, and HMMER hmmsearch results from the PFAM and TIGRFAM databases [91], [92]. tRNAs were identified using Aragorn [93]. These results were combined into GenBank format using custom perl scripts and tbl2asn from NCBI. The resulting dataset was submitted as BioProject 195937 and assigned GenBank accession number AZMT000000000. Altogether, we identified 1,737 genes in the 40 scaffolds containing MdBV components of which 1,713 were predicted protein-coding sequences and 24 were tRNAs (Table S1).

### Annotation of sequence motifs

Wasp Integration Motifs (WIMs) share the common sequence AGCT and identify the site at which MdBV proviral segments excise from the *M. demolitor* genome [35]. WIMs were previously mapped for three segments by inverse PCR [35]. The locations of WIM sites for all proviral segments in the *M. demolitor* genome were obvious from read mapping data, and segment beginning and end coordinates were given to AGCT sites where circularization occurs. Host Integration Motifs (HIMs) were also identified previously by PCR methods and sequencing as the site where MdBV DNAs in virions integrate into the genome of infected host cells [35]. The twelve HIM sequences identified by these approaches were aligned and made into a Hidden Markov Model (hmm) using HMMER hmmbuild (http://hmmer.janelia.org). This hmm was used as a query in a HMMER hmmsearch against the scaffolds of interest to identify HIM motifs in all correctly assembled old and new segments. Sequence motifs were aligned using MUSCLE and maximum likelihood phylogenetic trees were built using phylogeny.fr with the HKY+I+G model and 100 bootstrap replicates [94].

### Analyses of syntenic regions with other BV carrying wasps

Sets of orthologous genes were identified using orthomcl and four datasets: 1) all protein-coding sequences from *M. demolitor*, 2) all protein sequences from *G. flavicoxis* BV segments and flanking regions of the wasp genome, 3) same information from 2 for *G. indiensis*, and 4) protein coding sequences from nudivirus-gene containing regions of *C. congregata* [27], [41], [95]. Groups of orthologous genes (syntenic) in the genome were identified using the orthomcl output with orthocluster [96]. Syntenic regions were viewed with Gbrowse syn. Assembly of the *M. demolitor* genome initially suggested the nudivirus-like gene cluster contained two identical duplications absent from the nudivirus-like gene cluster identified in the wasp *C. congregata* [27]: *Cc50C22.3/HzNVorf94-like/38K* and *27b-like/Cc50C22.6*. Given the identical nature of these predicted duplications, we assessed whether they were correct by primer walking and Sanger sequencing these domains. Resequencing showed that these genes were not duplicated. The above information was then used to make figures that included modifications of pictorial representations of *M. demolitor* genes on scaffolds generated by Gbrowse [97], [98].

## Supporting Information

Figure S1 PCR verification of circularization of segments in MdBV virions. PCR products generated using primers (Table S1) that specifically amplify the single WIM formed after excision and circularization of MdBV proviral segments. Prior studies [43], [50] confirmed that circularized segments A-O are packaged into MdBV virions. This figure shows amplicons generated using WIM specific primers for each of the new segments identified in this study (K1, P-X). For each segment and WIM specific primer set, the following templates were examined: A) MdBV DNA extracted from virions pretreated with DNAse to remove exogenous DNA, B) whole ovary genomic DNA from Stage 1 female wasp pupae, which precedes MdBV replication [28], C) whole genomic DNA from adult male wasps, and D) negative control lacking MdBV template. Resulting products were visualized by ethidium bromide staining after running on agarose gels. Molecular mass markers are indicated to the left. Results show each circularized segment is present in virions as indicated by the presence of a PCR product of predicted size in lane A. Circularized segments are never observed in Stage 1 pupal ovaries, but sometimes are present in very low abundance using adult male DNA as template (segments A and B). Cloning and sequencing indicated the off-size products in lanes B and C for segments K1 and K are amplicons spanning repetitive WIM sequences in proviral DNA spanning segments P and K1 and K1 and K respectively.
(EPS)

Figure S2 Sequence similarity among MdBV proviral segments located in Locus 1 in the wasp genome. The segment-containing region of Locus 1 was aligned against itself using BLAST to identify regions of similarity (NCBI Genome Workbench with blastn, e-value cut-off = 0.001). The resulting dot matrix is colored to indicate proviral segment boundaries for segments that share some sequence similarity. Segments K1 and K show near total co-linearity in sequence suggesting recent duplication (dashed boxes labeled 1). Short regions of similarity are detected for select other tandem segment pairs including D and Q (boxes 2), A and B (boxes 3), and F and I (boxes 4), which may represent less recent duplication events.
(EPS)

Figure S3 qPCR and sequence read abundance generate similar estimates for the abundance of circularized segments in MdBV virions. Each point shows the estimated abundance for MdBV segments A-O as previously measured by quantitative PCR (x axis) [50] and sequence read coverage (y axis) as measured in this study. qPCR estimates were generated as absolute copy numbers generated from standard curves. Sequence read coverage was derived from mapping sequence reads as described in the Materials and Methods and is a relative metric that depends upon the total amount of sequencing undertaken. Each dot indicates a given segment with select segments (C, B, H, and J) identified. Although MdBV DNA isolated from virions was phi29 amplified prior to sequencing, the relative abundance of each segment estimated by read coverage is similar to qPCR estimates indicating that amplification did not have a large effect on sequence coverage. The red line indicates the best linear fit for the two estimates with the resulting correlation being highly significant (R^2^ = 0.8, p<0.0001).
(EPS)

Figure S4 Gene content of MdBV proviral segments. Proviral segments are organized into loci and oriented as described in Figure 1. The relative size and location of identified genes on each proviral segment. Each identified gene is named above or below its location. Color coding of boxes indicate exons and identify whether a given gene is a member of a particular family, is similar to an orphan gene identified from another BV, or is unknown. Lines connecting boxes indicate introns in a given gene, while white boxes indicate untranslated regions. Specific coordinates on scaffolds for each gene shown in the figure are summarized in Table S1.
(EPS)

Table S1 Coordinates and descriptions of all genes located in 40 scaffolds containing components of the *M. demolitor* proviral genome. Each row in the table contains an annotated gene, its location on scaffolds or contigs, the start and end position including untranslated regions, the NCBI locus tag, gene name, notes and description of putative function. Rows containing genes on different scaffolds are separated by a double line border. Nudivirus-like genes are highlighted in bold red type, while genes on proviral segments are indicated in bold type and in cells colored by segment location using the color key for segments in Figure 1. Genes indicated in black type consist primarily of predicted *M. demolitor* genes that are unrelated to viral functions.
(PDF)

Table S2 Genes or gene families shared between nudivirus-like and proviral segment regions of the genome.
(PDF)

Table S3 PCR primers used in this study.
(PDF)
