# Supplementary material for: Widespread Genome Reorganization of an Obligate Virus Mutualist
Source: PLoS Genet. 2014 Sep 18;10(9):e1004660. doi: 10.1371/journal.pgen.1004660 (PMC4169385; doi:10.1371/journal.pgen.1004660)
Supplement: Table S1 — Coordinates and descriptions of all genes located in 40 scaffolds containing components of the M. demolitor proviral genome. Each row in the table contains an annotated gene, its location on scaffolds or contigs, the start and end position including untranslated regions, the NCBI locus tag, gene name, notes and description of putative function. Rows containing genes on different scaffolds are separated by a double line border. Nudivirus-like genes are highlighted in bold red type, while genes on proviral segments are indicated in bold type and in cells colored by segment location using the color key for segments in Figure 1. Genes indicated in black type consist primarily of predicted M. demolitor genes that are unrelated to viral functions. (PDF) [file pgen.1004660.s005.pdf]

Scaffold	Start	End	Locus tag	Gene symbol	Note	Description
**Mdem_contig_4062422**	**1**	**1286**	**K425_1**	**odv-e66-1**		**putative envelope protein ODV-E66-1**
**Mdem_contig_4124537**	**768**	**1203**	**K425_2**	**orph-D2**		**Segment D hypothetical protein**
**Mdem_contig_4124537**	**2205**	**2528**	**K425_3**	**orph-D3**		**Segment D hypothetical protein**
Mdem_contig_4139115	1	14134	K425_4			putative cytoplasmic dynein 2 heavy chain 1-like protein
**Mdem_contig_4139115**	**14435**	**15375**	**K425_5**	**odv-e66-2**		**putative envelope protein ODV-E66-2**
**Mdem_scaffold_0004**	**1**	**597**	**K425_1373**	**odv-e66-9**	**similar to GFP_L7_0780, GIP_L8_0020**	**putative envelope protein ODV-E66-9, partial**
**Mdem_scaffold_0004**	**1726**	**4238**	**K425_1374**	**odv-e66-10**	**similar to GFP_L7_0780, GIP_L8_0020**	**putative envelope protein ODV-E66-10**
Mdem_scaffold_0004	9277	14712	K425_1499			eye-specific diacylglycerol kinase-like protein
Mdem_scaffold_0004	34280	45157	K425_1498			upheld-like protein
Mdem_scaffold_0004	46767	47447	K425_1497			U11/U12 small nuclear ribonucleoprotein 35 kDa-like protein
Mdem_scaffold_0004	47873	53813	K425_1496			protein tyrosine kinase domain-containing protein
Mdem_scaffold_0004	55469	57942	K425_1495			conserved uncharacterized protein
Mdem_scaffold_0004	58198	62790	K425_1375			vesicular integral-membrane protein VIP36-like protein
Mdem_scaffold_0004	63801	63872	K425_1370			tRNA-Glu
Mdem_scaffold_0004	64451	68498	K425_1376			wolframin-like protein
Mdem_scaffold_0004	68791	69726	K425_1494			ankyrin repeat domain-containing protein
Mdem_scaffold_0004	70888	70959	K425_1372			tRNA-Glu
Mdem_scaffold_0004	71082	72695	K425_1378			mitochondrial ribosomal protein L22
Mdem_scaffold_0004	72669	74473	K425_1493			ribosomal protein L39
Mdem_scaffold_0004	75984	86628	K425_1377			lipase 3-like protein
Mdem_scaffold_0004	89711	93208	K425_1379			lipase 3-like protein
Mdem_scaffold_0004	95721	101535	K425_1380			lipase 3-like protein
Mdem_scaffold_0004	105894	117625	K425_1381			low-density lipoprotein receptor-related-like protein
Mdem_scaffold_0004	119695	123776	K425_1492			Sorting nexin-like protein
Mdem_scaffold_0004	126150	128656	K425_1491			Corticotropin-releasing factor-binding protein
Mdem_scaffold_0004	136952	137522	K425_1490			conserved uncharacterized protein
Mdem_scaffold_0004	140571	146006	K425_1489			Armadillo/beta-catenin-like repeat-containing protein
Mdem_scaffold_0004	147968	154334	K425_1382			dicer2-like protein
Mdem_scaffold_0004	157801	160213	K425_1383			Proteasome subunit alpha type-1
Mdem_scaffold_0004	160782	163644	K425_1488			Lipoyl synthase, mitochondrial
Mdem_scaffold_0004	163645	166702	K425_1487			trypsin domain-containing protein
Mdem_scaffold_0004	170785	173910	K425_1486			F-box containing-protein
Mdem_scaffold_0004	178473	181437	K425_1384			hypothetical protein
Mdem_scaffold_0004	182223	187763	K425_1385			Coiled-coil domain-containing protein 22-like protein
Mdem_scaffold_0004	187783	189692	K425_1485			Mitochondrial 28S ribosomal protein S27 domain-containing protein
Mdem_scaffold_0004	193626	198688	K425_1386			phosphoribosylformylglycinamidine synthase
Mdem_scaffold_0004	199833	200950	K425_1387			KIAA1430-like protein
Mdem_scaffold_0004	201881	205399	K425_1388		similar to GIP_L7_0080, GFP_L5_0240, GIP_L2_0090, GFP_L5_0230, GFP_L5_0070, GFP_L5_0090, GIP_L2_0100, GIP_L7_0040, GIP_L7_0010, and GFP_L5_0190	conserved uncharacterized protein family 7
Mdem_scaffold_0004	208028	215623	K425_1484			transmembrane protein 131-like protein
Mdem_scaffold_0004	217870	223911	K425_1389			nucleosome assembly protein 1-like protein
Mdem_scaffold_0004	224725	229767	K425_1390			apoptosis-inducing factor 1, mitochondrial-like protein
Mdem_scaffold_0004	232035	234213	K425_1482			Broad-minded domain-containing protein
Mdem_scaffold_0004	235112	242403	K425_1483			TBC domain-containing protein kinase-like protein
Mdem_scaffold_0004	247413	247484	K425_1371			tRNA-Glu
Mdem_scaffold_0004	248706	251103	K425_1481			tex-like protein
Mdem_scaffold_0004	250633	252501	K425_1391			structure-specific endonuclease subunit slx1-like protein
Mdem_scaffold_0004	253187	256399	K425_1392			ribosomal protein S30-like protein
Mdem_scaffold_0004	259039	263422	K425_1393			conserved uncharacterized protein family 3
Mdem_scaffold_0004	265275	273681	K425_1394			conserved uncharacterized protein family 3
Mdem_scaffold_0004	273733	275802	K425_1480			cytochrome P450 domain-containing protein
Mdem_scaffold_0004	281432	286369	K425_1479			ras-like protein 2-like protein
Mdem_scaffold_0004	287947	290056	K425_1395			centrin-2-like protein
Mdem_scaffold_0004	290481	293170	K425_1478			exosome complex component RRP43-like protein
Mdem_scaffold_0004	294032	296193	K425_1396			Signal peptidase complex subunit 3
Mdem_scaffold_0004	297419	303180	K425_1397			mediator complex subunit 25-like protein
Mdem_scaffold_0004	305603	310229	K425_1477			mediator complex subunit 25-like protein
Mdem_scaffold_0004	310559	315691	K425_1398			protein transport protein Sec24C-like protein
Mdem_scaffold_0004	317949	323780	K425_1399			heparan-alpha-glucosaminide N-acetyltransferase-like protein
Mdem_scaffold_0004	338137	355158	K425_1476			glutamate receptor, ionotropic kainate 2-like protein
Mdem_scaffold_0004	382444	393240	K425_1435			protein spitz-like protein
Mdem_scaffold_0004	400134	403486	K425_1400			bride of sevenless-like protein
Mdem_scaffold_0004	408797	412496	K425_1401			MFS sugar transporter domain-containing protein
Mdem_scaffold_0004	414043	421025	K425_1402			Dual 3',5'-cyclic-AMP and -GMP phosphodiesterase 11
Mdem_scaffold_0004	423339	426041	K425_1475			golgin family, subfamily A member 7-like protein
Mdem_scaffold_0004	426498	429654	K425_1403			Galactoside 2-alpha-L-fucosyltransferase 3
Mdem_scaffold_0004	431475	434272	K425_1474			growth arrest specific protein 8-like protein
Mdem_scaffold_0004	435109	442008	K425_1473			pantothenate kinase
Mdem_scaffold_0004	442219	444821	K425_1404			serine--tRNA ligase
Mdem_scaffold_0004	449637	453788	K425_1472			growth arrest-specific protein 8-like protein
Mdem_scaffold_0004	454012	461145	K425_1405			conserved uncharacterized protein
Mdem_scaffold_0004	461535	463948	K425_1406			Oxysterol-binding protein-related protein 11
Mdem_scaffold_0004	466342	471548	K425_1471		similar to hypothetical protein Cotesia conregata bracovirus CcBV_23.4, GIP_L1_00360, and GFP_L7_0430	ribonuclease T2 domain-containing protein
Mdem_scaffold_0004	471946	475156	K425_1407			PDZ domain-containing protein
Mdem_scaffold_0004	475160	479405	K425_1470			p53-like protein
Mdem_scaffold_0004	480840	483559	K425_1469			putative palmitoyltransferase ZDHHC16-like protein

Mdem_scaffold_0004	484112	486322	K425_1408			mitochondrial ribosomal protein S26
Mdem_scaffold_0004	487979	498157	K425_1468			mothers against decapentaplegic 4-like protein
Mdem_scaffold_0004	500345	505387	K425_1467			ribonuclease T2 domain-containing protein
Mdem_scaffold_0004	505815	510115	K425_1466			ralA-binding-like protein
Mdem_scaffold_0004	512973	517555	K425_1409			glucose dehydrogenase acceptor-like protein
Mdem_scaffold_0004	517186	525656	K425_1465			EB module-containing protein
Mdem_scaffold_0004	527692	535446	K425_1464			EB module-containing protein
Mdem_scaffold_0004	536491	538954	K425_1410			EB module-containing protein
Mdem_scaffold_0004	540970	545107	K425_1411			upstream activation factor subunit spp27-like protein
Mdem_scaffold_0004	547362	553441	K425_1412			putative protein phosphatase 2C
Mdem_scaffold_0004	549582	550675	K425_1463			hypothetical protein
Mdem_scaffold_0004	553909	559264	K425_1413			TAPT1-like protein
Mdem_scaffold_0004	560343	564064	K425_1414			GTP-binding protein 2-like protein
Mdem_scaffold_0004	565321	569856	K425_1462			serine protease snake-like protein
Mdem_scaffold_0004	570653	577724	K425_1415			bifunctional protein NCOAT-like protein
Mdem_scaffold_0004	578167	582827	K425_1461			leucine-rich repeat-containing protein
Mdem_scaffold_0004	586785	597802	K425_1460			neurotactin-like protein
Mdem_scaffold_0004	599015	604921	K425_1416			tankyrase-1-like protein
Mdem_scaffold_0004	611131	614356	K425_1417			protein phosphatase 1 regulatory subunit 3C-B-like protein
Mdem_scaffold_0004	615131	617459	K425_1459			serine protease snake-like protein
Mdem_scaffold_0004	620209	621382	K425_1458			WD repeat domain-containing protein
Mdem_scaffold_0004	621722	625510	K425_1418			Non-specific lipid-transfer protein
Mdem_scaffold_0004	625638	627986	K425_1419			CDC42 small effector-like protein
Mdem_scaffold_0004	629085	632092	K425_1457			another transcription unit-like protein
Mdem_scaffold_0004	635331	665346	K425_1420			tight junction protein ZO-1-like protein
Mdem_scaffold_0004	642744	645310	K425_1456			MFS family sugar transporter
Mdem_scaffold_0004	666544	669783	K425_1421			Coiled-coil and C2 domain-containing protein 2A
Mdem_scaffold_0004	671089	675610	K425_1455			elongation of very long chain fatty acids protein 4-like protein
Mdem_scaffold_0004	686247	691084	K425_1422			glycine receptor subunit alpha-1-like protein
Mdem_scaffold_0004	691226	697324	K425_1454			periodic tryptophan protein 2-like protein
Mdem_scaffold_0004	707839	720776	K425_1453			eukaryotic translation initiation factor 4 gamma 2-like protein
Mdem_scaffold_0004	721111	724168	K425_1423			syntaxin-12-like protein
Mdem_scaffold_0004	725723	735594	K425_1452			ankyrin repeat and BTB/POZ domain-containing protein BTBD11-like protein
Mdem_scaffold_0004	745662	752237	K425_1424			Proton-coupled amino acid transporter-like protein
Mdem_scaffold_0004	777615	785307	K425_1425			Paired box protein Pax-6-like protein
Mdem_scaffold_0004	791633	793038	K425_1451			conserved uncharacterized protein
Mdem_scaffold_0004	802342	804959	K425_1426			Paired box protein Pax-6-like protein
Mdem_scaffold_0004	806160	818745	K425_1450			conserved uncharacterized protein
Mdem_scaffold_0004	819353	840921	K425_1427			centrosomal protein of 135 kDa-like protein
Mdem_scaffold_0004	826536	830290	K425_1449			conserved uncharacterized protein
Mdem_scaffold_0004	841268	858230	K425_1448			conserved uncharacterized protein
Mdem_scaffold_0004	859232	862705	K425_1428			Seryl-tRNA synthetase, mitochondrial
Mdem_scaffold_0004	879083	880323	K425_1447			Dynein heavy chain 1, axonemal-like protein
Mdem_scaffold_0004	886039	890070	K425_1429			mps one binder kinase activator-like protein 2-like protein
Mdem_scaffold_0004	890884	892531	K425_1446			conserved uncharacterized protein
Mdem_scaffold_0004	893910	898483	K425_1445			Palmitoyltransferase and ankyrin-repeat containing protein
Mdem_scaffold_0004	928132	931779	K425_1430			CD80-like C2-set immunoglobulin domain-containing protein
Mdem_scaffold_0004	939829	943659	K425_1444			peptidyl-prolyl cis-trans isomerase FKBP4-like protein
Mdem_scaffold_0004	945563	950360	K425_1431			squamous cell carcinoma antigen recognized by T-cells 3-like protein
Mdem_scaffold_0004	950030	953021	K425_1443			conserved uncharacterized protein
Mdem_scaffold_0004	956002	958931	K425_1442			conserved uncharacterized protein
Mdem_scaffold_0004	959124	961704	K425_1432			zinc metalloproteinase nas-like protein
Mdem_scaffold_0004	961692	973758	K425_1441			glucose dehydrogenase acceptor-like protein
**Mdem_scaffold_0004**	**974198**	**977563**	**K425_1440**	**35a-6**	**Similar to Chelonus inanitus 35a**	**conserved uncharacterized protein 35a-like protein-6**
**Mdem_scaffold_0004**	**976602**	**982351**	**K425_1439**	**35a-7**	**Similar to Chelonus inanitus 35a**	**conserved uncharacterized protein 35a-like protein-7**
Mdem_scaffold_0004	982606	985440	K425_1438			hypothetical protein
Mdem_scaffold_0004	986306	989669	K425_1437			clavesin-2-like protein
Mdem_scaffold_0004	997381	1003705	K425_1433			olfactory receptor
Mdem_scaffold_0004	1008583	1013104	K425_1434			ras-like protein family member 10B-like protein
Mdem_scaffold_0004	1013456	1014295	K425_1436			cell growth-regulating nucleolar-like protein
Mdem_scaffold_0014	197	4173	K425_248			conserved uncharacterized protein
Mdem_scaffold_0014	3725	6022	K425_408			conserved uncharacterized protein
Mdem_scaffold_0014	6577	11422	K425_409			putative ribosomal RNA methyltransferase-like protein
Mdem_scaffold_0014	16282	16826	K425_407			7tm Odorant receptor domain-containing protein
Mdem_scaffold_0014	17127	18991	K425_406			7tm Odorant receptor domain-containing protein
Mdem_scaffold_0014	28115	37269	K425_249			cln3-like protein
Mdem_scaffold_0014	38118	41592	K425_405			actin domain-containing protein
Mdem_scaffold_0014	43020	80327	K425_404			Down syndrome cell adhesion molecule-like protein
Mdem_scaffold_0014	94540	94893	K425_403			Down syndrome cell adhesion molecule-like protein
Mdem_scaffold_0014	95599	98277	K425_250			hypothetical protein
Mdem_scaffold_0014	195639	199692	K425_251			Zinc finger, C2H2 type domain-containing protein
Mdem_scaffold_0014	220542	226059	K425_252			solute carrier family-like protein
Mdem_scaffold_0014	230341	233934	K425_253			kelch-like protein
Mdem_scaffold_0014	232571	236582	K425_402			protein phosphatase methylesterase 1-like protein
Mdem_scaffold_0014	244250	248898	K425_254			Immunoglobulin domain-containing protein
Mdem_scaffold_0014	252054	253309	K425_401			HCaRG domain-containing protein
Mdem_scaffold_0014	253668	259750	K425_255			conserved uncharacterized protein
Mdem_scaffold_0014	260141	260946	K425_400			methyltransferase domain-containing protein
Mdem_scaffold_0014	272388	282130	K425_399			methyltransferase domain-containing protein
Mdem_scaffold_0014	298396	301457	K425_398			hypothetical protein
Mdem_scaffold_0014	314709	315437	K425_397			Zinc finger, C2H2 type domain-containing protein
Mdem_scaffold_0014	369213	371216	K425_256			hypothetical protein

Mdem_scaffold_0014	377853	390994	K425_396			zinc finger homeobox-like protein
Mdem_scaffold_0014	465304	465753	K425_257			hypothetical protein
Mdem_scaffold_0014	638238	640332	K425_258			ADP-ribosylation factor 2-like protein
Mdem_scaffold_0014	641759	645797	K425_395			conserved uncharacterized protein
Mdem_scaffold_0014	647900	650698	K425_259			F-box domain-containing protein
Mdem_scaffold_0014	650863	654361	K425_394			PDZ domain-containing protein 6-like protein
Mdem_scaffold_0014	654844	657791	K425_393			fizzy-like protein
Mdem_scaffold_0014	706572	711711	K425_392			synaptogyrin-like protein
Mdem_scaffold_0014	712215	715696	K425_260			RNA-binding protein squid-like protein
Mdem_scaffold_0014	718722	725613	K425_261			Protein tyrosine kinase domain-containing protein
Mdem_scaffold_0014	718904	720780	K425_391			short-chain dehydrogenase domain-containing protein
Mdem_scaffold_0014	726088	727284	K425_262			ribosomal protein L18A
Mdem_scaffold_0014	727780	730274	K425_263			F-box domain-containing protein
Mdem_scaffold_0014	731120	737560	K425_264			conserved uncharacterized protein
Mdem_scaffold_0014	739210	742384	K425_390			Zinc finger, C2H2 type domain-containing protein
Mdem_scaffold_0014	742389	743275	K425_265			hypothetical protein
Mdem_scaffold_0014	743648	747468	K425_266			conserved uncharacterized protein
Mdem_scaffold_0014	752856	762361	K425_267			Plexin cytoplasmic RasGAP domain-containing protein
Mdem_scaffold_0014	766535	774902	K425_389			integrator 12-like protein
Mdem_scaffold_0014	773062	781838	K425_268			conserved uncharacterized protein
Mdem_scaffold_0014	782873	785459	K425_388			Zn-finger in Ran binding protein and others domain-containing protein
Mdem_scaffold_0014	799108	801547	K425_387			Neurotransmitter-gated ion-channel transmembrane region-containing protein
Mdem_scaffold_0014	929350	930107	K425_269			hypothetical protein
Mdem_scaffold_0014	932620	933803	K425_386			Steroid receptor RNA activator (SRA1) domain-containing protein
Mdem_scaffold_0014	935431	947317	K425_270			C2 domain-containing protein
Mdem_scaffold_0014	947898	949303	K425_385			DNA/RNA non-specific endonuclease domain-containing protein
Mdem_scaffold_0014	951228	951964	K425_384			myosuppressin-like protein
Mdem_scaffold_0014	963571	967587	K425_271			AMP-binding enzyme-like protein
Mdem_scaffold_0014	967580	975739	K425_383			exosome complex exonuclease RRP44-like protein
Mdem_scaffold_0014	974813	978896	K425_272			nudE Nuclear distribution-like protein
Mdem_scaffold_0014	982377	984941	K425_382			histone H3.3
Mdem_scaffold_0014	986991	989768	K425_381			histone H3.3
Mdem_scaffold_0014	990006	993493	K425_273		similar to GFP_L1_0140 and GIP_L5_0190	smad nuclear-interacting-like protein
Mdem_scaffold_0014	994802	1004140	K425_380		similar to GFP_L1_0120 and GIP_L5_0170	protein tyrosine kinase
Mdem_scaffold_0014	1005656	1014850	K425_379			protein tyrosine kinase
Mdem_scaffold_0014	1017699	1019900	K425_274		similar to GFP_L1_0100 and GIP_L5_0150	prolylcarboxypeptidase
Mdem_scaffold_0014	1020252	1024380	K425_378		similar to GFP_L1_0090 and GIP_L5_0140	kinesin-like protein KIF3A
**Mdem_scaffold_0014**	**1025619**	**1027172**	**K425_377**	**ank-N4**	**synonym IkB-N2**	**viral ankyrin**
**Mdem_scaffold_0014**	**1029536**	**1030027**	**K425_376**	**ank-N5**	**synonym IkB-N3**	**viral ankyrin**
**Mdem_scaffold_0014**	**1031018**	**1031953**	**K425_275**	**orph-N2**		**hypothetical protein**
**Mdem_scaffold_0014**	**1032789**	**1033748**	**K425_375**	**ptp-N3**		**protein tyrosine phosphatase**
**Mdem_scaffold_0014**	**1034610**	**1035572**	**K425_374**	**ptp-N4**		**protein tyrosine phosphatase**
**Mdem_scaffold_0014**	**1038217**	**1038523**	**K425_373**	**orph-N1**		**hypothetical protein**
**Mdem_scaffold_0014**	**1038613**	**1039101**	**K425_372**	**ank-N1**	**synonym IkB-N1**	**viral ankyrin**
**Mdem_scaffold_0014**	**1039430**	**1040368**	**K425_371**	**ptp-N1**		**protein tyrosine phosphatase**
**Mdem_scaffold_0014**	**1040865**	**1041809**	**K425_370**	**ptp-N2**		**protein tyrosine phosphatase**
**Mdem_scaffold_0014**	**1047523**	**1048047**	**K425_369**	**ank-J4**	**synonym IkB-J1**	**viral ankyrin**
**Mdem_scaffold_0014**	**1049185**	**1050078**	**K425_368**	**ptp-J2**		**protein tyrosine phosphatase**
**Mdem_scaffold_0014**	**1053924**	**1054805**	**K425_366**	**ptp-J3**		**protein tyrosine phosphatase**
**Mdem_scaffold_0014**	**1055620**	**1056519**	**K425_367**	**ptp-J4**		**protein tyrosine phosphatase**
**Mdem_scaffold_0014**	**1057637**	**1059777**	**K425_276**	**ptp-J1**		**protein tyrosine phosphatase**
Mdem_scaffold_0014	1062797	1065193	K425_365		similar to GFP_L1_0020 and GIP_L5_0010	conserved hypothetical protein
Mdem_scaffold_0014	1068563	1073033	K425_277		similar to GFP_L1_0010 and GIP_L5_0020	conserved hypothetical protein
Mdem_scaffold_0014	1073707	1077046	K425_278		similar to GIP_L5_0010	ras-related protein Rab-2
Mdem_scaffold_0014	1079986	1085208	K425_279			sorting nexin-6-like protein
Mdem_scaffold_0014	1095803	1101542	K425_364			Immunoglobulin domain-containing protein
Mdem_scaffold_0014	1110276	1112678	K425_280			Mitochondrial ATP synthase B chain precursor domain-containing protein
Mdem_scaffold_0014	1112800	1117695	K425_281			Adenosine deaminase-like protein
Mdem_scaffold_0014	1121196	1130673	K425_363			dead ringer-like protein
Mdem_scaffold_0014	1175983	1178245	K425_362			dead ringer-like protein
Mdem_scaffold_0014	1216215	1220056	K425_361			Bardet-Biedl syndrome 1 protein
Mdem_scaffold_0014	1218807	1225103	K425_282			Targeting protein for Xklp2 (TPX2) domain-containing protein
Mdem_scaffold_0014	1222170	1227805	K425_360			dispatched-like protein
Mdem_scaffold_0014	1231418	1237614	K425_283			sodium- and chloride-dependent GABA transporter-like protein
Mdem_scaffold_0014	1240865	1246393	K425_359			contactin-like protein
Mdem_scaffold_0014	1250329	1260118	K425_284			guanine nucleotide-binding protein subunit gamma-e-like protein
Mdem_scaffold_0014	1275945	1288224	K425_285			putative chitinase
Mdem_scaffold_0014	1287714	1299612	K425_358			chaoptin-like protein
Mdem_scaffold_0014	1314790	1330869	K425_286			NADPH oxidase-like protein
Mdem_scaffold_0014	1329088	1339961	K425_357			Diacylglycerol kinase epsilon-like protein
Mdem_scaffold_0014	1340521	1345389	K425_356			van gogh-like protein
Mdem_scaffold_0014	1346022	1349626	K425_287			Zinc finger, C2H2 type domain-containing protein
Mdem_scaffold_0014	1355390	1372447	K425_288			DNA mismatch repair protein Msh2-like protein
Mdem_scaffold_0014	1356138	1360977	K425_355			conserved uncharacterized protein
Mdem_scaffold_0014	1371328	1375019	K425_354			ras-related protein Rab-39B-like protein

Mdem_scaffold_0014	1375197	1379870	K425_353			xmas-2-like protein
Mdem_scaffold_0014	1380153	1382890	K425_289			sarah-like protein
Mdem_scaffold_0014	1384646	1389400	K425_352			cullin-5-like protein
Mdem_scaffold_0014	1389700	1392784	K425_351			endoplasmic reticulum oxidoreductin-1-like protein
Mdem_scaffold_0014	1393403	1396693	K425_290			ubiquitin-protein ligase E3 Mdm2-like protein
Mdem_scaffold_0014	1396595	1399308	K425_291			prenyl protease type I-like protein
Mdem_scaffold_0014	1399411	1402436	K425_350			Dihydroxyacetone kinase-like protein
Mdem_scaffold_0014	1403550	1410734	K425_292			monocarboxylate MFS transporter 10-like protein
Mdem_scaffold_0014	1417414	1419316	K425_293			sine oculis-binding-like protein
Mdem_scaffold_0014	1419691	1421958	K425_349			guanine nucleotide-binding protein subunit beta-like protein
Mdem_scaffold_0014	1427148	1428835	K425_348			photoreceptor-specific nuclear receptor-like protein
Mdem_scaffold_0014	1428739	1430583	K425_294			centrosomal protein of 78 kDa-like protein
Mdem_scaffold_0014	1431028	1431507	K425_347			photoreceptor-specific nuclear receptor-like protein
Mdem_scaffold_0014	1432644	1435057	K425_346			photoreceptor-specific nuclear receptor-like protein
Mdem_scaffold_0014	1437806	1441163	K425_345			photoreceptor-specific nuclear receptor-like protein
Mdem_scaffold_0014	1442144	1449075	K425_295			Inhibitor of Apoptosis domain-containing protein
Mdem_scaffold_0014	1443084	1447710	K425_344			mediator of RNA polymerase II transcription subunit 24-like protein
Mdem_scaffold_0014	1450715	1452220	K425_296			MFS sugar transporter-like protein
Mdem_scaffold_0014	1454323	1461293	K425_297			ankyrin repeat containing-protein
Mdem_scaffold_0014	1461426	1465891	K425_343			serine/threonine-protein kinase/endoribonuclease IRE1-like protein
Mdem_scaffold_0014	1466250	1467675	K425_298			RNA exonuclease 4-like protein
Mdem_scaffold_0014	1468713	1470876	K425_299			Flavin-containing monooxygenase-like protein
Mdem_scaffold_0014	1471349	1473619	K425_300			small glutamine-rich tetratricopeptide containing protein
Mdem_scaffold_0014	1474198	1479593	K425_301			Ras-like GTP-binding protein Rho1-like protein
Mdem_scaffold_0014	1484140	1494167	K425_342			neurofibromin-like protein
Mdem_scaffold_0014	1495565	1498465	K425_341			inositol-pentakisphosphate 2-kinase-like protein
Mdem_scaffold_0014	1504808	1505416	K425_340			BTG domain-containing protein
Mdem_scaffold_0014	1509593	1512283	K425_302			hypothetical protein
Mdem_scaffold_0014	1520152	1540197	K425_303			voltage-gated calcium channel alpha 1 subunit-like protein
Mdem_scaffold_0014	1540309	1548010	K425_304			poly(A) polymerase gamma-like protein
Mdem_scaffold_0014	1540310	1544433	K425_339			DnaJ domain-containing protein
Mdem_scaffold_0014	1550148	1557592	K425_305			fibrillin-like protein
Mdem_scaffold_0014	1557680	1561652	K425_338			polo-like protein
Mdem_scaffold_0014	1562311	1563926	K425_337			serine/threonine-protein kinase PLK2-like protein
Mdem_scaffold_0014	1565338	1569374	K425_306			pleckstrin domain-containing family M member
Mdem_scaffold_0014	1568795	1571338	K425_336			spindle assembly abnormal 6-like protein
Mdem_scaffold_0014	1571572	1573376	K425_307			UPF0760 protein C2orf29-like protein
Mdem_scaffold_0014	1573919	1576742	K425_308			conserved uncharacterized protein
Mdem_scaffold_0014	1577187	1577927	K425_335			BTB/POZ domain-containing protein
Mdem_scaffold_0014	1578857	1581465	K425_334			ATP synthase F1, delta subunit
Mdem_scaffold_0014	1580616	1582735	K425_309			conserved uncharacterized protein
Mdem_scaffold_0014	1583539	1587357	K425_333			Chitin binding Peritrophin-A domain-containing protein
Mdem_scaffold_0014	1594411	1603522	K425_310			F-box domain-containing protein
Mdem_scaffold_0014	1599992	1602126	K425_332			AMY-1-associating protein expressed in testis 1-like protein
Mdem_scaffold_0014	1602527	1605044	K425_331			conserved uncharacterized protein
Mdem_scaffold_0014	1609130	1611303	K425_311			thioredoxin domain-containing protein
Mdem_scaffold_0014	1616449	1620927	K425_330			dual specificity tyrosine-phosphorylation-regulated kinase 2-like protein
Mdem_scaffold_0014	1623122	1625614	K425_312			Autophagy-specific 8a-like protein
Mdem_scaffold_0014	1626044	1632321	K425_313			nuclear hormone receptor ligand-binding domain-containing protein
Mdem_scaffold_0014	1633101	1639667	K425_314			protein tyrosine kinase domain-containing protein
Mdem_scaffold_0014	1639770	1641701	K425_329			cytosolic carboxypeptidase 1-like protein
Mdem_scaffold_0014	1647633	1651632	K425_315			conserved uncharacterized protein
Mdem_scaffold_0014	1652213	1658172	K425_328			cytosolic carboxypeptidase 1-like protein
Mdem_scaffold_0014	1662791	1663708	K425_316			hypothetical protein
Mdem_scaffold_0014	1664799	1666921	K425_326			hypothetical protein
Mdem_scaffold_0014	1668281	1670554	K425_327			electron transfer flavoprotein subunit alpha, mitochondrial-like protein
Mdem_scaffold_0014	1671650	1673132	K425_317			DNA methyltransferase 1-associated protein 1-like protein
Mdem_scaffold_0014	1683209	1693694	K425_325			K+-dependent Na+/Ca+ exchanger-like protein
Mdem_scaffold_0014	1712204	1733726	K425_318			chondroitin sulfate proteoglycan 4-like protein
Mdem_scaffold_0014	1736339	1737561	K425_319			Pre-rRNA-processing protein TSR2-like protein
Mdem_scaffold_0014	1737131	1741672	K425_324			Niemann-Pick type C-1-like protein
Mdem_scaffold_0014	1742985	1745909	K425_320			hypothetical protein
Mdem_scaffold_0014	1745913	1749505	K425_323			CECR5, HAD hydrolase, TIGR01456 family
Mdem_scaffold_0014	1750646	1754213	K425_321			Atg14, UV radiation resistance protein and autophagy-related subunit 14 domain-containing protein
Mdem_scaffold_0014	1754066	1756814	K425_322			retinol dehydrogenase 11-like protein
Mdem_scaffold_0019	16721	17669	K425_552			conserved uncharacterized protein
Mdem_scaffold_0019	18681	21180	K425_551			reverse transcriptase-like protein
Mdem_scaffold_0019	22244	24229	K425_526			hypothetical protein
Mdem_scaffold_0019	27542	29329	K425_527			conserved uncharacterized protein
Mdem_scaffold_0019	64422	65264	K425_550			gag protein from human immunodeficiency virus 1-like protein
Mdem_scaffold_0019	74794	75531	K425_553			hypothetical protein
Mdem_scaffold_0019	78581	79816	K425_549			conserved uncharacterized protein
Mdem_scaffold_0019	84970	85920	K425_548			reverse transcriptase-like protein
**Mdem_scaffold_0019**	**93164**	**94400**	**K425_547**	**int-2**	**synonym HzNVORF144-like protein**	**viral integrase**
Mdem_scaffold_0019	100850	102709	K425_528			conserved uncharacterized protein
Mdem_scaffold_0019	118604	119401	K425_529		similar to GFP_L5_0200	conserved uncharacterized protein
Mdem_scaffold_0019	119668	121030	K425_530			hypothetical protein
Mdem_scaffold_0019	129033	129359	K425_531			hypothetical protein
Mdem_scaffold_0019	129390	129773	K425_532			hypothetical protein
Mdem_scaffold_0019	130795	131157	K425_533			hypothetical protein
Mdem_scaffold_0019	137002	139885	K425_546			hypothetical protein
Mdem_scaffold_0019	152231	153091	K425_534		similar to GFP_L5_0170	conserved uncharacterized protein

Mdem_scaffold_0019	166383	170089	K425_545			120.7 kDa protein in NOF-FB transposable element-like protein
Mdem_scaffold_0019	183576	183956	K425_535			hypothetical protein
Mdem_scaffold_0019	225326	226144	K425_544		similar to GFP_L5_0170	conserved uncharacterized protein family 1
Mdem_scaffold_0019	229942	231068	K425_536			hypothetical protein
Mdem_scaffold_0019	231421	232250	K425_537			hypothetical protein
Mdem_scaffold_0019	232399	233043	K425_538			hypothetical protein
Mdem_scaffold_0019	240043	241119	K425_539			retroviral aspartyl protease domain-containing protein
Mdem_scaffold_0019	242542	243765	K425_540			hypothetical protein
Mdem_scaffold_0019	248595	249749	K425_541		present in baculovirus and insect genomes	baculovirus envelope protein efp-like protein
Mdem_scaffold_0019	250127	250477	K425_542			hypothetical protein
Mdem_scaffold_0019	250706	253635	K425_543			hypothetical protein
Mdem_scaffold_0025	2974	12501	K425_602			anoctamin-4-like protein
Mdem_scaffold_0025	16611	32384	K425_749			Son of sevenless-like protein
Mdem_scaffold_0025	30972	39331	K425_603			bric-a-brac-like protein
Mdem_scaffold_0025	44714	49090	K425_604			Zinc finger, C2H2 type domain-containing protein
Mdem_scaffold_0025	55746	57965	K425_748			conserved uncharacterized protein
Mdem_scaffold_0025	60426	65031	K425_747			conserved uncharacterized protein
Mdem_scaffold_0025	66830	67578	K425_605			conserved uncharacterized protein
Mdem_scaffold_0025	103589	115476	K425_606			protein tyrosine kinase domain-containing protein
Mdem_scaffold_0025	116354	118422	K425_607			hypothetical protein
Mdem_scaffold_0025	123783	130512	K425_746			Alpha-N-acetylglucosaminidase
Mdem_scaffold_0025	131009	135474	K425_745			Endoribonuclease XendoU domain-containing protein
Mdem_scaffold_0025	137440	140155	K425_608			conserved uncharacterized protein
Mdem_scaffold_0025	140305	156371	K425_744			Haloacid dehalogenase-like hydrolase domain-containing protein
Mdem_scaffold_0025	182282	192990	K425_743			protein kinase C-binding NELL1-like protein
Mdem_scaffold_0025	261708	266677	K425_742			conserved uncharacterized protein
Mdem_scaffold_0025	266233	279332	K425_609			transporter, solute:sodium symporter (SSS) family
Mdem_scaffold_0025	289265	299509	K425_741			period-like protein
Mdem_scaffold_0025	316237	321868	K425_740			RNA-binding protein 45-like protein
Mdem_scaffold_0025	323356	333827	K425_610			procollagen lysyl hydroxylase-like protein
Mdem_scaffold_0025	326963	327951	K425_739			hemicentin-1-like protein
Mdem_scaffold_0025	329264	330672	K425_738			contactin-5-like protein
Mdem_scaffold_0025	335196	339602	K425_737			modifier of rudimentary-like protein
Mdem_scaffold_0025	342141	343587	K425_736			NEDD4 family-interacting-like protein
Mdem_scaffold_0025	345705	351552	K425_611			PWWP domain-containing protein
Mdem_scaffold_0025	362296	369986	K425_735			leucine-rich repeat and immunoglobulin-like protein domain-containing protein
Mdem_scaffold_0025	372275	374245	K425_612			Nuclear protein NP60-like protein
Mdem_scaffold_0025	401778	427927	K425_613			lilliputian-like protein
Mdem_scaffold_0025	447201	455641	K425_614			conserved uncharacterized protein
Mdem_scaffold_0025	458587	463227	K425_615			conserved uncharacterized protein
Mdem_scaffold_0025	466726	468857	K425_734			glucosidase 2 subunit beta-like protein
Mdem_scaffold_0025	469211	472015	K425_616			protein tumorous imaginal disc, mitochondrial-like protein
Mdem_scaffold_0025	472912	475386	K425_733			Ion channel domain-containing protein
Mdem_scaffold_0025	476673	480187	K425_617			N-acetyllactosaminide beta-1,3-N-acetylglucosaminyltransferase-like protein
Mdem_scaffold_0025	480466	486114	K425_732			wee-like protein kinase-like protein
Mdem_scaffold_0025	485932	487982	K425_618			prenylated Rab acceptor protein 1-like protein
Mdem_scaffold_0025	488474	493577	K425_619			anoctamin-1-like protein
Mdem_scaffold_0025	494275	496658	K425_622			60S ribosomal export protein NMD3
Mdem_scaffold_0025	497109	499713	K425_621			sorting and assembly machinery component 50-like protein
Mdem_scaffold_0025	500059	503593	K425_620			Protein phosphatase 1 regulatory subunit-like protein
Mdem_scaffold_0025	507040	508812	K425_623			conserved uncharacterized protein
Mdem_scaffold_0025	514247	515675	K425_624			myotrophin-like protein
Mdem_scaffold_0025	515089	521002	K425_731			histone-lysine N-methyltransferase SET-like protein
Mdem_scaffold_0025	520700	523893	K425_625			WD40 repeat-containing protein SMU1-like protein
Mdem_scaffold_0025	523907	528025	K425_730			potassium channel subfamily K-like protein
Mdem_scaffold_0025	529789	532637	K425_729			potassium channel subfamily K-like protein
Mdem_scaffold_0025	532856	536671	K425_728			potassium channel subfamily K-like protein
Mdem_scaffold_0025	536797	542078	K425_727			conserved uncharacterized protein
Mdem_scaffold_0025	545828	547968	K425_626			RNA polymerase II subunit A C-terminal domain phosphatase SSU72-like protein
Mdem_scaffold_0025	548000	550937	K425_726			large subunit GTPase 1-like protein
Mdem_scaffold_0025	551320	558963	K425_725			conserved uncharacterized protein
Mdem_scaffold_0025	557715	562110	K425_627			ras-related protein Rab-18-like protein
Mdem_scaffold_0025	561603	565044	K425_724			protein RCC2-like protein
Mdem_scaffold_0025	567029	568381	K425_723			mpv17-like protein 2-like protein
Mdem_scaffold_0025	568908	570060	K425_722			60S ribosomal protein L37
Mdem_scaffold_0025	570073	572232	K425_720			Translation initiation factor eIF-2B subunit gamma
Mdem_scaffold_0025	572347	574284	K425_721			aspartylglucosaminidase precursor-like protein
Mdem_scaffold_0025	574021	582301	K425_719			replication protein A 70 kDa DNA-binding subunit-like protein
Mdem_scaffold_0025	579013	585320	K425_629			WD-repeat containing protein
Mdem_scaffold_0025	585655	591733	K425_718			SNAP receptor complex member-like protein
Mdem_scaffold_0025	585902	587281	K425_628			tRNA (adenine-N(1)-)-methyltransferase non-catalytic subunit TRM6-like protein
Mdem_scaffold_0025	590667	594770	K425_630			N-lysine methyltransferase SET-like protein
Mdem_scaffold_0025	595187	602798	K425_631			conserved uncharacterized protein
Mdem_scaffold_0025	603310	606199	K425_632			DEAD-box helicase Dbp80-like protein
Mdem_scaffold_0025	606188	609316	K425_717			chaperonin GroL
Mdem_scaffold_0025	609403	610753	K425_633			10 kDa heat shock protein, mitochondrial-like protein
Mdem_scaffold_0025	610741	612518	K425_716			something about silencing protein 10-like protein
Mdem_scaffold_0025	615550	617876	K425_634			mitochondrial ribosomal protein L20

Mdem_scaffold_0025	617266	618920	K425_715			triose-phosphate isomerase
Mdem_scaffold_0025	618904	620152	K425_635			INO80 complex subunit C-like protein
Mdem_scaffold_0025	620831	622895	K425_714			protein wos2-like protein
Mdem_scaffold_0025	634745	645631	K425_636			nostrin-like protein
Mdem_scaffold_0025	645632	647505	K425_712			lipase domain-containing protein
Mdem_scaffold_0025	647832	651964	K425_713			exosome component 10-like protein
Mdem_scaffold_0025	648502	653194	K425_637			protein canopy-1-like protein
Mdem_scaffold_0025	652830	656081	K425_711			GPI transamidase component PIG-T-like protein
Mdem_scaffold_0025	662191	670135	K425_710			TAR DNA-binding protein 43-like protein
Mdem_scaffold_0025	670315	675487	K425_709			NLS-binding and DNA-binding and dimerization domain-containing protein
Mdem_scaffold_0025	677098	679300	K425_708			heat shock 70 kDa-like protein
Mdem_scaffold_0025	704178	723207	K425_638			conserved uncharacterized protein
Mdem_scaffold_0025	725469	742098	K425_707			transporter, solute:sodium symporter (SSS) family
Mdem_scaffold_0025	742754	746182	K425_639			Coatomer subunit gamma-2
Mdem_scaffold_0025	746681	749002	K425_640			methionine aminopeptidase, type II
Mdem_scaffold_0025	750611	752955	K425_706			conserved uncharacterized protein
Mdem_scaffold_0025	765700	768062	K425_705			hypothetical protein
Mdem_scaffold_0025	768237	770743	K425_641			dihydrolipoyl dehydrogenase
Mdem_scaffold_0025	771575	775897	K425_642			dedicator of cytokinesis protein 3-like protein
Mdem_scaffold_0025	776901	780838	K425_704			Innexin inx3
Mdem_scaffold_0025	788374	795421	K425_643			dedicator of cytokinesis protein 3-like protein
Mdem_scaffold_0025	796272	806982	K425_703			protocadherin-like protein wing polarity protein stan-like protein
Mdem_scaffold_0025	812452	814891	K425_644			conserved uncharacterized protein
Mdem_scaffold_0025	812599	820387	K425_702			zinc transporter 9-like protein
Mdem_scaffold_0025	815141	816001	K425_645			conserved uncharacterized protein
Mdem_scaffold_0025	820904	824543	K425_701			Tudor domain-containing protein
Mdem_scaffold_0025	832142	838071	K425_646			tetraspanin-11-like protein
Mdem_scaffold_0025	840448	842318	K425_647			conserved uncharacterized protein
Mdem_scaffold_0025	842295	844205	K425_700			putative enoyl-CoA hydratase, mitochondrial-like protein
Mdem_scaffold_0025	844893	847111	K425_648			putative oxidoreductase yrbE-like protein
Mdem_scaffold_0025	847106	849961	K425_699			protein DDI1 2-like protein
Mdem_scaffold_0025	850608	853437	K425_649			N-acetylglucosamine-1-phosphotransferase subunits alpha/beta-like protein
Mdem_scaffold_0025	853672	855420	K425_698			conserved uncharacterized protein
Mdem_scaffold_0025	856519	860092	K425_650			chromatin-remodeling complex ATPase chain Iswi-like protein
Mdem_scaffold_0025	863009	864761	K425_697			CUE domain-containing protein
Mdem_scaffold_0025	868043	869132	K425_651			conserved uncharacterized protein
Mdem_scaffold_0025	870017	872298	K425_652			hemolymph juvenile hormone binding protein (JHBP) domain-containing protein
Mdem_scaffold_0025	875199	893722	K425_696			amyloid beta A4 precursor protein-binding family B member 2-like isoform 2
Mdem_scaffold_0025	905053	906600	K425_653			cytochrome P450 domain-containing protein
Mdem_scaffold_0025	918198	924532	K425_695			ubiquitin thioesterase trabid-like protein
Mdem_scaffold_0025	921066	923455	K425_655			hypothetical protein
Mdem_scaffold_0025	924544	927304	K425_654			DNA-directed RNA polymerase III subunit RPC3-like protein
Mdem_scaffold_0025	927895	932831	K425_656			protein shuttle craft-like protein
Mdem_scaffold_0025	936252	939997	K425_694			conserved uncharacterized protein
Mdem_scaffold_0025	941287	945328	K425_693			Ribonuclease P protein subunit p20
Mdem_scaffold_0025	942682	945176	K425_657			Cleavage stimulation factor 50 kDa subunit
Mdem_scaffold_0025	945643	948564	K425_659			conserved uncharacterized protein
Mdem_scaffold_0025	947143	951044	K425_692			DNA-directed RNA polymerase II subunit RPB9
Mdem_scaffold_0025	953427	955073	K425_658			bric-a-brac-like protein
Mdem_scaffold_0025	962212	965357	K425_660			ankyrin-repeat containing protein
Mdem_scaffold_0025	965871	968028	K425_661			NADH dehydrogenase (quinone), D subunit
Mdem_scaffold_0025	985443	993999	K425_662			scavenger receptor class B member-like protein
Mdem_scaffold_0025	987291	990647	K425_691			putative reverse transcriptase
Mdem_scaffold_0025	995084	997247	K425_690			scavenger receptor class B member-like protein
Mdem_scaffold_0025	999204	1002763	K425_663			ankyrin-repeat containing protein
Mdem_scaffold_0025	1004225	1008076	K425_664			DNA-binding protein Ets97D-like protein
Mdem_scaffold_0025	1039265	1051290	K425_689			Ubiquitin carboxyl-terminal hydrolase 2-like protein
Mdem_scaffold_0025	1052447	1057547	K425_688			protein RRP5-like protein
Mdem_scaffold_0025	1058387	1061616	K425_665			hypothetical protein
Mdem_scaffold_0025	1087446	1087518	K425_601			tRNA-Arg
Mdem_scaffold_0025	1088392	1095373	K425_666			calpain-D-like protein
Mdem_scaffold_0025	1092653	1093186	K425_687			hypothetical protein
Mdem_scaffold_0025	1100780	1103368	K425_667			ring box protein
Mdem_scaffold_0025	1106591	1110631	K425_686			otopetrin-3-like protein
Mdem_scaffold_0025	1145659	1153635	K425_685			conserved uncharacterized protein
Mdem_scaffold_0025	1207355	1208673	K425_684			ankyrin-repeat containing protein
Mdem_scaffold_0025	1225438	1228427	K425_683			EB module-containing protein
Mdem_scaffold_0025	1277456	1289141	K425_668			zeta-sarcoglycan-like protein
Mdem_scaffold_0025	1302954	1304844	K425_682			prostaglandin reductase 1-like protein
Mdem_scaffold_0025	1305694	1312160	K425_681			rapamycin-insensitive companion of mTOR-like protein
**Mdem_scaffold_0025**	**1314117**	**1315067**	**K425_680**	**ptp-U1**		**protein tyrosine phosphatase**
**Mdem_scaffold_0025**	**1316816**	**1317748**	**K425_679**	**ptp-U2**		**protein tyrosine phosphatase**
Mdem_scaffold_0025	1320563	1324187	K425_669			cell cycle checkpoint protein RAD1-like protein
Mdem_scaffold_0025	1325074	1330227	K425_678			SH2B adapter protein 1-like protein
Mdem_scaffold_0025	1329169	1331636	K425_671			conserved uncharacterized protein
Mdem_scaffold_0025	1331840	1340593	K425_670			Phosphatidylinositol 4-kinase alpha
Mdem_scaffold_0025	1340709	1343558	K425_676			NTF2-related export-like protein
Mdem_scaffold_0025	1343191	1345423	K425_672			asparagine-linked glycosylation-like protein
Mdem_scaffold_0025	1345493	1347532	K425_675			RNA 3'-phosphate cyclase

Mdem_scaffold_0025	1347429	1348652	K425_673			vacuolar-sorting protein SNF8
Mdem_scaffold_0025	1348562	1351220	K425_677			sodium channel and clathrin linker 1-like protein
Mdem_scaffold_0025	1391028	1399435	K425_674			synaptotagmin-C-like protein
Mdem_scaffold_0040	4557	6287	K425_1313			Akt1-like protein, partial
Mdem_scaffold_0040	9790	11232	K425_1312			mitochondrial ribosomal protein S28
Mdem_scaffold_0040	11491	12686	K425_1311			cytochrome b5 type B-like protein
Mdem_scaffold_0040	19261	41523	K425_1225			MORN repeat-containing protein
Mdem_scaffold_0040	55713	79215	K425_1226			Autophagy-related protein 11 domain-containing protein
Mdem_scaffold_0040	83757	87973	K425_1310			TRAF-interacting-like protein
Mdem_scaffold_0040	88202	94729	K425_1227			caspase-8-like protein
Mdem_scaffold_0040	96094	100604	K425_1309			nicalin-1-like protein
Mdem_scaffold_0040	100025	102372	K425_1228			activator of basal transcription 1-like protein
Mdem_scaffold_0040	102847	106508	K425_1308			trimeric intracellular cation channel-like protein
Mdem_scaffold_0040	113351	120787	K425_1229		similar to venom protein 2 from Microctonus hyperodae	conserved uncharacterized protein family 3
Mdem_scaffold_0040	124391	134511	K425_1230		similar to venom protein 2 from Microctonus hyperodae	conserved uncharacterized protein family 3
Mdem_scaffold_0040	137920	144554	K425_1231			conserved uncharacterized protein family 3
Mdem_scaffold_0040	148198	151849	K425_1232			S-methyl-5'-thioadenosine phosphorylase-like protein
Mdem_scaffold_0040	152430	159752	K425_1307			S-methyl-5'-thioadenosine phosphorylase-like protein
Mdem_scaffold_0040	168629	169587	K425_1306			S-methyl-5'-thioadenosine phosphorylase-like protein
Mdem_scaffold_0040	172288	174731	K425_1233			mannose-1-phosphate guanyltransferase alpha-like protein
Mdem_scaffold_0040	178084	190618	K425_1234			XK-related protein 7-like protein
Mdem_scaffold_0040	253175	256782	K425_1235			protein msta-like protein
Mdem_scaffold_0040	258087	262087	K425_1236			Zinc-finger C2H2 domain-containing protein
Mdem_scaffold_0040	263985	266962	K425_1237			Synaptic glycoprotein SC2
Mdem_scaffold_0040	266960	270370	K425_1305			WD repeat-containing protein 65-like protein
Mdem_scaffold_0040	269760	283894	K425_1238			WD repeat-containing protein
Mdem_scaffold_0040	284771	288908	K425_1304			TATA binding protein
Mdem_scaffold_0040	288927	291487	K425_1303			mTERF domain-containing protein 1, mitochondrial
Mdem_scaffold_0040	293359	298484	K425_1239			reactive oxygen species modulator 1-like protein
Mdem_scaffold_0040	296633	302764	K425_1302			intermediate compartment protein 3-like protein
Mdem_scaffold_0040	303780	305728	K425_1240			tRNA selenocysteine 1-associated protein 1-like protein
Mdem_scaffold_0040	306387	307600	K425_1301			mitochondrial ribosomal protein L18
Mdem_scaffold_0040	308219	311109	K425_1241			TRAF-interacting-like protein
Mdem_scaffold_0040	312402	314767	K425_1300			GDP-mannose 4,6-dehydratase
Mdem_scaffold_0040	317771	324812	K425_1242			conserved uncharacterized protein
Mdem_scaffold_0040	355902	357762	K425_1244			rho GTPase-activating protein 1-like protein
Mdem_scaffold_0040	360418	364942	K425_1245			rho GTPase-activating protein 1-like protein
Mdem_scaffold_0040	365287	368617	K425_1299			Ras-related protein Rab-21
Mdem_scaffold_0040	369276	374558	K425_1246			conserved uncharacterized protein
Mdem_scaffold_0040	381961	383715	K425_1247			rhythmically expressed 2-like protein
Mdem_scaffold_0040	384894	398572	K425_1248			laminin subunit gamma-1-like protein
Mdem_scaffold_0040	400965	407136	K425_1298			syntaxin-like protein
Mdem_scaffold_0040	410753	420739	K425_1249			myelin expression factor 2-like protein
Mdem_scaffold_0040	423806	441418	K425_1297			Protein fem-1-like protein CG6966
Mdem_scaffold_0040	447221	485453	K425_1250			protein groucho-like protein
Mdem_scaffold_0040	493737	505855	K425_1296			Integrator complex subunit 3-like protein
Mdem_scaffold_0040	508194	509386	K425_1251			Phosphatidylinositol-specific phospholipase C, X domain-like protein
Mdem_scaffold_0040	508241	519580	K425_1295			phosphatidylinositol 3-kinase catalytic subunit type 3-like protein
Mdem_scaffold_0040	522045	529980	K425_1252			dnaJ subfamily C member 10-like protein
Mdem_scaffold_0040	530566	535043	K425_1294			putative achaete scute target 1-like protein
Mdem_scaffold_0040	538450	546975	K425_1293			acid phosphatase-like protein
Mdem_scaffold_0040	584755	588057	K425_1253			serine--tRNA ligase
Mdem_scaffold_0040	588723	593641	K425_1272			disintegrin and metalloprotease-like protein
Mdem_scaffold_0040	593817	599502	K425_1273			disintegrin and metalloprotease-like protein
Mdem_scaffold_0040	602292	609398	K425_1274			disintegrin and metalloprotease-like protein
Mdem_scaffold_0040	609399	619668	K425_1275			disintegrin and metalloprotease-like protein
Mdem_scaffold_0040	619809	620651	K425_1254			disintegrin and metalloprotease-like protein
Mdem_scaffold_0040	623123	626526	K425_1255			disintegrin and metalloprotease-like protein
Mdem_scaffold_0040	635173	636665	K425_1292			Phosphatidylinositol-specific phospholipase C, X domain-containing protein
Mdem_scaffold_0040	642281	649253	K425_1256			conserved uncharacterized protein family 3
Mdem_scaffold_0040	655554	660345	K425_1257			conserved uncharacterized protein family 3
Mdem_scaffold_0040	661839	666698	K425_1258			Phosphatidylinositol-specific phospholipase C, X domain-containing protein
Mdem_scaffold_0040	668684	670827	K425_1291			conserved uncharacterized protein family 3
Mdem_scaffold_0040	674513	675505	K425_1290			Phosphatidylinositol-specific phospholipase C, X domain-like protein
Mdem_scaffold_0040	676584	677582	K425_1259			Phosphatidylinositol-specific phospholipase C, X domain-like protein
Mdem_scaffold_0040	683158	684174	K425_1289			Phosphatidylinositol-specific phospholipase C, X domain-like protein
Mdem_scaffold_0040	686206	687675	K425_1288			Phosphatidylinositol-specific phospholipase C, X domain-like protein
Mdem_scaffold_0040	687785	690642	K425_1287			DnaJ-like protein subfamily C member 17-like protein
**Mdem_scaffold_0040**	**693842**	**695701**	**K425_1260**	**pif-5-2**	**synonym odv-e56**	**putative envelope protein ODV-E56-2**
**Mdem_scaffold_0040**	**696993**	**698622**	**K425_1261**	**pif-5-3**	**synonym odv-e56**	**putative envelope protein ODV-E56-3**
Mdem_scaffold_0040	746474	752819	K425_1262			conserved uncharacterized protein
Mdem_scaffold_0040	754153	758298	K425_1286			BTB/POZ domain-containing protein 2-like protein
Mdem_scaffold_0040	758441	765090	K425_1263			rab3 GTPase-activating protein non-catalytic subunit-like protein
Mdem_scaffold_0040	765800	768221	K425_1284			acylphosphatase-2-like protein
Mdem_scaffold_0040	769459	779216	K425_1285			conserved uncharacterized protein
Mdem_scaffold_0040	784487	789236	K425_1283			PCTP-like protein
Mdem_scaffold_0040	790391	799294	K425_1264			phosphoglucomutase-2-like protein
Mdem_scaffold_0040	798139	804196	K425_1282			conserved uncharacterized protein
Mdem_scaffold_0040	806534	813504	K425_1265			kinesin-like protein KIF11

Mdem_scaffold_0040	813753	820142	K425_1281			trypsin-like protein
Mdem_scaffold_0040	820784	822293	K425_1266			conserved uncharacterized protein
Mdem_scaffold_0040	821581	827263	K425_1280			Annexin-B9
Mdem_scaffold_0040	828463	831272	K425_1267			Transmembrane protein 70-like protein
Mdem_scaffold_0040	831257	846014	K425_1279			ankyrin-repeat domain-containing protein
Mdem_scaffold_0040	847511	856069	K425_1278			neuroligin-4, Y-linked-like protein
Mdem_scaffold_0040	858563	871165	K425_1268			neurogenic protein big brain-like protein
Mdem_scaffold_0040	869457	874333	K425_1277			transmembrane protein 50A-like protein
Mdem_scaffold_0040	875224	879840	K425_1269			calcineurin subunit B type 2-like protein
Mdem_scaffold_0040	882343	886087	K425_1270			tissue inhibitor of metalloproteases domain-containing protein
Mdem_scaffold_0040	888510	896727	K425_1276			5' nucleotidase, putative
Mdem_scaffold_0040	898591	899459	K425_1271			tissue inhibitor of metalloproteases domain-containing protein
Mdem_scaffold_0067	72	1477	K425_1559			histone H1-like protein
**Mdem_scaffold_0067**	**5376**	**7229**	**K425_1560**	**odv-e66-13**		**putative envelope protein ODV-E66-13**
Mdem_scaffold_0067	10257	12926	K425_1561			conserved uncharacterized protein
Mdem_scaffold_0067	14357	35646	K425_1562			piopio-like protein
Mdem_scaffold_0067	40186	46452	K425_1592			conserved uncharacterized protein
Mdem_scaffold_0067	59003	61474	K425_1563			conserved uncharacterized protein
Mdem_scaffold_0067	68883	85404	K425_1564			P-type ATPase of unknown pump specificity (type V)
Mdem_scaffold_0067	85891	86488	K425_1591			histone H2-like protein
Mdem_scaffold_0067	86284	87180	K425_1565			histone H2-like protein
Mdem_scaffold_0067	91506	92091	K425_1566			histone H3-like protein
Mdem_scaffold_0067	94424	94495	K425_1558			tRNA-Glu
Mdem_scaffold_0067	96731	101006	K425_1567			Chondroitin N-acetylgalactosaminyltransferase domain-containing protein
Mdem_scaffold_0067	100358	104502	K425_1590			ell-associated factor Eaf-like protein
Mdem_scaffold_0067	107093	107254	K425_1589			hypothetical protein
Mdem_scaffold_0067	108986	115197	K425_1588			conserved uncharacterized protein
Mdem_scaffold_0067	118310	126705	K425_1587			neprilysin-like protein
Mdem_scaffold_0067	128187	137461	K425_1586			conserved uncharacterized protein
Mdem_scaffold_0067	151334	152787	K425_1568			conserved uncharacterized protein
Mdem_scaffold_0067	158733	160515	K425_1585			conserved uncharacterized protein
Mdem_scaffold_0067	166485	170430	K425_1584			conserved uncharacterized protein
Mdem_scaffold_0067	172220	182211	K425_1569			hypothetical protein
Mdem_scaffold_0067	183817	193188	K425_1570			convoluted-like protein
Mdem_scaffold_0067	203210	205940	K425_1571			hypothetical protein
Mdem_scaffold_0067	208382	209719	K425_1572			hypothetical protein
Mdem_scaffold_0067	219492	225554	K425_1573			quasimodo-like protein
Mdem_scaffold_0067	224728	228630	K425_1583			methylcrotonoyl-CoA carboxylase beta chain, mitochondrial-like protein
Mdem_scaffold_0067	229463	234491	K425_1574			fanconi-associated nuclease 1-like protein
Mdem_scaffold_0067	234618	240916	K425_1575			lysophospholipid acyltransferase 5-like protein
Mdem_scaffold_0067	242526	243853	K425_1581			tweedle motif cuticular-like protein
Mdem_scaffold_0067	250734	255499	K425_1582			tweedle motif cuticular-like protein
Mdem_scaffold_0067	289057	291108	K425_1577			hypothetical protein
Mdem_scaffold_0067	325970	332014	K425_1578			hypothetical protein
Mdem_scaffold_0067	338353	355530	K425_1580			potassium voltage-gated channel subfamily-like protein
Mdem_scaffold_0067	362467	363717	K425_1579			conserved uncharacterized protein
Mdem_scaffold_0116	71602	73213	K425_180		similar to GfV-B16-ORF1-like protein	conserved uncharacterized protein family 6
Mdem_scaffold_0116	177044	178186	K425_142		similar to GfV-B16-ORF1-like protein	conserved uncharacterized protein family 6
Mdem_scaffold_0116	183517	196158	K425_179			Origin recognition complex (ORC) subunit 3 domain-containing protein
**Mdem_scaffold_0116**	**193944**	**194807**	**K425_143**	**pif-4**	**present in Microplitis demolitor virus particles**	**per os infectivity factor pif-4 (19kDa)**
Mdem_scaffold_0116	195537	228610	K425_144			Fasciclin domain-containing protein
Mdem_scaffold_0116	215033	216174	K425_178			skpF-like protein
Mdem_scaffold_0116	228908	233376	K425_145			skpB-like protein
Mdem_scaffold_0116	234019	236247	K425_146			skpA-like protein
Mdem_scaffold_0116	238044	240484	K425_147			Dolichol-phosphate mannosyltransferase subunit 3 (DPM3) domain-containing protein
Mdem_scaffold_0116	239053	241981	K425_177			diphthamide biosynthesis protein 2
Mdem_scaffold_0116	247684	250086	K425_176			Ras-related protein Rab-8A-like protein
Mdem_scaffold_0116	253075	256527	K425_175			conserved uncharacterized protein
**Mdem_scaffold_0116**	**259784**	**260881**	**K425_148**	**odv-e66-4**		**putative envelope protein ODV-E66-4, partial**
Mdem_scaffold_0116	262190	264795	K425_149			fas apoptotic inhibitory molecule 2-like protein
Mdem_scaffold_0116	267228	271795	K425_150			3-hydroxyisobutyryl-CoA hydrolase, mitochondrial-like protein
Mdem_scaffold_0116	274946	279407	K425_174			GTP-binding protein SAR1b-like protein
Mdem_scaffold_0116	284169	298554	K425_151			vacuolar protein sorting-associated protein 13C-like protein
Mdem_scaffold_0116	299684	301688	K425_173			carbonic anhydrase-like protein
Mdem_scaffold_0116	304097	318764	K425_152			metal transporter CNNM2-like protein
Mdem_scaffold_0116	326533	332342	K425_153			neuropeptide FF receptor 2-like protein
Mdem_scaffold_0116	333257	336188	K425_172			conserved uncharacterized protein
Mdem_scaffold_0116	334542	340199	K425_154			protein farnesyltransferase subunit beta-like protein
Mdem_scaffold_0116	341820	343398	K425_155			ATP synthase subunit H domain-containing protein
Mdem_scaffold_0116	346684	350277	K425_171			Serendipity locus alpha protein (SRY-A) domain-containing protein
Mdem_scaffold_0116	354631	370850	K425_156			conserved uncharacterized protein
Mdem_scaffold_0116	358192	359199	K425_170			conserved uncharacterized protein
Mdem_scaffold_0116	387876	395755	K425_169			chaoptin-like protein
Mdem_scaffold_0116	418436	420157	K425_158			Maltase 1-like protein
Mdem_scaffold_0116	423897	425829	K425_157			Maltase 1-like protein
Mdem_scaffold_0116	426311	428275	K425_160			Maltase 1-like protein
Mdem_scaffold_0116	428813	430674	K425_159			Maltase 1-like protein
Mdem_scaffold_0116	431356	433062	K425_161			Maltase 1-like protein
Mdem_scaffold_0116	435118	437033	K425_162			Maltase 1-like protein

Mdem_scaffold_0116	438303	438389	K425_141			tRNA-Ser
Mdem_scaffold_0116	440520	440585	K425_168			hypothetical protein
Mdem_scaffold_0116	460056	461749	K425_163			hypothetical protein
Mdem_scaffold_0116	594965	595039	K425_140			tRNA-Thr
Mdem_scaffold_0116	693890	701966	K425_164			chaoptin-like protein
Mdem_scaffold_0116	720116	721074	K425_167			ion-transport peptide precursor-like protein
Mdem_scaffold_0116	739737	744872	K425_165			ras-related protein Rab-27A-like protein
Mdem_scaffold_0116	751128	778997	K425_166			disintegrin and metalloproteinase domain-containing protein
Mdem_scaffold_0130	11508	15182	K425_191			LIM domain-containing protein
Mdem_scaffold_0130	29438	34272	K425_207			conserved uncharacterized protein
Mdem_scaffold_0130	40078	43161	K425_205			conserved uncharacterized protein family 2
Mdem_scaffold_0130	44333	52863	K425_206			conserved uncharacterized protein family 2
**Mdem_scaffold_0130**	**54349**	**55823**	**K425_204**	**pif-2**	**present in Microplitis demolitor virus particles**	**per os infectivity factor pif-2**
**Mdem_scaffold_0130**	**57973**	**58317**	**K425_192**		**similar to pif-1**	**hypothetical protein**
**Mdem_scaffold_0130**	**58164**	**59927**	**K425_193**	**pif-1**	**present in Microplitis demolitor virus particles**	**per os infectivity factor pif-1**
Mdem_scaffold_0130	78416	97999	K425_194			7 transmembrane receptor (rhodopsin family) domain-containing protein
Mdem_scaffold_0130	99391	106116	K425_203			MFS sugar transporter, spinster-like protein
Mdem_scaffold_0130	123285	129513	K425_195			Ion channel regulatory protein UNC-93 domain-containing protein
Mdem_scaffold_0130	132378	139352	K425_202			Proline dehydrogenase domain-containing protein
Mdem_scaffold_0130	141882	147372	K425_196			elongator complex protein 4-like protein
Mdem_scaffold_0130	152188	163763	K425_197			chaperone protein DnaK
Mdem_scaffold_0130	164217	170820	K425_198			ethanolamine kinase 1-like protein
Mdem_scaffold_0130	172538	180164	K425_199			lobe-like protein
Mdem_scaffold_0130	180657	184751	K425_208			tetraspanin domain-containing protein
Mdem_scaffold_0130	240037	249232	K425_200			bric-a-brac-like protein
Mdem_scaffold_0130	259598	267609	K425_201			nanos-like protein
Mdem_scaffold_0157	3446	3697	K425_416			hypothetical protein
Mdem_scaffold_0157	4312	4797	K425_417			hypothetical protein
Mdem_scaffold_0157	10395	11585	K425_418			7tm Chemosensory receptor domain-containing protein
Mdem_scaffold_0157	15777	17842	K425_419			7tm Chemosensory receptor domain-containing protein
Mdem_scaffold_0157	20931	25147	K425_420			7tm Chemosensory receptor domain-containing protein
Mdem_scaffold_0157	38299	41566	K425_434			hypothetical protein
Mdem_scaffold_0157	60220	65270	K425_421			hypothetical protein
Mdem_scaffold_0157	67218	68082	K425_433			hypothetical protein
Mdem_scaffold_0157	69293	70329	K425_422		similar to GIP_L2_0080	conserved uncharacterized protein
Mdem_scaffold_0157	73114	75307	K425_423			7tm Chemosensory receptor domain-containing protein
Mdem_scaffold_0157	76503	77398	K425_432			histone H4 replacement-like protein
Mdem_scaffold_0157	78313	79761	K425_424			Partial alpha/beta-hydrolase lipase region-containing protein
Mdem_scaffold_0157	80170	84382	K425_425			Partial alpha/beta-hydrolase lipase region-like protein
Mdem_scaffold_0157	87157	92143	K425_431		similar to venom protein 2 from Microctonus hyperodae	conserved uncharacterized protein family 3
**Mdem_scaffold_0157**	**105552**	**106552**	**K425_430**		**similar to hypothetical protein ABX44706 from Penaeus monodon nudivirus and HzNVorf93**	**conserved uncharacterized protein**
Mdem_scaffold_0157	110394	121443	K425_426			Nuclear pore complex protein Nup205
Mdem_scaffold_0157	123495	124412	K425_429			Phosphatidylinositol-specific phospholipase C, X domain-containing protein
Mdem_scaffold_0157	125221	126069	K425_428			Phosphatidylinositol N-acetylglucosaminyltransferase subunit C
Mdem_scaffold_0157	129406	132398	K425_427			transcription initiation factor TFIID subunit 6-like protein
Mdem_scaffold_0159	6237	10641	K425_474			DNA topoisomerase 2-binding protein 1-like protein
Mdem_scaffold_0159	11833	15626	K425_473	gidA		tRNA uridine 5-carboxymethylaminomethyl modification enzyme GidA
Mdem_scaffold_0159	186762	188336	K425_435			hypothetical protein
Mdem_scaffold_0159	239920	242902	K425_437			protein-tyrosine sulfotransferase-like protein
Mdem_scaffold_0159	247905	256086	K425_436			DnaJ domain-containing protein
Mdem_scaffold_0159	283560	291456	K425_472	purN		phosphoribosylglycinamide formyltransferase
Mdem_scaffold_0159	294254	299302	K425_471			protein tyrosine kinase domain-containing protein
Mdem_scaffold_0159	301800	302681	K425_438			hypothetical protein
Mdem_scaffold_0159	306036	308385	K425_439			conserved hypothetical protein
**Mdem_scaffold_0159**	309992	311462	**K425_440**	**HzNVorf118-like (PmV-like)**	**similar to HzNVorf118 and PmV hypothetical protein**	**Bracovirus particle protein HzNVorf118-like (PmV-like)**
**Mdem_scaffold_0159**	315174	316855	**K425_470**	**pif-3**		**per os infectivity factor 3**
**Mdem_scaffold_0159**	316956	318148	**K425_441**	**vp39**	**similar to HzNVorf89**	**Major bracovirus capsid protein vp39**
**Mdem_scaffold_0159**	319248	319955	**K425_469**	**27b-like-1**	**similar to Cotesia congregata 27b product of Cc50C22.7**	**Bracovirus particle protein 27b-like-1**
**Mdem_scaffold_0159**	320040	321226	**K425_468**	**Cc50C22.6-like-1**	**similar to Cotesia congregata Cc50C22.6**	**conserved hypothetical protein family 4**
**Mdem_scaffold_0159**	**325975**	**328544**	**K425_442**	**Cc50C22.5-like**	**similar to Cotesia congregata Cc50C22.5**	**Bracovirus particle protein**
**Mdem_scaffold_0159**	**329696**	**331138**	**K425_443**	**HzNVorf9-like 1**		**Bracovirus particle protein HzNVorf9-like 1**
**Mdem_scaffold_0159**	**331947**	**332375**	**K425_444**	**Cc50C22.3-like**	**similar to Cotesia congregata Cc50C22.3**	**conserved hypothetical protein**
**Mdem_scaffold_0159**	**332331**	**333003**	**K425_446**	**HzNVorf94-like-1**		**hypothetical protein**
**Mdem_scaffold_0159**	**334139**	**335041**	**K425_463**	**38K-1**	**similar to HzNVorf108**	**Bracovirus particle protein 38K-2**
**Mdem_scaffold_0159**	**336616**	**337482**	**K425_462**	**35a-2**	**similar to 35a protein from Chelonus inanitus**	**conserved uncharacterized protein 35a-like protein-3**
**Mdem_scaffold_0159**	**339560**	**340144**	**K425_461**			**hypothetical protein**
Mdem_scaffold_0159	342879	348823	K425_449			exportin domain-containing protein
Mdem_scaffold_0159	347604	354810	K425_447			aldehyde dehydrogenase domain-containing protein
Mdem_scaffold_0159	353604	354617	K425_460			methyltransferase domain-containing protein
Mdem_scaffold_0159	354449	361379	K425_448			lissencephaly-1-like protein

**Mdem_scaffold_0159**	**368561**	**369415**	**K425_458**	**HzNVorf106-like**		**Bracovirus particle protein HzNVorf106-like**
**Mdem_scaffold_0159**	**370429**	**371388**	**K425_457**	**HzNVorf9-like-2**		**Bracovirus particle protein HzNVorf9-like 2**
**Mdem_scaffold_0159**	**371440**	**373231**	**K425_459**			**hypothetical protein**
**Mdem_scaffold_0159**	**372569**	**373186**	**K425_450**			**hypothetical protein**
**Mdem_scaffold_0159**	**374179**	**375393**	**K425_456**			**hypothetical protein**
**Mdem_scaffold_0159**	**378378**	**379147**	**K425_451**	**Cc50C22.6-like-2**	**similar to Cotesia congregata Cc50C22.6**	**conserved hypothetical protein family 4**
**Mdem_scaffold_0159**	**379769**	**380755**	**K425_452**	**int-1**	**similar to HzNVorf144**	**viral integrase, bracovirus particle protein**
**Mdem_scaffold_0159**	**381633**	**382400**	**K425_453**	**35a-4**	**similar to 35a protein from Chelonus inanitus**	**conserved uncharacterized protein 35a-like protein-4**
Mdem_scaffold_0159	391816	393460	K425_455			kelch domain-containing protein
Mdem_scaffold_0159	397495	399201	K425_454			a-kinase anchor protein 10, mitochondrial-like protein
Mdem_scaffold_0240	120184	123924	K425_600			lipase domain-containing protein
Mdem_scaffold_0240	129142	130005	K425_561			Phosphatidylinositol-specific phospholipase C, X domain-like protein
Mdem_scaffold_0240	146864	147733	K425_563			Phosphatidylinositol-specific phospholipase C, X domain-like protein
Mdem_scaffold_0240	150440	151411	K425_562			Phosphatidylinositol-specific phospholipase C, X domain-like protein
Mdem_scaffold_0240	214596	227908	K425_599			spalt major-like protein
Mdem_scaffold_0240	457732	459619	K425_598			hypothetical protein
Mdem_scaffold_0240	471740	475632	K425_597			hypothetical protein
Mdem_scaffold_0240	475658	478611	K425_596			hypothetical protein
Mdem_scaffold_0240	482533	484998	K425_595			hypothetical protein
Mdem_scaffold_0240	555943	560152	K425_564			nose resistant to fluoxetine protein 6-like protein
Mdem_scaffold_0240	621931	628123	K425_565			hypothetical protein
**Mdem_scaffold_0240**	**658585**	**661612**	**K425_594**	**35a-5**		**conserved uncharacterized protein 35a-like protein-5**
Mdem_scaffold_0240	667806	668789	K425_593			conserved uncharacterized protein
Mdem_scaffold_0240	672496	685219	K425_592			CDK5 and ABL1 enzyme substrate 1-like protein
Mdem_scaffold_0240	688321	695807	K425_566			Gamma interferon inducible lysosomal thiol reductase (GILT) domain-containing protein
Mdem_scaffold_0240	695700	699295	K425_591			splicing factor 45-like protein
Mdem_scaffold_0240	719367	725966	K425_567			naked cuticle-like protein
Mdem_scaffold_0240	738449	744378	K425_590			conserved uncharacterized protein
Mdem_scaffold_0240	748623	749504	K425_568			Mediator of RNA polymerase II transcription subunit 29-like protein
Mdem_scaffold_0240	753136	764659	K425_569			SRF-type transcription factor (DNA-binding and dimerization domain)
Mdem_scaffold_0240	778146	787946	K425_570			abnormal spindle-like protein
Mdem_scaffold_0240	788705	801850	K425_589			protein Mo25-like protein
Mdem_scaffold_0240	807652	814359	K425_571			conserved uncharacterized protein
Mdem_scaffold_0240	813986	818915	K425_588			pleiotropic regulator 1-like protein
Mdem_scaffold_0240	825275	829323	K425_587			protein msta, isoform A-like protein
Mdem_scaffold_0240	837900	841330	K425_586			SDA1 domain-containing protein
Mdem_scaffold_0240	842022	843655	K425_585			origin recognition complex subunit 5-like protein
Mdem_scaffold_0240	844986	849878	K425_572			conserved uncharacterized protein
Mdem_scaffold_0240	847018	855347	K425_584			amidophosphoribosyltransferase
Mdem_scaffold_0240	857000	861524	K425_573			SAICAR synthetase domain-containing protein
Mdem_scaffold_0240	865191	869188	K425_583			Metallopeptidase family M24 protein
Mdem_scaffold_0240	871082	873801	K425_574			conserved uncharacterized protein
Mdem_scaffold_0240	876017	882688	K425_582			coiled-coil domain-containing protein 151-like protein
Mdem_scaffold_0240	934477	937307	K425_581			hypothetical protein
Mdem_scaffold_0240	958673	963684	K425_575			notch-like protein
Mdem_scaffold_0240	965147	966468	K425_580			hypothetical protein
Mdem_scaffold_0240	994555	1017195	K425_576			notch-like protein
Mdem_scaffold_0240	1050883	1068090	K425_577			notch-like protein
Mdem_scaffold_0240	1076655	1087878	K425_579			polycomb-like protein
Mdem_scaffold_0240	1091029	1092219	K425_578			leucine-rich repeat-containing protein
Mdem_scaffold_0275	750	20646	K425_915			ligand-gated ion channel pHCl-like protein
Mdem_scaffold_0275	37937	39867	K425_751			trypsin-like protein
Mdem_scaffold_0275	41878	45439	K425_752			trypsin-like protein
Mdem_scaffold_0275	52770	54948	K425_753			trypsin-like protein
Mdem_scaffold_0275	54822	59503	K425_914			Flavin-containing monooxygenase-like protein
Mdem_scaffold_0275	61852	72695	K425_913			Sterile20-like protein
Mdem_scaffold_0275	76898	84546	K425_912			fatty-acid amide hydrolase 2-like protein
Mdem_scaffold_0275	85141	86711	K425_754			mimitin, mitochondrial-like protein
Mdem_scaffold_0275	86698	97434	K425_911			poly(U)-binding-splicing factor half pint-like protein
Mdem_scaffold_0275	98436	100673	K425_755			SET domain-containing protein
Mdem_scaffold_0275	213994	228851	K425_756			Immunoglobulin domain-containing protein
**Mdem_scaffold_0275**	**228828**	**230147**	**K425_910**	**pif-5-1**	**synonym odv-e56**	**putative envelope protein ODV-E56-1**
Mdem_scaffold_0275	237692	238423	K425_909			atonal-like protein
Mdem_scaffold_0275	245055	246443	K425_908			DNL-type zinc finger-like protein
Mdem_scaffold_0275	247077	249126	K425_757			HAD hydrolase, family IIID
Mdem_scaffold_0275	249966	250037	K425_750			tRNA-Asp
Mdem_scaffold_0275	250759	256399	K425_758			putative mitochondrial inner membrane-like protein
Mdem_scaffold_0275	256376	259330	K425_907			Fumarylacetoacetate hydrolase domain-containing protein
Mdem_scaffold_0275	260171	266542	K425_759			E3 ubiquitin-protein ligase UHRF1-like protein
Mdem_scaffold_0275	263951	266624	K425_906			NADH:ubiquinone reductase 23kD subunit precursor
Mdem_scaffold_0275	267232	270177	K425_905			conserved uncharacterized protein
Mdem_scaffold_0275	273302	285130	K425_760			conserved uncharacterized protein
Mdem_scaffold_0275	289178	291452	K425_904			aurora-like protein
Mdem_scaffold_0275	291600	300643	K425_761			DEAD/DEAH box helicase domain-containing protein
Mdem_scaffold_0275	298205	302932	K425_903			putative methyltransferase
Mdem_scaffold_0275	300818	307295	K425_762			Transmembrane-like protein
Mdem_scaffold_0275	307513	311022	K425_902			Beta-1,4-galactosyltransferase-like protein
Mdem_scaffold_0275	311472	318777	K425_763			dynactin subunit 1-like protein
Mdem_scaffold_0275	321765	325124	K425_901			Casein kinase I isoform alpha-like protein
Mdem_scaffold_0275	325919	332036	K425_764			egalitarian-like protein

Mdem_scaffold_0275	332726	335485	K425_765			conserved uncharacterized protein
Mdem_scaffold_0275	335530	346673	K425_900			huntingtin-like protein
Mdem_scaffold_0275	345726	349212	K425_766			thioredoxin peroxidase-like protein
Mdem_scaffold_0275	349765	352531	K425_767			conserved uncharacterized protein
Mdem_scaffold_0275	352627	360267	K425_899			Phosphatidylinositide phosphatase SAC2-like protein
Mdem_scaffold_0275	360680	364218	K425_768			docking-like protein
Mdem_scaffold_0275	370440	378938	K425_769			hypothetical protein
Mdem_scaffold_0275	389321	393621	K425_770			anaphase-promoting complex subunit 7-like protein
Mdem_scaffold_0275	396541	398270	K425_771			rabphilin-like protein
Mdem_scaffold_0275	398729	404394	K425_898			conserved uncharacterized protein
Mdem_scaffold_0275	404589	406040	K425_772			conserved uncharacterized protein
Mdem_scaffold_0275	406547	409490	K425_897			conserved uncharacterized protein
Mdem_scaffold_0275	409716	412154	K425_773			Thioredoxin domain-containing protein
Mdem_scaffold_0275	412618	414177	K425_774			vacuolar protein sorting-associated protein 37B-like protein
Mdem_scaffold_0275	414396	415413	K425_896			DnaJ domain-containing protein
Mdem_scaffold_0275	415898	418381	K425_776			Copper chaperone for superoxide dismutase-like protein
Mdem_scaffold_0275	419021	421682	K425_775			nitrilase 1-like protein
Mdem_scaffold_0275	421570	422459	K425_895			small ribonucleoprotein particle protein SmD3
Mdem_scaffold_0275	422144	424479	K425_777			conserved uncharacterized protein
Mdem_scaffold_0275	424344	425754	K425_894			Histone RNA hairpin-binding protein RNA-binding domain-containing protein
Mdem_scaffold_0275	426514	430608	K425_778			AN1-type zinc finger protein 1-like protein
Mdem_scaffold_0275	430618	437842	K425_893			Endonuclease/Exonuclease/phosphatase family protein
Mdem_scaffold_0275	439321	443857	K425_779			epsilon-sarcoglycan-like protein
Mdem_scaffold_0275	445292	446714	K425_891			mitochondrial ribosomal protein L35-like protein
Mdem_scaffold_0275	447222	449572	K425_892			mitochondrial ribosomal protein L45-like protein
Mdem_scaffold_0275	449742	455338	K425_780			phosphatidylinositol synthase-like protein
Mdem_scaffold_0275	451594	455363	K425_890			tRNA pseudouridine synthase
Mdem_scaffold_0275	455585	458557	K425_781			charged multivesicular body protein 7-like protein
Mdem_scaffold_0275	458537	462835	K425_889			Coagulation factor IX-like protein
Mdem_scaffold_0275	462963	467156	K425_782			Zinc finger, C2H2 type domain-containing protein
Mdem_scaffold_0275	468035	470012	K425_888			putative small nuclear ribonucleoprotein G
Mdem_scaffold_0275	470842	472779	K425_887			Anaphase-promoting complex subunit 10
Mdem_scaffold_0275	473852	478663	K425_886			carnation-like protein
Mdem_scaffold_0275	478881	480263	K425_783			phosphoglycerate mutase 1 family
Mdem_scaffold_0275	482236	488504	K425_885			putative methyltransferase
Mdem_scaffold_0275	491485	496868	K425_884			alpha-N-acetylgalactosaminidase-like protein
Mdem_scaffold_0275	498391	500316	K425_784			vps2-like protein
Mdem_scaffold_0275	500593	505288	K425_785			topoisomerase 3beta-like protein
Mdem_scaffold_0275	506117	516212	K425_786			rickets-like protein
Mdem_scaffold_0275	520788	529347	K425_883			protein tyrosine phosphatase-like protein
**Mdem_scaffold_0275**	**530097**	**530717**	**K425_787**	**lef-5**		**putative transcription initiation factor lef-5**
**Mdem_scaffold_0275**	**530749**	**532041**	**K425_788**	**vlf-1**	**synonym vlf-1a**	**very late factor 1, bracovirus capsid protein**
Mdem_scaffold_0275	537963	566096	K425_882			protein trachealess-like protein
**Mdem_scaffold_0275**	**616425**	**617597**	**K425_789**	**HzNVorf64-like (p51)**	**similar to HzNVorf64**	**Bracovirus particle protein HzNVorf64-like (p51)**
Mdem_scaffold_0275	623039	661917	K425_881			Nuclear envelope localisation domain-containing protein
Mdem_scaffold_0275	664354	667631	K425_880			Trophoblast glycoprotein-like protein
Mdem_scaffold_0275	669017	676025	K425_790			conserved uncharacterized protein
Mdem_scaffold_0275	677975	682010	K425_791			cytochrome P450 domain-containing protein
Mdem_scaffold_0275	682558	685431	K425_879			3-demethylubiquinone-9 3-O-methyltransferase
Mdem_scaffold_0275	687955	691108	K425_792			RILP-like protein
Mdem_scaffold_0275	693336	697724	K425_878			partner of drosha-like protein
Mdem_scaffold_0275	700118	706148	K425_877			tetraspanin-like protein
Mdem_scaffold_0275	707239	716124	K425_876			stromal interaction molecule-like protein
Mdem_scaffold_0275	717417	721669	K425_793			cramped-like protein
Mdem_scaffold_0275	723397	726241	K425_794			ETS-like proteinous factor
Mdem_scaffold_0275	724598	729744	K425_875			arginine methyltransferase 3-like protein
Mdem_scaffold_0275	729309	736996	K425_795			DNA repair and recombination protein RadA-like protein
Mdem_scaffold_0275	731434	735004	K425_796			asteroid-like protein
Mdem_scaffold_0275	736776	740835	K425_874			Annexin-B9
Mdem_scaffold_0275	741807	750537	K425_797			Palmitoyltransferase ZDHHC2
Mdem_scaffold_0275	751997	753955	K425_873			Putative hydroxypyruvate isomerase
Mdem_scaffold_0275	754117	756534	K425_872			abhydrolase domain-containing protein
Mdem_scaffold_0275	758313	766301	K425_871			monocarboxylate transporter 13-like protein
Mdem_scaffold_0275	766194	769045	K425_870			leucine-rich repeat-containing protein
Mdem_scaffold_0275	769387	773328	K425_798			transmembrane protein 39-like protein
Mdem_scaffold_0275	772125	775494	K425_869			NUDIX domain-containing protein
Mdem_scaffold_0275	776292	779563	K425_799			cytochrome c oxidase assembly protein COX15-like protein
Mdem_scaffold_0275	780775	784510	K425_868			ubiquitin domain-containing protein
Mdem_scaffold_0275	785351	791705	K425_801			MMS19 nucleotide excision repair-like protein
Mdem_scaffold_0275	791210	792631	K425_800			MORN repeat-containing protein
Mdem_scaffold_0275	795526	798572	K425_802			neuronal acetylcholine receptor subunit alpha-like protein
Mdem_scaffold_0275	798536	800627	K425_867			calmodulin-like protein 4-like protein
Mdem_scaffold_0275	802363	806989	K425_803			solute carrier family 25-like protein
Mdem_scaffold_0275	806550	808708	K425_866			mitotic checkpoint protein BUB3-like protein
Mdem_scaffold_0275	809108	813455	K425_804			ribosomal protein L4
Mdem_scaffold_0275	814596	816164	K425_805			pre-mRNA-splicing factor SPF27-like protein
Mdem_scaffold_0275	817235	831308	K425_865			colorectal mutant cancer-like protein
Mdem_scaffold_0275	833130	840544	K425_864			ETS domain-containing protein
Mdem_scaffold_0275	852701	856402	K425_806			solute carrier family 25-like protein
Mdem_scaffold_0275	856881	859279	K425_863			hypothetical protein
**Mdem_scaffold_0275**	**860238**	**862937**	**K425_862**	**lef-9**		**viral RNA polymerase subunit lef-9**

Mdem_scaffold_0275	863392	869217	K425_807			annexin-like protein
Mdem_scaffold_0275	874066	876108	K425_808			conserved uncharacterized protein
Mdem_scaffold_0275	878430	879719	K425_861			dopamine receptor 2-like protein
Mdem_scaffold_0275	907036	909835	K425_860			7tm chemosensory receptor
Mdem_scaffold_0275	910801	915478	K425_809		similar to venom protein 2 from Microctonus hyperodae	conserved uncharacterized protein family 3
Mdem_scaffold_0275	915381	916697	K425_859			BTB/POZ domain-containing protein
Mdem_scaffold_0275	928746	930547	K425_858			sprouty-like protein
Mdem_scaffold_0275	959630	1004377	K425_857			eukaryotic translation initiation factor 5B-like protein
Mdem_scaffold_0275	1003399	1006214	K425_810			ribosomal protein L27A, isoform A
Mdem_scaffold_0275	1006809	1007765	K425_856			splicing factor 3A subunit 2-like protein
Mdem_scaffold_0275	1010700	1011662	K425_855			Phosphatidylinositol-specific phospholipase C, X domain-containing protein
Mdem_scaffold_0275	1013130	1015692	K425_811		similar to venom protein Ci-48a from Chelonus inanitus	conserved uncharacterized protein family 3
Mdem_scaffold_0275	1015740	1017155	K425_812			hypothetical protein
Mdem_scaffold_0275	1019566	1020470	K425_813			conserved uncharacterized protein
Mdem_scaffold_0275	1025373	1027023	K425_814			conserved uncharacterized protein
Mdem_scaffold_0275	1027526	1034208	K425_854			conserved uncharacterized protein
Mdem_scaffold_0275	1035821	1039008	K425_815			conserved uncharacterized protein
Mdem_scaffold_0275	1041181	1048509	K425_853			monocarboxylate transporter 12-like protein
Mdem_scaffold_0275	1061081	1064062	K425_816			alpha-tubulin-like protein
Mdem_scaffold_0275	1065553	1066904	K425_817			conserved uncharacterized protein
Mdem_scaffold_0275	1067930	1074048	K425_818			MFS transporter domain-containing protein
Mdem_scaffold_0275	1076207	1080250	K425_852			adipocyte plasma membrane-associated-like protein
Mdem_scaffold_0275	1090524	1098177	K425_851			Double-stranded RNA-specific editase Adar-like protein
Mdem_scaffold_0275	1104369	1105878	K425_819			hypothetical protein
Mdem_scaffold_0275	1106640	1107784	K425_850			AN1-type zinc finger domain-containing protein
Mdem_scaffold_0275	1108479	1110516	K425_820		similar to GIP_L7_0080, GFP_L5_0240, GIP_L2_0090, GFP_L5_0230, GFP_L5_0070, GFP_L5_0090, GIP_L2_0100, GIP_L7_0040, GIP_L7_0010, and GFP_L5_0190	conserved uncharacterized protein family 7
Mdem_scaffold_0275	1111738	1115079	K425_821			disintegrin and metalloprotease-like protein
Mdem_scaffold_0275	1115574	1116578	K425_849			hypothetical protein
Mdem_scaffold_0275	1129347	1131659	K425_848			ribosome biogenesis protein BRX1-like protein
Mdem_scaffold_0275	1134352	1135701	K425_822			hypothetical protein
Mdem_scaffold_0275	1135700	1138191	K425_847			vascular endothelial growth factor receptor-like protein
Mdem_scaffold_0275	1140833	1142370	K425_823			hypothetical protein
Mdem_scaffold_0275	1142343	1146415	K425_846			anaphase-promoting complex subunit 4-like protein
Mdem_scaffold_0275	1150476	1155608	K425_824			putative reverse transcriptase
Mdem_scaffold_0275	1161034	1163400	K425_825			hypothetical protein
**Mdem_scaffold_0275**	**1169048**	**1173820**	**K425_826**	**helicase**		**putative viral DNA helicase and primase**
Mdem_scaffold_0275	1175110	1177308	K425_827			BTB/POZ domain-containing protein
Mdem_scaffold_0275	1178085	1179871	K425_828			BTB/POZ domain-containing protein
Mdem_scaffold_0275	1183124	1191645	K425_845			conserved uncharacterized protein
Mdem_scaffold_0275	1193865	1194953	K425_844		similar to Cotesia congregata Cc8L18.1 from p74 genomic region	conserved uncharacterized protein family 8
Mdem_scaffold_0275	1196093	1198158	K425_843			TAZ zinc finger domain-containing protein
Mdem_scaffold_0275	1199239	1201799	K425_842			TAZ zinc finger domain-containing protein
Mdem_scaffold_0275	1202631	1204641	K425_829			hypothetical protein
Mdem_scaffold_0275	1205148	1209163	K425_830			iduronate 2-sulfatase-like protein
Mdem_scaffold_0275	1209166	1221464	K425_841			Transcriptional regulator ATRX-like protein
Mdem_scaffold_0275	1224380	1226963	K425_840			Zinc finger, C2H2 type domain-containing protein
Mdem_scaffold_0275	1227396	1230457	K425_839			Zinc finger, C2H2 type domain-containing protein
Mdem_scaffold_0275	1228598	1232742	K425_831			non-structural maintenance of chromosomes element 1-like protein
Mdem_scaffold_0275	1233144	1235730	K425_832			Mitochondrial-processing peptidase subunit beta-like protein
Mdem_scaffold_0275	1235764	1238081	K425_838			Mps one binder kinase activator-like 3
Mdem_scaffold_0275	1239038	1243130	K425_837			HAUS augmin-like complex subunit 6 N-terminus domain-containing protein
Mdem_scaffold_0275	1242766	1247286	K425_833			kelch-like protein
Mdem_scaffold_0275	1247981	1252577	K425_836			dnaJ subfamily B member 12-like protein
Mdem_scaffold_0275	1252578	1260449	K425_834			conserved uncharacterized protein
Mdem_scaffold_0275	1263113	1264725	K425_835			nogo-B receptor-like protein
Mdem_scaffold_0331	7088	12518	K425_964		similar to venom protein 2 from Microctonus hyperodae	conserved uncharacterized protein family 3
Mdem_scaffold_0331	21310	24766	K425_949			RNA-binding protein 28-like protein
Mdem_scaffold_0331	25393	27549	K425_963			another transcription unit-like protein
Mdem_scaffold_0331	28990	32599	K425_950			prolactin regulatory element-binding-like protein
Mdem_scaffold_0331	33906	40499	K425_962			conserved uncharacterized protein family 9
Mdem_scaffold_0331	86270	87912	K425_961			hypothetical protein
Mdem_scaffold_0331	99849	122400	K425_960			conserved uncharacterized protein family 9
Mdem_scaffold_0331	135325	135984	K425_959			hypothetical protein
Mdem_scaffold_0331	144625	149689	K425_951			hypothetical protein
Mdem_scaffold_0331	156231	157613	K425_952			putative reverse transcriptase
Mdem_scaffold_0331	158532	159535	K425_953			putative reverse transcriptase
**Mdem_scaffold_0331**	**174370**	**188501**	**K425_954**		**synonym 97b**	**putative disintegrin and metalloprotease protein**
Mdem_scaffold_0331	192578	212093	K425_955			BEN-domain containing protein
Mdem_scaffold_0331	244471	245106	K425_958		similar to GIP_L7_0070 and CcBV_31.7 from Cotesia congregata bracovirus	putative reverse transcriptase and recombinase
Mdem_scaffold_0331	261227	278164	K425_957			adenylate cyclase type 3-like protein

Mdem_scaffold_0331	280226	290301	K425_956			alpha-tocopherol transfer-like protein
Mdem_scaffold_0359	5	1922	K425_996			hypothetical protein
**Mdem_scaffold_0359**	**8146**	**8628**	**K425_994**	**ank-V1**		**viral ankyrin**
**Mdem_scaffold_0359**	**8864**	**9775**	**K425_995**	**ptp-V2**		**protein tyrosine phosphatase**
**Mdem_scaffold_0359**	**10821**	**11913**	**K425_993**	**orph-V3**		**conserved hypothetical protein**
**Mdem_scaffold_0359**	**12169**	**15584**	**K425_976**	**ank-V4**		**viral ankyrin**
**Mdem_scaffold_0359**	**19958**	**23602**	**K425_992**	**orph-W1**		**conserved hypothetical protein family 5**
**Mdem_scaffold_0359**	**30049**	**31322**	**K425_977**	**orph-E3**		**conserved hypothetical protein**
**Mdem_scaffold_0359**	**32008**	**33170**	**K425_991**	**orph-E2**		**conserved hypothetical protein**
**Mdem_scaffold_0359**	**35045**	**36039**	**K425_978**	**orph-C3**		**hypothetical protein**
**Mdem_scaffold_0359**	**36580**	**37605**	**K425_990**	**orph-C4**		**conserved hypothetical protein**
**Mdem_scaffold_0359**	**39186**	**39710**	**K425_979**	**ank-C1**		**viral ankyrin**
**Mdem_scaffold_0359**	**40022**	**40567**	**K425_980**	**ank-C2**		**viral ankyrin**
**Mdem_scaffold_0359**	**42955**	**45295**	**K425_981**	**orph-X1**		**conserved hypothetical protein**
**Mdem_scaffold_0359**	**46350**	**47358**	**K425_982**	**orph-X2**		**conserved hypothetical protein**
**Mdem_scaffold_0359**	**48152**	**49201**	**K425_983**	**orph-X3**		**conserved hypothetical protein**
**Mdem_scaffold_0359**	**50374**	**52360**	**K425_989**	**orph-X4**	**similar to orph-E2**	**conserved hypothetical protein**
**Mdem_scaffold_0359**	**52561**	**55862**	**K425_984**	**orph-X5**		**conserved hypothetical protein family 5, BEN domain-containing**
Mdem_scaffold_0359	60928	64291	K425_985			clathrin light chain domain-containing protein
Mdem_scaffold_0359	64375	67785	K425_988			ankyrin repeat domain-containing protein
Mdem_scaffold_0359	68923	76583	K425_986			patatin-like phospholipase domain-containing protein
Mdem_scaffold_0359	80597	81036	K425_987			conserved uncharacterized protein
**Mdem_scaffold_0394**	**6743**	**10138**	**K425_1003**	**lef-8**		**viral RNA polymerase subunit**
**Mdem_scaffold_0394**	**52004**	**52660**	**K425_1000**	**HzNVorf128-like-1**		**HzNVorf128-like protein-1**
**Mdem_scaffold_0394**	**54625**	**57186**	**K425_1001**	**HzNVorf128-like-2**		**HzNVorf128-like protein-2**
Mdem_scaffold_0394	92890	92961	K425_998			tRNA-Ser
Mdem_scaffold_0394	98209	98280	K425_997			tRNA-Ser
Mdem_scaffold_0394	99149	99220	K425_999			tRNA-Ser
Mdem_scaffold_0394	142915	143597	K425_1002			hypothetical protein
Mdem_scaffold_0404	14473	17692	K425_1034			putative reverse transcriptase
Mdem_scaffold_0404	55800	73552	K425_1033			krotzkopf verkehrt-like protein
Mdem_scaffold_0404	79343	79414	K425_1005			tRNA-Ser
Mdem_scaffold_0404	79566	79637	K425_1004			tRNA-Ser
Mdem_scaffold_0404	90556	90627	K425_1006			tRNA-Ser
Mdem_scaffold_0404	92307	95038	K425_1032			hypothetical protein
Mdem_scaffold_0404	104758	111142	K425_1031			krotzkopf verkehrt-like protein
Mdem_scaffold_0404	129629	132889	K425_1007			reverse transcriptase-like protein
Mdem_scaffold_0404	145002	159464	K425_1008			transporter, solute:sodium symporter (SSS) family
Mdem_scaffold_0404	183316	184791	K425_1009			conserved uncharacterized protein family 10
Mdem_scaffold_0404	186651	189606	K425_1010			conserved uncharacterized protein family 10
Mdem_scaffold_0404	194789	195791	K425_1011			conserved uncharacterized protein family 10
Mdem_scaffold_0404	217855	231614	K425_1030			E3 ubiquitin-protein ligase RNF25-like protein
Mdem_scaffold_0404	252603	254946	K425_1029			pterin-4a-carbinolamine dehydratase-like protein
Mdem_scaffold_0404	256406	260414	K425_1012			DNA-directed RNA polymerase I subunit RPA49-like protein
Mdem_scaffold_0404	261902	269951	K425_1013			WW domain-containing oxidoreductase
Mdem_scaffold_0404	270419	277817	K425_1028			mitochondrial ribosomal protein L15
Mdem_scaffold_0404	280047	288227	K425_1014			heparan sulfate 2-O-sulfotransferase-like protein
Mdem_scaffold_0404	284110	289672	K425_1027			N-terminal methyltransferase-like protein
Mdem_scaffold_0404	298674	302982	K425_1026			NADH dehydrogenase ubiquinone 1 alpha subcomplex subunit-like protein
Mdem_scaffold_0404	304458	317625	K425_1015			ftsJ methyltransferase domain-containing protein 1-like protein
Mdem_scaffold_0404	324589	338425	K425_1025			decaprenyl-diphosphate synthase subunit 2-like protein
Mdem_scaffold_0404	344025	363183	K425_1016			vacuolar protein sorting-associated protein VTA1-like protein
Mdem_scaffold_0404	377584	378300	K425_1017			putative reverse transcriptase
Mdem_scaffold_0404	379806	381349	K425_1018			conserved uncharacterized protein
**Mdem_scaffold_0404**	**384450**	**386375**	**K425_1024**	**odv-e66-11**		**putative envelope protein ODV-E66-11**
Mdem_scaffold_0404	396473	399357	K425_1023			condensin complex subunit 2-like protein
Mdem_scaffold_0404	399580	401880	K425_1019			putative palmitoyltransferase ZDHHC24-like protein
Mdem_scaffold_0404	411345	412838	K425_1020			conserved uncharacterized protein
Mdem_scaffold_0404	412896	413891	K425_1021			putative reverse transcriptase
Mdem_scaffold_0404	483574	486028	K425_1022			conserved uncharacterized protein
Mdem_scaffold_0407	3553	15755	K425_1224			g1/S-specific cyclin-D2-like protein
Mdem_scaffold_0407	50358	55857	K425_1223			conserved uncharacterized protein
Mdem_scaffold_0407	50416	51641	K425_1037			cell cycle control protein cwf16-like protein
Mdem_scaffold_0407	55606	66122	K425_1038			spondin-like protein
Mdem_scaffold_0407	66113	67652	K425_1222			translation initiation factor-3 (IF3), putative
Mdem_scaffold_0407	67426	68716	K425_1039			conserved uncharacterized protein
Mdem_scaffold_0407	68571	69975	K425_1221			conserved uncharacterized protein
Mdem_scaffold_0407	71384	74204	K425_1220			RING finger domain-containing protein
Mdem_scaffold_0407	74979	77364	K425_1219			glucose dehydrogenase acceptor-like protein
Mdem_scaffold_0407	78579	82896	K425_1218			glucose dehydrogenase acceptor-like protein
Mdem_scaffold_0407	83841	87035	K425_1217			glucose dehydrogenase acceptor-like protein
Mdem_scaffold_0407	88466	93299	K425_1216			glucose dehydrogenase acceptor-like protein
Mdem_scaffold_0407	93974	96542	K425_1215			glucose dehydrogenase acceptor-like protein
Mdem_scaffold_0407	97171	99048	K425_1214			glucose dehydrogenase acceptor-like protein
Mdem_scaffold_0407	99849	101726	K425_1213			glucose dehydrogenase acceptor-like protein
Mdem_scaffold_0407	102228	104111	K425_1212			glucose dehydrogenase acceptor-like protein
Mdem_scaffold_0407	104682	106526	K425_1211			glucose dehydrogenase acceptor-like protein
Mdem_scaffold_0407	107118	108983	K425_1210			glucose dehydrogenase acceptor-like protein
Mdem_scaffold_0407	109643	111520	K425_1209			glucose dehydrogenase acceptor-like protein
Mdem_scaffold_0407	112048	113604	K425_1208			glucose dehydrogenase acceptor-like protein
Mdem_scaffold_0407	115236	119495	K425_1207			glucose dehydrogenase acceptor-like protein
Mdem_scaffold_0407	118767	122600	K425_1040			flotillin-2-like protein

Mdem_scaffold_0407	126887	140923	K425_1205			Phosphorylase b kinase gamma catalytic chain, skeletal muscle-like protein
Mdem_scaffold_0407	141449	144373	K425_1206			phosphorylase kinase gamma-like protein
Mdem_scaffold_0407	152053	156126	K425_1204			arginine--tRNA ligase
Mdem_scaffold_0407	155011	162808	K425_1041			adenylate cyclase type 9-like protein
Mdem_scaffold_0407	163358	170123	K425_1203			chaoptin-like protein
Mdem_scaffold_0407	171515	179491	K425_1202			silver-like protein
Mdem_scaffold_0407	179862	184065	K425_1042			shawn-like protein
Mdem_scaffold_0407	180106	181555	K425_1201			Coiled-coil domain-containing protein
Mdem_scaffold_0407	184073	185866	K425_1043			transcriptional adapter 3-B-like protein
Mdem_scaffold_0407	186961	189745	K425_1200			ATP synthase lipid-binding protein, mitochondrial-like protein
Mdem_scaffold_0407	189745	191332	K425_1044			conserved uncharacterized protein
Mdem_scaffold_0407	196614	199530	K425_1199			AN1-type zinc finger protein 6-like protein
Mdem_scaffold_0407	203503	208330	K425_1045			YL-1-like protein
Mdem_scaffold_0407	207945	213796	K425_1198			ras-related protein Rab-11A-like protein
Mdem_scaffold_0407	213964	218413	K425_1046			post-GPI attachment to proteins factor 2-like protein
Mdem_scaffold_0407	274662	288908	K425_1047			scalloped-like protein
Mdem_scaffold_0407	298940	307159	K425_1197			caudal-like protein
Mdem_scaffold_0407	316878	321380	K425_1048			conserved uncharacterized protein
Mdem_scaffold_0407	321358	323835	K425_1196			conserved uncharacterized protein
Mdem_scaffold_0407	324230	326296	K425_1049			lipase 3-like protein
Mdem_scaffold_0407	326672	328839	K425_1050			lipase 3-like protein
Mdem_scaffold_0407	329359	340300	K425_1051			guanine nucleotide exchange factor for Rab-3A-like protein
Mdem_scaffold_0407	341223	343740	K425_1195			conserved uncharacterized protein
Mdem_scaffold_0407	345330	351916	K425_1194			supernumerary limbs-like protein
Mdem_scaffold_0407	352427	354165	K425_1193			phosphatidate phosphatase PPAPDC1A-like protein
Mdem_scaffold_0407	353530	358949	K425_1052			Abhydrolase domain-containing protein
Mdem_scaffold_0407	358828	360213	K425_1192			crossover junction endonuclease EME1-like protein
Mdem_scaffold_0407	362566	365722	K425_1191			protein SAAL1-like protein
Mdem_scaffold_0407	364483	373899	K425_1053			disintegrin and metalloproteinase domain-containing protein
Mdem_scaffold_0407	375411	377969	K425_1190			transmembrane protein 129-like protein
Mdem_scaffold_0407	499220	505085	K425_1054			conserved uncharacterized protein
Mdem_scaffold_0407	507653	513193	K425_1189			glycosyltransferase-like protein LARGE1
Mdem_scaffold_0407	513701	520640	K425_1187			extracellular domains-containing protein CG31004-like protein
Mdem_scaffold_0407	521445	523705	K425_1188			Pop2-like protein
Mdem_scaffold_0407	524522	529077	K425_1055			BTB/POZ domain-containing protein KCTD9-like protein
Mdem_scaffold_0407	553456	555659	K425_1056			hypothetical protein
Mdem_scaffold_0407	556773	558781	K425_1057			hypothetical protein
Mdem_scaffold_0407	593514	595594	K425_1186			MFS sugar transporter domain-containing protein
Mdem_scaffold_0407	623796	629475	K425_1058			octopamine receptor beta-2R-like protein
Mdem_scaffold_0407	636511	640741	K425_1059			conserved uncharacterized protein
Mdem_scaffold_0407	638673	646530	K425_1185			DNA-directed RNA polymerase II subunit RPB2-like protein
Mdem_scaffold_0407	647994	649391	K425_1060			thioredoxin-dependent peroxide reductase, mitochondrial-like protein
Mdem_scaffold_0407	660088	661165	K425_1184			Homeobox domain-containing protein
Mdem_scaffold_0407	692223	696649	K425_1183			EB module-containing protein
Mdem_scaffold_0407	704420	710970	K425_1182			Homeobox domain-containing protein
Mdem_scaffold_0407	734925	736286	K425_1061			mitochondrial ribosomal protein L2
Mdem_scaffold_0407	735481	739534	K425_1181			sideroflexin-1-like protein
Mdem_scaffold_0407	743183	745719	K425_1062			osiris 1-like protein
Mdem_scaffold_0407	748246	752632	K425_1063			osiris 24-like protein
Mdem_scaffold_0407	754200	757541	K425_1064			osiris 2-like protein
Mdem_scaffold_0407	762068	764056	K425_1065			osiris 3-like protein
Mdem_scaffold_0407	776743	778299	K425_1066			osiris 6-like protein
Mdem_scaffold_0407	785803	789079	K425_1067			osiris 7-like protein
Mdem_scaffold_0407	795427	796838	K425_1069			osiris 8-like protein
Mdem_scaffold_0407	800732	802186	K425_1068			osiris 9-like protein
Mdem_scaffold_0407	803327	804871	K425_1070			osiris 10-like protein
Mdem_scaffold_0407	805458	808224	K425_1071			osiris 11-like protein
Mdem_scaffold_0407	809411	811394	K425_1180			osiris 11-like protein
Mdem_scaffold_0407	810240	810313	K425_1036			tRNA-Thr
Mdem_scaffold_0407	816858	818333	K425_1072			osiris 12-like protein
Mdem_scaffold_0407	827071	829041	K425_1073			osiris 14-like protein
Mdem_scaffold_0407	838269	841579	K425_1074			osiris 16-like protein
Mdem_scaffold_0407	846642	853388	K425_1075			osiris 17-like protein
Mdem_scaffold_0407	853370	853459	K425_1035			tRNA-Ile
Mdem_scaffold_0407	854463	855643	K425_1179			Odorant-binding protein 56-like protein
Mdem_scaffold_0407	856373	857824	K425_1178			Odorant-binding protein 56-like protein
Mdem_scaffold_0407	860594	862487	K425_1077			Odorant-binding protein 56-like protein
Mdem_scaffold_0407	862825	864425	K425_1076			Odorant-binding protein 56-like protein
Mdem_scaffold_0407	865755	867091	K425_1177			osiris 20-like protein
Mdem_scaffold_0407	869283	870458	K425_1175			osiris 19-like protein
Mdem_scaffold_0407	872566	873613	K425_1176			osiris 18-like protein
Mdem_scaffold_0407	874601	877476	K425_1078			conserved uncharacterized protein family 3
Mdem_scaffold_0407	878508	881134	K425_1079			conserved uncharacterized protein
Mdem_scaffold_0407	879655	880932	K425_1174			ankyrin-repeat domain-containing protein
Mdem_scaffold_0407	881472	882736	K425_1173			conserved uncharacterized protein
Mdem_scaffold_0407	883353	887623	K425_1080			general transcription factor IIH subunit 3-like protein
Mdem_scaffold_0407	929075	930914	K425_1172			octopamine receptor alpha-1B-like protein
Mdem_scaffold_0407	946939	947937	K425_1171			conserved uncharacterized protein
Mdem_scaffold_0407	1001558	1003024	K425_1170			octopamine receptor alpha-1B-like protein, partial
Mdem_scaffold_0407	1052912	1058686	K425_1081			transporter, solute:sodium symporter (SSS) family
Mdem_scaffold_0407	1063363	1075192	K425_1082		similar to GfV-B16-ORF1-like protein	conserved uncharacterized protein family 6
Mdem_scaffold_0407	1124654	1129685	K425_1083			sorting nexin-4-like protein

Mdem_scaffold_0407	1133670	1149986	K425_1169			pyrokinin 1 receptor-like protein
Mdem_scaffold_0407	1156678	1162916	K425_1168			Neutral ceramidase-like protein
Mdem_scaffold_0407	1172993	1174595	K425_1084			cuticle-like protein
Mdem_scaffold_0407	1176263	1177108	K425_1085			cuticle-like protein
Mdem_scaffold_0407	1180140	1180921	K425_1086			cuticle-like protein
Mdem_scaffold_0407	1181331	1185656	K425_1167			Neutral ceramidase-like protein
Mdem_scaffold_0407	1189130	1199193	K425_1087			serine protease nudel-like protein
Mdem_scaffold_0407	1204570	1232948	K425_1088			uninflatable-like protein
Mdem_scaffold_0407	1234303	1235960	K425_1089			pomp-like protein
Mdem_scaffold_0407	1235959	1239348	K425_1166			n-acetyllactosaminide beta-1,3-N-acetylglucosaminyltransferase-like protein
Mdem_scaffold_0407	1239180	1242154	K425_1090			conserved uncharacterized protein
Mdem_scaffold_0407	1240310	1243691	K425_1165			Haloacid dehalogenase-like hydrolase domain-containing protein
Mdem_scaffold_0407	1244171	1250817	K425_1164			ubiquitin thioesterase trabid-like protein, partial
Mdem_scaffold_0407	1253105	1259047	K425_1163			dihydroxyacetone phosphate acyltransferase-like protein
Mdem_scaffold_0407	1259359	1262521	K425_1091			pinin-like protein
Mdem_scaffold_0407	1279076	1280688	K425_1162			conserved uncharacterized protein
Mdem_scaffold_0407	1281333	1282949	K425_1092			conserved uncharacterized protein
Mdem_scaffold_0407	1281969	1285201	K425_1161			4-hydroxybenzoate polyprenyl transferase
Mdem_scaffold_0407	1285338	1289135	K425_1093			Abhydrolase domain-containing protein
Mdem_scaffold_0407	1289894	1300024	K425_1094			la-related protein 1-like protein
Mdem_scaffold_0407	1313349	1316693	K425_1160			Heart- and neural crest derivatives-expressed protein 2-like protein
Mdem_scaffold_0407	1324463	1327272	K425_1159			UMP-CMP kinase-like protein
Mdem_scaffold_0407	1329834	1332574	K425_1158			pointed-like protein
Mdem_scaffold_0407	1339535	1342204	K425_1157			pointed-like protein
Mdem_scaffold_0407	1384530	1388592	K425_1095			mitochondrial ATP synthase coupling factor 6-like protein
Mdem_scaffold_0407	1389086	1392437	K425_1096			conserved uncharacterized protein
**Mdem_scaffold_0407**	**1392006**	**1393626**	**K425_1156**	**lef-4**		**viral RNA polymerase subunit lef-4**
Mdem_scaffold_0407	1402394	1406469	K425_1155			Endoribonuclease XendoU domain-containing protein
Mdem_scaffold_0407	1406581	1409732	K425_1154			glyoxalase domain-containing protein 4-like protein
Mdem_scaffold_0407	1410036	1416206	K425_1097			proline--tRNA ligase
Mdem_scaffold_0407	1416218	1423469	K425_1153			DNA polymerase epsilon catalytic subunit A-like protein
Mdem_scaffold_0407	1423789	1425949	K425_1152			conserved uncharacterized protein
Mdem_scaffold_0407	1432100	1435504	K425_1098			ubiquitin carboxyl-terminal hydrolase 46-like protein
Mdem_scaffold_0407	1435513	1436638	K425_1151			60S ribosomal protein L21
Mdem_scaffold_0407	1436822	1437889	K425_1150			60S ribosomal protein L21
Mdem_scaffold_0407	1446863	1451768	K425_1148			Bardet-Biedl syndrome 5-like protein
Mdem_scaffold_0407	1447024	1448283	K425_1099			conserved uncharacterized protein
Mdem_scaffold_0407	1451868	1455993	K425_1100			SCY1-like protein 2-like protein
Mdem_scaffold_0407	1456676	1460183	K425_1101			xaa-Pro aminopeptidase 1-like protein
Mdem_scaffold_0407	1464799	1471631	K425_1147			conserved uncharacterized protein
Mdem_scaffold_0407	1495630	1498182	K425_1102			nuclear receptor-binding factor 2-like protein
Mdem_scaffold_0407	1498976	1499951	K425_1146			putative odorant receptor
Mdem_scaffold_0407	1506924	1508550	K425_1145			putative odorant receptor
Mdem_scaffold_0407	1510311	1515599	K425_1103			conserved uncharacterized protein
Mdem_scaffold_0407	1515733	1518846	K425_1144			dead end-like protein
Mdem_scaffold_0407	1589808	1603155	K425_1104			mushroom body large-type Kenyon cell-specific-like protein
Mdem_scaffold_0407	1624163	1628728	K425_1105			pyruvate dehydrogenase complex dihydrolipoamide acetyltransferase
Mdem_scaffold_0407	1629497	1632609	K425_1143			serine/threonine-protein kinase PAK 1-like protein
Mdem_scaffold_0407	1633385	1637030	K425_1106			merlin-like protein
Mdem_scaffold_0407	1637521	1639682	K425_1107			2-methoxy-6-polyprenyl-1,4-benzoquinol methylase, mitochondrial-like protein
Mdem_scaffold_0407	1640121	1642312	K425_1142			cytoplasmic dynein light chain 2-like protein
Mdem_scaffold_0407	1643503	1646867	K425_1108			serine/arginine-rich splicing factor 1-like protein
Mdem_scaffold_0407	1649504	1653908	K425_1109			whacked-like protein
Mdem_scaffold_0407	1653909	1657469	K425_1141			BRCA1-associated-like protein
Mdem_scaffold_0407	1656954	1660401	K425_1110			putative neutral sphingomyelinase-like protein
Mdem_scaffold_0407	1658263	1668639	K425_1140			rho GTPase-activating protein 10-like protein
Mdem_scaffold_0407	1669702	1675748	K425_1112			branched-chain amino acid aminotransferase
Mdem_scaffold_0407	1676865	1684825	K425_1111			octopamine receptor beta-1R-like protein
Mdem_scaffold_0407	1680673	1682622	K425_1139			hypothetical protein
Mdem_scaffold_0407	1687019	1690080	K425_1138			NAD-dependent deacetylase sirtuin-2-like protein
Mdem_scaffold_0407	1689056	1703094	K425_1113			vacuolar protein sorting-associated protein 13B-like protein
Mdem_scaffold_0407	1702818	1704602	K425_1136			clathrin coat assembly protein ap17-like protein
Mdem_scaffold_0407	1704992	1705937	K425_1114			Trafficking protein particle complex subunit 6B-like protein
Mdem_scaffold_0407	1705938	1710689	K425_1137			rab proteins geranylgeranyltransferase component A-like protein
Mdem_scaffold_0407	1712064	1716313	K425_1115			calnexin-like protein
Mdem_scaffold_0407	1716314	1720039	K425_1135			myotubularin-related protein 8-like protein
Mdem_scaffold_0407	1721297	1726407	K425_1116			Brain tumor-like protein
Mdem_scaffold_0407	1735748	1738398	K425_1134			ric8a-like protein
Mdem_scaffold_0407	1738399	1740221	K425_1117			TGF-beta-activated kinase 1 and MAP3K7-binding-like protein
Mdem_scaffold_0407	1740946	1744442	K425_1133			4-hydroxybutyrate coenzyme A transferase
Mdem_scaffold_0407	1744917	1747967	K425_1118			V-type ATPase, B subunit
Mdem_scaffold_0407	1749526	1755927	K425_1119			glycerol-3-phosphate acyltransferase 1, mitochondrial-like protein
Mdem_scaffold_0407	1755932	1758312	K425_1132			tektin-like protein
Mdem_scaffold_0407	1758680	1761081	K425_1120			tektin-like protein
Mdem_scaffold_0407	1780179	1794502	K425_1121			empty spiracles-like protein
Mdem_scaffold_0407	1864494	1868625	K425_1122			Bromodomain-containing protein
Mdem_scaffold_0407	1868954	1869991	K425_1123			clavesin-2-like protein
Mdem_scaffold_0407	1871938	1874170	K425_1124			clavesin-2-like protein
Mdem_scaffold_0407	1874764	1877474	K425_1125			cuticular protein 57A-like protein
Mdem_scaffold_0407	1876039	1878588	K425_1131			synaptosomal-associated protein 29-like protein
Mdem_scaffold_0407	1879244	1881866	K425_1126			lactosylceramide 4-alpha-galactosyltransferase-like protein

Mdem_scaffold_0407	1886315	1900782	K425_1130			twins-like protein
Mdem_scaffold_0407	1901565	1907675	K425_1129			conserved uncharacterized protein
Mdem_scaffold_0407	1907839	1909172	K425_1127			Phosphoglycerate mutase family member 5-like protein
Mdem_scaffold_0407	1912958	1916670	K425_1128			tyrosine-protein kinase Btk29A-like protein
Mdem_scaffold_0412	525	6328	K425_1346			ALK tyrosine kinase receptor-like protein
Mdem_scaffold_0412	10520	12270	K425_1314			ribonuclease H2 subunit C-like protein
Mdem_scaffold_0412	12257	16839	K425_1345			Transmembrane channel-like protein 7
Mdem_scaffold_0412	16600	19337	K425_1315			PPPDE peptidase domain-containing protein 1
**Mdem_scaffold_0412**	**21239**	**23986**	**K425_1323**	**orph-H1**		**conserved uncharacterized protein**
**Mdem_scaffold_0412**	**21917**	**23659**	**K425_1340**	**ank-H4**	**synonym IkB-H1**	**viral ankyrin**
**Mdem_scaffold_0412**	**23926**	**24888**	**K425_1341**	**ptp-H3**		**protein tyrosine phosphatase**
**Mdem_scaffold_0412**	**25337**	**26983**	**K425_1316**	**ptp-H2**		**protein tyrosine phosphatase**
**Mdem_scaffold_0412**	**27569**	**28579**	**K425_1342**	**ptp-H1**		**protein tyrosine phosphatase**
**Mdem_scaffold_0412**	**29700**	**30659**	**K425_1343**	**ptp-H5**		**protein tyrosine phosphatase**
**Mdem_scaffold_0412**	**31477**	**32025**	**K425_1344**	**ptp-H4**		**protein tyrosine phosphatase**
Mdem_scaffold_0412	38285	48737	K425_1339			genghis khan-like protein
Mdem_scaffold_0412	49104	50685	K425_1317			sorting nexin-24-like protein
Mdem_scaffold_0412	56573	80993	K425_1338			semaphorin-1A-like protein
Mdem_scaffold_0412	161818	165147	K425_1337			Abhydrolase domain-containing protein
Mdem_scaffold_0412	165572	166947	K425_1336			SUN domain-containing-like protein
Mdem_scaffold_0412	166525	172129	K425_1318			Zinc finger FYVE domain-containing protein
Mdem_scaffold_0412	169594	176060	K425_1335			BAG domain-containing protein
Mdem_scaffold_0412	183868	187675	K425_1334			HEAT repeat-containing protein
Mdem_scaffold_0412	188107	191063	K425_1319			zucchini-like protein
Mdem_scaffold_0412	189806	191427	K425_1333			peroxisomal membrane protein 11-like protein
Mdem_scaffold_0412	191154	195413	K425_1320			non-lysosomal glucosylceramidase-like protein
Mdem_scaffold_0412	195195	199387	K425_1332			cytochrome P450 domain-containing protein
Mdem_scaffold_0412	203905	206991	K425_1321			pro-resilin-like protein, partial
Mdem_scaffold_0412	209017	209829	K425_1331			pro-resilin-like protein
Mdem_scaffold_0412	220403	221030	K425_1330			pro-resilin-like protein
Mdem_scaffold_0412	222808	226121	K425_1329			pro-resilin-like protein, partial
Mdem_scaffold_0412	236501	238147	K425_1328			pro-resilin-like protein
Mdem_scaffold_0412	260968	263754	K425_1327			pro-resilin-like protein, partial
Mdem_scaffold_0412	270135	271607	K425_1326			pro-resilin-like protein
Mdem_scaffold_0412	285908	292502	K425_1325			hypothetical protein
Mdem_scaffold_0412	311239	321158	K425_1324			conserved uncharacterized protein
Mdem_scaffold_0412	360440	394948	K425_1322			hemicentin-1-like protein
Mdem_scaffold_0435	8125	10105	K425_1347			EB module-containing protein
Mdem_scaffold_0435	14900	19898	K425_1348			carboxylesterase domain-containing protein
Mdem_scaffold_0435	24098	24889	K425_1349			carboxylesterase domain-containing protein
**Mdem_scaffold_0435**	**27844**	**29110**	**K425_1357**	**ac92-like-2**	**similar to HzNVorf13**	**ac92-like protein, putative sulfhydryl oxidase-2**
Mdem_scaffold_0435	30798	31310	K425_1356			putative reverse transcriptase
Mdem_scaffold_0435	31335	32008	K425_1355		similar to hypothetical protein Cotesia conregata bracovirus CcBV_2.3	conserved uncharacterized protein
Mdem_scaffold_0435	32387	33358	K425_1354		similar to hypothetical protein Cotesia conregata bracovirus CcBV_2.3	conserved uncharacterized protein
Mdem_scaffold_0435	34225	35034	K425_1350			hypothetical protein
Mdem_scaffold_0435	39651	41123	K425_1351			putative reverse transcriptase
Mdem_scaffold_0435	41896	43026	K425_1353			hypothetical protein
Mdem_scaffold_0435	44348	61979	K425_1352			katanin 60-like protein
Mdem_scaffold_0446	7491	14396	K425_1358			ribose-phosphate diphosphokinase-like protein
Mdem_scaffold_0446	15523	23916	K425_1359			RHO protein GDP dissociation inhibitor domain-containing protein
Mdem_scaffold_0446	36373	41947	K425_1360			singed-like protein
Mdem_scaffold_0446	43847	55414	K425_1369			ribonuclease Z, mitochondrial-like protein
Mdem_scaffold_0446	56195	58721	K425_1361			hypothetical protein
Mdem_scaffold_0446	68589	71086	K425_1368			UDP-glucoronosyl and UDP-glucosyl transferase domain-containing protein
**Mdem_scaffold_0446**	**79263**	**79535**	**K425_1367**	**orph-R1**		**conserved hypothetical protein**
Mdem_scaffold_0446	86089	86364	K425_1366			histone H4 replacement-like protein
Mdem_scaffold_0446	86570	87155	K425_1362			histone H3-like protein
Mdem_scaffold_0446	86893	87159	K425_1365			histone H3-like protein
Mdem_scaffold_0446	90079	90680	K425_1364			histone H2B-like protein
Mdem_scaffold_0446	90470	91283	K425_1363			histone H2A-like protein
Mdem_scaffold_0528	99881	124927	K425_1503			mushroom-body expressed-like protein
Mdem_scaffold_0528	145754	149257	K425_1541			alpha-tubulin-like protein
Mdem_scaffold_0528	152617	153591	K425_1540			conserved uncharacterized protein
Mdem_scaffold_0528	153864	153939	K425_1501			tRNA-Arg
Mdem_scaffold_0528	157132	158739	K425_1539			hypothetical protein
Mdem_scaffold_0528	160211	163528	K425_1538			hypothetical protein
Mdem_scaffold_0528	161621	167941	K425_1504			rough-like protein
Mdem_scaffold_0528	173251	179239	K425_1537			conserved uncharacterized protein
Mdem_scaffold_0528	181415	183759	K425_1505			tolkin, partial
Mdem_scaffold_0528	187700	194057	K425_1506			tolkin, partial
Mdem_scaffold_0528	195209	196853	K425_1507			tolkin, partial
Mdem_scaffold_0528	197420	197978	K425_1508			tolloid, partial
Mdem_scaffold_0528	242044	250682	K425_1509			Angiotensin-converting enzyme-like protein
Mdem_scaffold_0528	250859	252889	K425_1535			cuticular-like protein
Mdem_scaffold_0528	256103	259840	K425_1536			cuticular-like protein
Mdem_scaffold_0528	267238	270415	K425_1534			cuticular-like protein
Mdem_scaffold_0528	287844	303637	K425_1510			conserved uncharacterized protein
Mdem_scaffold_0528	304900	308317	K425_1511			egf domain-containing protein
Mdem_scaffold_0528	309679	323617	K425_1512			conserved uncharacterized protein
Mdem_scaffold_0528	325486	326274	K425_1513			7tm chemosensory receptor domain-containing protein

Mdem_scaffold_0528	327277	332347	K425_1533			transmembrane protein 161AB-like protein
Mdem_scaffold_0528	333250	335490	K425_1532			chitooligosaccharidolytic beta-N-acetylglucosaminidase-like protein
Mdem_scaffold_0528	342533	348030	K425_1514			conserved uncharacterized protein
Mdem_scaffold_0528	348493	350507	K425_1515			ankyrin-repeat domain-containing protein
Mdem_scaffold_0528	350655	357027	K425_1531			EB module-containing protein
Mdem_scaffold_0528	367531	368186	K425_1530			hypothetical protein
Mdem_scaffold_0528	386894	393242	K425_1529			headcase-like protein
Mdem_scaffold_0528	413650	413721	K425_1502			tRNA-Asp
Mdem_scaffold_0528	478552	478626	K425_1500			tRNA-Lys
Mdem_scaffold_0528	504799	505377	K425_1528			headcase-like protein
Mdem_scaffold_0528	567181	568810	K425_1527		similar to egf1.5	Trypsin Inhibitor like cysteine rich domain-containing protein
Mdem_scaffold_0528	570273	571852	K425_1526		similar to egf1.5	Trypsin Inhibitor like cysteine rich domain-containing protein
Mdem_scaffold_0528	586059	606389	K425_1525			putative dynein heavy chain, ciliary-like protein
**Mdem_scaffold_0528**	**629227**	**630062**	**K425_1524**	**17a**	**similar to Chelonus inanitus 17a and Cotesia congregata hypothetical protein CBL47422**	**Bracovirus particle protein 17a**
Mdem_scaffold_0528	630639	633578	K425_1523			trypsin-like protein
Mdem_scaffold_0528	634554	641460	K425_1516			TAF5-like protein RNA polymerase II p300-like protein
Mdem_scaffold_0528	643114	653552	K425_1522			elongin A-like protein
Mdem_scaffold_0528	645455	647261	K425_1517			putative reverse transcriptase
Mdem_scaffold_0528	655277	661836	K425_1518			glutamate dehydrogenase-like protein
Mdem_scaffold_0528	664771	672626	K425_1519			serpin-like protein
Mdem_scaffold_0528	718457	729790	K425_1520			hypothetical protein
Mdem_scaffold_0528	752535	759136	K425_1521			conserved uncharacterized protein
Mdem_scaffold_0617	5302	18100	K425_1543			Ribosome 60S biogenesis N-terminal domain-containing protein
Mdem_scaffold_0617	17824	19681	K425_1557			conserved uncharacterized protein family 11
Mdem_scaffold_0617	23720	25289	K425_1556			conserved uncharacterized protein family 11
Mdem_scaffold_0617	34901	49515	K425_1544			ankyrin-repeat domain-containing protein
Mdem_scaffold_0617	55976	56048	K425_1542			tRNA-Arg
Mdem_scaffold_0617	67826	68491	K425_1555			ankyrin-repeat domain-containing protein
Mdem_scaffold_0617	126180	130371	K425_1545			ankyrin-repeat domain-containing protein
Mdem_scaffold_0617	130426	133655	K425_1554			tRNA-i(6)A37 thiotransferase enzyme MiaB
Mdem_scaffold_0617	134081	138752	K425_1553			apolipoprotein O-like protein
Mdem_scaffold_0617	141408	142269	K425_1552			hypothetical protein
Mdem_scaffold_0617	142865	144213	K425_1551			conserved uncharacterized protein
Mdem_scaffold_0617	146619	158137	K425_1546			importin-5-like protein
**Mdem_scaffold_0617**	**161773**	**163900**	**K425_1547**	**odv-e66-12**		**putative envelope protein ODV-E66-12**
Mdem_scaffold_0617	173325	178177	K425_1550			conserved uncharacterized protein
Mdem_scaffold_0617	212394	213548	K425_1549			conserved uncharacterized protein
Mdem_scaffold_0617	238221	240177	K425_1548			conserved uncharacterized protein
Mdem_scaffold_0844	18342	21221	K425_1593			cysteine desulfurase, mitochondrial-like protein
Mdem_scaffold_0844	21502	23153	K425_1611			ras-related protein Rab-X6-like protein
Mdem_scaffold_0844	23689	25592	K425_1594			hypothetical protein
Mdem_scaffold_0844	25982	28053	K425_1595			ankyrin-repeat domain-containing protein
Mdem_scaffold_0844	28748	34819	K425_1610			translation initiation factor eIF-2B subunit delta-like protein
Mdem_scaffold_0844	35829	37900	K425_1609			ribosomal protein L19-like protein
Mdem_scaffold_0844	38190	47931	K425_1596			peroxisomal acyl-coenzyme A oxidase-like protein
Mdem_scaffold_0844	46699	48384	K425_1608			RCC1 domain-containing protein
Mdem_scaffold_0844	55123	70845	K425_1607			syndecan-like protein
Mdem_scaffold_0844	102577	108042	K425_1606			ATPase inhibitor mai-2, mitochondrial-like protein
Mdem_scaffold_0844	106443	111178	K425_1597			scarlet-like protein
Mdem_scaffold_0844	114338	115116	K425_1598			hypothetical protein
Mdem_scaffold_0844	116850	117716	K425_1599			hypothetical protein
Mdem_scaffold_0844	118546	126906	K425_1605			Ras-related protein Rab-32-like protein
**Mdem_scaffold_0844**	**137170**	**138240**	**K425_1604**	**odv-e66-14**		**putative envelope protein ODV-E66-14, partial**
**Mdem_scaffold_0844**	**138691**	**139500**	**K425_1603**	**odv-e66-15**		**putative envelope protein ODV-E66-15, partial**
**Mdem_scaffold_0844**	**139688**	**140497**	**K425_1602**	**odv-e66-16**		**putative envelope protein ODV-E66-16, partial**
Mdem_scaffold_0844	143172	153804	K425_1600			flapwing-like protein
Mdem_scaffold_0844	169740	181029	K425_1601			conserved uncharacterized protein
Mdem_scaffold_0845	5788	15606	K425_1613			putative alpha-amylase
**Mdem_scaffold_0845**	**20016**	**21586**	**K425_1612**	**pif-5-4**	**synonym odv-e56**	**putative envelope protein ODV-E56-4, partial**
Mdem_scaffold_0845	23939	25738	K425_1614			putative alpha-amylase
Mdem_scaffold_0845	32624	32956	K425_1617			conserved uncharacterized protein
Mdem_scaffold_0845	74838	77274	K425_1616			conserved uncharacterized protein
Mdem_scaffold_0845	183748	186103	K425_1615			ankyrin-repeat domain-containing protein
**Mdem_scaffold_0919**	**1268**	**2626**	**K425_1685**	**odv-e66-17**		**putative envelope protein ODV-E66-17**
**Mdem_scaffold_0919**	**3526**	**5355**	**K425_1684**	**odv-e66-18**		**putative envelope protein ODV-E66-18**
**Mdem_scaffold_0919**	**8120**	**9577**	**K425_1686**	**odv-e66-19**		**putative envelope protein ODV-E66-19**
**Mdem_scaffold_0919**	**12921**	**13640**	**K425_1688**	**odv-e66-20**		**putative envelope protein ODV-E66-20, partial**
**Mdem_scaffold_0919**	**13977**	**15165**	**K425_1687**	**odv-e66-21**		**putative envelope protein ODV-E66-21**
Mdem_scaffold_0919	17045	20709	K425_1618			cytochrome b-561 domain containing protein
Mdem_scaffold_0919	31566	39431	K425_1619			t-complex protein 11 domain-containing protein
Mdem_scaffold_0919	36287	39993	K425_1683			regulator of telomere elongation helicase-like protein
Mdem_scaffold_0919	46260	49799	K425_1620			hypothetical protein
Mdem_scaffold_0919	50876	55896	K425_1621			hypothetical protein
Mdem_scaffold_0919	71118	72317	K425_1622			carboxylesterase domain-containing protein
Mdem_scaffold_0919	121582	122205	K425_1623			carboxylesterase domain-containing protein
Mdem_scaffold_0919	129692	130859	K425_1682			lipocalin-like protein
Mdem_scaffold_0919	158500	161599	K425_1624			retinol dehydrogenase-like protein
Mdem_scaffold_0919	165776	168080	K425_1625			venom allergen 5-like protein
Mdem_scaffold_0919	169595	172720	K425_1626			venom allergen 5-like protein
Mdem_scaffold_0919	173720	181001	K425_1681			calcium-binding mitochondrial carrier protein Aralar1-like protein
Mdem_scaffold_0919	207147	213143	K425_1627			serine protease stubble-like protein

Mdem_scaffold_0919	213255	226701	K425_1680			serpin-like protein
Mdem_scaffold_0919	227006	235014	K425_1628			ubiquitin protein ligase E3A-like protein
Mdem_scaffold_0919	235735	237628	K425_1679			crimpled-like protein
Mdem_scaffold_0919	238192	242793	K425_1678			NADH-cytochrome b5 reductase 2-like protein
Mdem_scaffold_0919	244593	253076	K425_1629			NADH-cytochrome b5 reductase 2-like protein
Mdem_scaffold_0919	253067	258669	K425_1677			T-complex protein 1, beta subunit
Mdem_scaffold_0919	263432	270538	K425_1630			inscuteable-like protein
Mdem_scaffold_0919	272017	274494	K425_1631			Bromodomain-containing protein
Mdem_scaffold_0919	274582	279021	K425_1676			hypothetical protein
Mdem_scaffold_0919	285449	289504	K425_1675			hypothetical protein
Mdem_scaffold_0919	290833	295870	K425_1632			ADP-ribosylation factor GTPase-activating protein 2-like protein
Mdem_scaffold_0919	297988	302936	K425_1674			conserved uncharacterized protein
Mdem_scaffold_0919	305186	322962	K425_1633			disintegrin and metalloproteinase-like protein
Mdem_scaffold_0919	327956	330653	K425_1673			hypothetical protein
Mdem_scaffold_0919	332074	340418	K425_1634			Kv channel-interacting protein 2-like protein
Mdem_scaffold_0919	342622	344663	K425_1672			alpha-tubulin-like protein
**Mdem_scaffold_0919**	**345232**	**347994**	**K425_1635**	**35a-8**	**Similar to Chelonus inanitus 35a**	**conserved uncharacterized protein 35a-like protein-8**
**Mdem_scaffold_0919**	**348137**	**350413**	**K425_1636**	**35a-9**	**Similar to Chelonus inanitus 35a**	**conserved uncharacterized protein 35a-like protein-9**
**Mdem_scaffold_0919**	**351060**	**352745**	**K425_1637**	**35a-10**	**Similar to Chelonus inanitus 35a**	**conserved uncharacterized protein 35a-like protein-10**
**Mdem_scaffold_0919**	**353152**	**354288**	**K425_1638**	**35a-11**	**Similar to Chelonus inanitus 35a**	**conserved uncharacterized protein 35a-like protein-11**
**Mdem_scaffold_0919**	**354662**	**355501**	**K425_1639**	**35a-12**	**Similar to Chelonus inanitus 35a**	**conserved uncharacterized protein 35a-like protein-12**
**Mdem_scaffold_0919**	**355743**	**360623**	**K425_1640**	**35a-13**	**Similar to Chelonus inanitus 35a**	**conserved uncharacterized protein 35a-like protein-13**
**Mdem_scaffold_0919**	**360960**	**363266**	**K425_1641**	**35a-14**	**Similar to Chelonus inanitus 35a**	**conserved uncharacterized protein 35a-like protein-14**
Mdem_scaffold_0919	368150	369771	K425_1671			7tm Odorant receptor domain-containing protein
Mdem_scaffold_0919	371718	377989	K425_1642			conserved uncharacterized protein
Mdem_scaffold_0919	383050	387089	K425_1643			conserved uncharacterized protein family 3
Mdem_scaffold_0919	390490	394354	K425_1670			king tubby-like protein
Mdem_scaffold_0919	395906	400102	K425_1669			fructose-bisphosphate aldolase-like protein
Mdem_scaffold_0919	404121	404903	K425_1668			fructose-bisphosphate aldolase-like protein
Mdem_scaffold_0919	405774	419350	K425_1644			ADP-ribosylation factor-like protein
Mdem_scaffold_0919	419639	425953	K425_1667			DNA-polymerase-delta
Mdem_scaffold_0919	426048	427639	K425_1666			conserved uncharacterized protein
Mdem_scaffold_0919	428541	432991	K425_1665			calumenin-like protein
Mdem_scaffold_0919	435714	439330	K425_1645			tetraspanin-like protein
Mdem_scaffold_0919	440326	450047	K425_1664			conserved uncharacterized protein
Mdem_scaffold_0919	464988	467975	K425_1646			alkylated DNA repair protein alkB 8-like protein
Mdem_scaffold_0919	469106	476426	K425_1663			conserved uncharacterized protein
Mdem_scaffold_0919	511818	515872	K425_1647			dopamine receptor-like protein
Mdem_scaffold_0919	520630	524719	K425_1648			PBP/GOBP family protein
Mdem_scaffold_0919	526352	527961	K425_1650			serine protease gastrulation-defective-like protein
Mdem_scaffold_0919	529175	530893	K425_1649			conserved uncharacterized protein
Mdem_scaffold_0919	535035	542037	K425_1662			PAX-interacting-like protein
Mdem_scaffold_0919	547059	570127	K425_1661			CNG channel-like protein
**Mdem_scaffold_0919**	**618516**	**620705**	**K425_1651**	**p74**	**synonym pif-0**	**per os infectivity factor, viral envelope protein p74**
Mdem_scaffold_0919	627737	632223	K425_1652			hypothetical protein
Mdem_scaffold_0919	628851	630191	K425_1660			BTB/POZ domain containing protein
Mdem_scaffold_0919	632755	634994	K425_1653		similar to Cotesia congregata Cc8L18.1 from p74 genomic region	conserved uncharacterized protein family 8
Mdem_scaffold_0919	635259	637223	K425_1654		similar to Cotesia congregata Cc8L18.1 from p74 genomic region	conserved uncharacterized protein family 8
Mdem_scaffold_0919	639264	642664	K425_1655			fidgetin-like protein
Mdem_scaffold_0919	642908	646166	K425_1656			chymotrypsin-2
Mdem_scaffold_0919	646020	647682	K425_1657			ankyrin-repeat domain-containing protein
Mdem_scaffold_0919	648230	652565	K425_1659			putative Ufm1-specific protease 2-like protein
Mdem_scaffold_0919	653279	659975	K425_1658			Spt20-like protein
Mdem_scaffold_0938	23773	23847	K425_1689			tRNA-Met
Mdem_scaffold_0938	25856	28208	K425_1691			RWD domain-containing protein
Mdem_scaffold_0938	31460	42857	K425_1744			NADP-dependent malic enzyme-like protein
Mdem_scaffold_0938	54075	55507	K425_1692			Phosphatidylinositol-specific phospholipase C, X domain-like protein
Mdem_scaffold_0938	59068	61958	K425_1693			Phosphatidylinositol N-acetylglucosaminyltransferase subunit C
Mdem_scaffold_0938	60963	62419	K425_1743			globin domain-containing protein
Mdem_scaffold_0938	65648	68434	K425_1694			conserved uncharacterized protein
Mdem_scaffold_0938	70663	72441	K425_1742			7tm Odorant receptor domain-containing protein
Mdem_scaffold_0938	75769	82049	K425_1741			eukaryotic translation initiation factor 2-alpha kinase-like protein
Mdem_scaffold_0938	86598	94355	K425_1740			bric-a-brac-like protein
Mdem_scaffold_0938	87685	88584	K425_1695			eukaryotic translation initiation factor 2-alpha kinase-like protein
Mdem_scaffold_0938	96880	98614	K425_1696			globin domain-containing protein
Mdem_scaffold_0938	103415	112806	K425_1697			flare-like protein
Mdem_scaffold_0938	119114	124468	K425_1698			slit-like protein
Mdem_scaffold_0938	126646	133846	K425_1739			rhythmically expressed 5-like protein
Mdem_scaffold_0938	151871	155150	K425_1738			eukaryotic initiation factor 4E-like protein
Mdem_scaffold_0938	156800	159589	K425_1699			Bromodomain-containing protein
Mdem_scaffold_0938	165889	178078	K425_1737			ebony-like protein
Mdem_scaffold_0938	196731	204637	K425_1745			conserved uncharacterized protein
Mdem_scaffold_0938	224428	224949	K425_1700			Thyrotropin-releasing hormone receptor-like protein
Mdem_scaffold_0938	245302	245994	K425_1701			Thyrotropin-releasing hormone receptor
Mdem_scaffold_0938	254487	260138	K425_1736			protein FAM45-like protein
Mdem_scaffold_0938	261120	267691	K425_1702			eukaryotic initiation factor A-like protein
Mdem_scaffold_0938	268705	273752	K425_1703			neprilysin-like protein
Mdem_scaffold_0938	274663	281257	K425_1735			Phosphatidylinositol 4-kinase-like protein
Mdem_scaffold_0938	341219	341311	K425_1690			tRNA-Ile
Mdem_scaffold_0938	464703	467188	K425_1734			conserved uncharacterized protein
Mdem_scaffold_0938	531396	533582	K425_1704			U2 small nuclear riboprotein auxiliary factor 50-like protein

Mdem_scaffold_0938	583034	590357	K425_1705			conserved uncharacterized protein
Mdem_scaffold_0938	615568	617220	K425_1706			Ecdysteroid kinase domain-containing protein
Mdem_scaffold_0938	617437	620524	K425_1707			Ecdysteroid kinase domain-containing protein
Mdem_scaffold_0938	662538	673030	K425_1708			Zona pellucida-like domain-containing protein
Mdem_scaffold_0938	681217	696257	K425_1733			Zona pellucida-like domain-containing protein
Mdem_scaffold_0938	699086	700646	K425_1732			NPC2-like protein
Mdem_scaffold_0938	705962	717333	K425_1731			WD repeat-containing protein
Mdem_scaffold_0938	718244	735163	K425_1709			Fmr1-like protein
Mdem_scaffold_0938	740470	741188	K425_1710			hypothetical protein
**Mdem_scaffold_0938**	**741776**	**744812**	**K425_1711**	**p47**		**viral RNA polymerase subunit p47**
Mdem_scaffold_0938	745432	748402	K425_1712			conserved uncharacterized protein family 12
Mdem_scaffold_0938	756290	757471	K425_1713			conserved uncharacterized protein family 12
Mdem_scaffold_0938	759188	760680	K425_1714			conserved uncharacterized protein family 12
Mdem_scaffold_0938	761747	764805	K425_1715			transmembrane protein 208-like protein
Mdem_scaffold_0938	765928	774209	K425_1717			nose resistant to fluoxetine protein 6-like protein
Mdem_scaffold_0938	775322	779787	K425_1716		similar to GFP_L6_0170	dehydrogenase E1 and transketolase domain-containing protein
Mdem_scaffold_0938	779801	784147	K425_1730			disintegrin and metalloproteinase-like protein
Mdem_scaffold_0938	784670	786238	K425_1718		similar to GIP_L3_0210 and GFP_L6_0160	chymotrypsin-like protein
Mdem_scaffold_0938	821383	825001	K425_1719			MFS sugar transporter domain-containing protein
Mdem_scaffold_0938	833499	835358	K425_1720			7tm Odorant receptor domain-containing protein
Mdem_scaffold_0938	836537	838600	K425_1721			hypothetical protein
Mdem_scaffold_0938	838390	840750	K425_1729			globin domain-containing protein
Mdem_scaffold_0938	858329	865235	K425_1728		similar to GFP_L6_0020 and GIP_L3_0040	phospholipase B-like protein
Mdem_scaffold_0938	882613	890513	K425_1722			methionine adenosyltransferase
Mdem_scaffold_0938	890924	907615	K425_1727			Zinc-finger C2H2 domain and EB module-containing protein
Mdem_scaffold_0938	910549	915184	K425_1723			conserved uncharacterized protein
Mdem_scaffold_0938	916012	924067	K425_1726			ubiquitin-protein ligase HERC4-like protein
Mdem_scaffold_0938	925354	932934	K425_1724			PHD finger-like protein
Mdem_scaffold_0938	932069	934020	K425_1725			methionyl-tRNA formyltransferase
Mdem_scaffold_1154	30	1946	K425_6			putative ataxin-3 protein
Mdem_scaffold_1154	3188	8654	K425_7			negative elongation factor E
Mdem_scaffold_1154	3222	11147	K425_139			conserved uncharacterized protein
Mdem_scaffold_1154	13001	18696	K425_138			protein SCAI-like protein
Mdem_scaffold_1154	47307	48445	K425_8			DNA-binding protein D-ETS-3-like protein
Mdem_scaffold_1154	53659	56126	K425_136			N-acetylgalactosamine kinase-like protein
Mdem_scaffold_1154	56941	65177	K425_137			ankyrin-repeat domain-containing protein
Mdem_scaffold_1154	99503	115321	K425_135			conserved uncharacterized protein
Mdem_scaffold_1154	135817	159205	K425_9			homeobox protein caupolican-like protein
Mdem_scaffold_1154	187709	200663	K425_10			homeobox protein caupolican-like protein
Mdem_scaffold_1154	237873	241259	K425_134			neprilysin-like protein
Mdem_scaffold_1154	290456	292191	K425_133			conserved uncharacterized protein
Mdem_scaffold_1154	293552	298197	K425_11			fatty acid-binding protein
**Mdem_scaffold_1154**	**298813**	**299357**	**K425_12**		**similar to vlf-1b-1**	**conserved hypothetical protein**
Mdem_scaffold_1154	309615	317415	K425_13			serine protease-like protein
Mdem_scaffold_1154	317669	320305	K425_132			splicing factor 3A subunit 3-like protein
Mdem_scaffold_1154	319883	322672	K425_14			origin recognition complex subunit 1-like protein
**Mdem_scaffold_1154**	**323090**	**325417**	**K425_131**	**odv-e66-3**		**putative envelope protein ODV-E66-3**
Mdem_scaffold_1154	402622	428524	K425_15			transcription factor AP-2-like protein
Mdem_scaffold_1154	432612	434622	K425_130			ubiquitin carboxy-terminal hydrolase-like protein
Mdem_scaffold_1154	436290	440483	K425_129			conserved uncharacterized protein
Mdem_scaffold_1154	474298	474851	K425_128			conserved uncharacterized protein
Mdem_scaffold_1154	482240	487353	K425_16			guanylate kinase domain-containing protein
Mdem_scaffold_1154	490626	495034	K425_17			ankyrin-repeat domain-containing protein
Mdem_scaffold_1154	494301	497561	K425_127			dnaJ domain-containing protein
Mdem_scaffold_1154	498545	500495	K425_18			u2 small nuclear ribonucleoprotein auxiliary factor 35 kDa subunit-related protein 1-like protein
Mdem_scaffold_1154	501359	503811	K425_126			alpha-tocopherol transfer-like protein
Mdem_scaffold_1154	511539	517661	K425_125			SAPS domain-containing protein
Mdem_scaffold_1154	518318	521355	K425_19			bicoid stability factor-like protein
Mdem_scaffold_1154	521919	526898	K425_20			bicoid stability factor-like protein
Mdem_scaffold_1154	526869	527783	K425_124			barrier-to-autointegration factor A-like protein
Mdem_scaffold_1154	527784	529454	K425_21			NEFA-interacting nuclear protein NIP30-like protein
Mdem_scaffold_1154	529350	532490	K425_123			Sly1-like protein
Mdem_scaffold_1154	534020	539101	K425_122			conserved uncharacterized protein
Mdem_scaffold_1154	547299	549660	K425_22			d-2-hydroxyglutarate dehydrogenase, mitochondrial-like protein
Mdem_scaffold_1154	551457	553154	K425_23			conserved uncharacterized protein
Mdem_scaffold_1154	563592	567265	K425_24			conserved uncharacterized protein
Mdem_scaffold_1154	573194	577043	K425_121			cdc42-like protein
Mdem_scaffold_1154	577136	578307	K425_25			proteasome subunit beta type-1-like protein
Mdem_scaffold_1154	578493	579969	K425_120			ribosomal protein S7
Mdem_scaffold_1154	580267	581857	K425_26			cytochrome c oxidase subunit IV-like protein
Mdem_scaffold_1154	584681	588788	K425_119			PAP-associated domain-containing protein
Mdem_scaffold_1154	589257	590560	K425_28			cytochrome c oxidase subunit Vb-like protein
Mdem_scaffold_1154	590561	592971	K425_118			peroxin 13-like protein
Mdem_scaffold_1154	593849	595006	K425_27			PKC-activated protein phosphatase-1 inhibitor-like protein
Mdem_scaffold_1154	594302	600778	K425_117			SART-1 domain-containing protein
Mdem_scaffold_1154	604480	610285	K425_116			decapentaplegic-like protein
Mdem_scaffold_1154	615887	619264	K425_115			AMP-binding domain-containing protein
Mdem_scaffold_1154	619616	620461	K425_29			Splicing factor 3B subunit 10 (SF3b10)-like protein
Mdem_scaffold_1154	620452	621955	K425_114			exosome complex component MTR3-like protein
Mdem_scaffold_1154	621956	625246	K425_31			glucose dehydrogenase acceptor-like protein

Mdem_scaffold_1154	624252	625710	K425_113			RNA recognition motif domain-containing protein
Mdem_scaffold_1154	626003	629593	K425_30			grapes-like protein
Mdem_scaffold_1154	629975	631852	K425_112			nuclear inhibitor of protein phosphatase 1-like protein
Mdem_scaffold_1154	631930	636579	K425_32			Blastoderm-specific 25D-like protein
Mdem_scaffold_1154	637451	639273	K425_111			endothelial differentiation-related factor 1-like protein
Mdem_scaffold_1154	640278	643166	K425_110			conserved uncharacterized protein
Mdem_scaffold_1154	643707	645952	K425_109			conserved uncharacterized protein
Mdem_scaffold_1154	650250	652692	K425_33			proton-coupled amino acid transporter 4-like protein
Mdem_scaffold_1154	656204	660185	K425_34			proton-coupled amino acid transporter 4-like protein
Mdem_scaffold_1154	660198	661383	K425_108			RNA polymerase II subunit 10
Mdem_scaffold_1154	661180	665447	K425_35			PAB-dependent poly(A)-specific ribonuclease subunit-like protein
Mdem_scaffold_1154	669207	672178	K425_36			conserved uncharacterized protein
Mdem_scaffold_1154	673930	675521	K425_107			conserved uncharacterized protein
Mdem_scaffold_1154	676062	677861	K425_37			conserved uncharacterized protein
Mdem_scaffold_1154	678272	681725	K425_38			flotillin-1-like protein
Mdem_scaffold_1154	679665	683455	K425_106			conserved uncharacterized protein
Mdem_scaffold_1154	683020	687134	K425_39			hypothetical protein
Mdem_scaffold_1154	687999	695309	K425_40			nervous wreck-like protein
Mdem_scaffold_1154	696325	698315	K425_105			3-hydroxyacyl-CoA dehydrogenase type-2-like protein
Mdem_scaffold_1154	698621	706177	K425_41			cytochrome P450 domain-containing protein
Mdem_scaffold_1154	706666	711259	K425_104			phospholipase D3-like protein
Mdem_scaffold_1154	890122	899887	K425_42			conserved uncharacterized protein
Mdem_scaffold_1154	905247	914193	K425_103			Rab-GTPase-TBC domain-containing protein
Mdem_scaffold_1154	932349	935981	K425_102			kinesin-like protein KIF18A
Mdem_scaffold_1154	938122	939676	K425_43			hypothetical protein
Mdem_scaffold_1154	940038	942121	K425_44			hypothetical protein
Mdem_scaffold_1154	942122	942837	K425_101			hypothetical protein
Mdem_scaffold_1154	943316	947057	K425_45			xenotropic and polytropic retrovirus receptor 1-like protein
Mdem_scaffold_1154	949154	963761	K425_100			Ion channel regulatory protein UNC-93 domain-containing protein
Mdem_scaffold_1154	978997	983885	K425_46			DNA mismatch repair protein mutL-like protein
Mdem_scaffold_1154	983560	985987	K425_99			negative elongation factor D-like protein
Mdem_scaffold_1154	987780	994054	K425_47			carnitine O-palmitoyltransferase 1-like protein
Mdem_scaffold_1154	993937	995583	K425_98			conserved uncharacterized protein
Mdem_scaffold_1154	996546	999378	K425_48			T-complex protein 1, delta subunit
Mdem_scaffold_1154	999801	1003053	K425_97			N-acetylgalactosaminyltransferase 7-like protein
Mdem_scaffold_1154	1002377	1006202	K425_49			THAP domain-containing protein
Mdem_scaffold_1154	1007260	1008337	K425_50			7tm Odorant receptor domain-containing protein
Mdem_scaffold_1154	1010278	1011246	K425_51			7tm Odorant receptor domain-containing protein
Mdem_scaffold_1154	1013265	1018587	K425_52			7tm Odorant receptor domain-containing protein
Mdem_scaffold_1154	1021184	1025824	K425_53			7tm Odorant receptor domain-containing protein
Mdem_scaffold_1154	1026722	1028877	K425_54			7tm Odorant receptor domain-containing protein
Mdem_scaffold_1154	1030363	1030713	K425_55			7tm Odorant receptor domain-containing protein
Mdem_scaffold_1154	1031689	1032368	K425_56			7tm Odorant receptor domain-containing protein
Mdem_scaffold_1154	1034274	1036078	K425_57			7tm Odorant receptor domain-containing protein
Mdem_scaffold_1154	1036572	1043333	K425_58			7tm Odorant receptor domain-containing protein
Mdem_scaffold_1154	1044763	1048943	K425_59			7tm Odorant receptor domain-containing protein
Mdem_scaffold_1154	1049711	1051532	K425_60			conserved uncharacterized protein
Mdem_scaffold_1154	1053282	1058756	K425_61			7tm Odorant receptor domain-containing protein
Mdem_scaffold_1154	1059795	1061580	K425_96			Pacifastin inhibitor (LCMII) domain-containing protein
Mdem_scaffold_1154	1063344	1066684	K425_62			Citrate transporter domain-containing protein
Mdem_scaffold_1154	1092739	1102542	K425_63			scavenger receptor class B member-like protein
Mdem_scaffold_1154	1104725	1107163	K425_64			Low-density lipoprotein receptor -like protein
Mdem_scaffold_1154	1109076	1116548	K425_95			striatin-like protein
Mdem_scaffold_1154	1120115	1125222	K425_65			epithelial splicing regulatory-like protein
Mdem_scaffold_1154	1126886	1132735	K425_66			peroxidasin-like protein
Mdem_scaffold_1154	1132945	1134587	K425_94			acyl-coenzyme A thioesterase-like protein
Mdem_scaffold_1154	1134648	1139146	K425_93			diphosphomevalonate decarboxylase
Mdem_scaffold_1154	1136672	1139158	K425_67			actin domain-containing protein
Mdem_scaffold_1154	1140864	1144363	K425_68			cleavage and polyadenylation specificity factor subunit 5-like protein
Mdem_scaffold_1154	1144878	1148871	K425_69			conserved uncharacterized protein
Mdem_scaffold_1154	1149038	1151650	K425_92			phytanoyl-CoA dioxygenase domain-containing protein
Mdem_scaffold_1154	1152969	1158076	K425_70			conserved uncharacterized protein
Mdem_scaffold_1154	1215576	1217483	K425_91			ubiquitin-related modifier 1-like protein
Mdem_scaffold_1154	1220443	1225189	K425_90			lung seven transmembrane receptor domain-containing protein
Mdem_scaffold_1154	1225262	1227505	K425_89			syntaxin-17-like protein
Mdem_scaffold_1154	1234500	1236992	K425_88			putative dehydrogenase and reductase
Mdem_scaffold_1154	1249926	1251494	K425_87			hypothetical protein
Mdem_scaffold_1154	1262890	1265961	K425_86			Insect allergen related repeat, nitrile-specifier detoxification domain-containing protein
Mdem_scaffold_1154	1293091	1300165	K425_85			Zinc finger, C2H2 type domain-containing protein
Mdem_scaffold_1154	1457877	1464966	K425_84			shroom-like protein
Mdem_scaffold_1154	1484663	1489616	K425_83			hypothetical protein
Mdem_scaffold_1154	1546657	1549459	K425_71			putative nucleolar GTP-binding protein
Mdem_scaffold_1154	1553273	1557301	K425_72			coronin-like protein
Mdem_scaffold_1154	1562091	1564595	K425_73			lipopolysaccharide-induced tumor necrosis factor-alpha factor-like protein
Mdem_scaffold_1154	1565549	1567586	K425_75			lipopolysaccharide-induced tumor necrosis factor-alpha factor-like protein
Mdem_scaffold_1154	1566352	1569447	K425_74			lipopolysaccharide-induced tumor necrosis factor-alpha factor-like protein
Mdem_scaffold_1154	1570228	1573552	K425_76			Histidyl-tRNA synthetase, cytoplasmic
Mdem_scaffold_1154	1572103	1582185	K425_82			Histidyl-tRNA synthetase, cytoplasmic
Mdem_scaffold_1154	1586069	1590536	K425_81			hypothetical protein

Mdem_scaffold_1154	1591807	1596614	K425_80			EB module-containing protein
Mdem_scaffold_1154	1597939	1599508	K425_77			hypothetical protein
Mdem_scaffold_1154	1599509	1601843	K425_78			conserved uncharacterized protein
Mdem_scaffold_1154	1601226	1606666	K425_79			Immunoglobulin domain-containing protein
Mdem_scaffold_1199	29604	35477	K425_181			Transcription factor castor-like protein
Mdem_scaffold_1199	61117	71047	K425_182			conserved uncharacterized protein
Mdem_scaffold_1199	72407	74653	K425_183		similar to 19.5g1 protein from Chelonus inanitus bracovirus	conserved uncharacterized protein
Mdem_scaffold_1199	75617	90529	K425_187			alkaline phosphatase, tissue-nonspecific isozyme-like protein
Mdem_scaffold_1199	95469	97370	K425_186			hypothetical protein
Mdem_scaffold_1199	114660	119705	K425_184		similar to GIP_L6_0020, GFP_L3_0430	G-patch domain-contaning protein
Mdem_scaffold_1199	118077	119162	K425_185			hypothetical protein
Mdem_scaffold_1213	1018	2634	K425_190		similar to GFP_L5_0170	conserved uncharacterized protein family 1
Mdem_scaffold_1213	16930	18907	K425_189			transposable element-like protein
**Mdem_scaffold_1213**	**26279**	**28159**	**K425_188**	**vlf-1b-1**	**synonym HzNVorf140-like, present in Microplitis demolitor virus particles**	**very late factor 1b-1, bracovirus particle protein**
Mdem_scaffold_1462	142	6458	K425_247			Microtubule-binding protein MIP-T3-like protein
Mdem_scaffold_1462	11050	26452	K425_210			protein Malvolio-like protein
Mdem_scaffold_1462	28722	35431	K425_245			prostatic acid phosphatase-like protein
Mdem_scaffold_1462	38283	41763	K425_244			conserved uncharacterized protein
Mdem_scaffold_1462	47378	51827	K425_211			hypothetical protein
Mdem_scaffold_1462	56804	57079	K425_246			histone H4 replacement-like protein
Mdem_scaffold_1462	69982	70559	K425_243			histone H2A-like protein
Mdem_scaffold_1462	70593	71198	K425_212			histone H2B-like protein
Mdem_scaffold_1462	75163	94006	K425_242		similar to GIP_L8_0090 and GIP_L8_0100	NAD nucleotidase
Mdem_scaffold_1462	99028	102378	K425_213		similar to GIP_L8_0090 and GIP_L8_0100	5' nucleotidase, putative
Mdem_scaffold_1462	104405	118625	K425_214		similar to GIP_L8_0060	conserved uncharacterized protein
Mdem_scaffold_1462	127011	129178	K425_215			hypothetical protein
Mdem_scaffold_1462	130282	156201	K425_241		similar to GIP_L8_0050	P-type ATPase of unknown pump specificity (type V)
Mdem_scaffold_1462	166508	177083	K425_240		similar to GFP_L7_0800 and GIP_L8_0040	hyaluronidase-like protein
Mdem_scaffold_1462	182279	183536	K425_239		similar to GIP_L1_00710, GFP_L7_0750 and GFP_L7_0760	hyaluronidase
Mdem_scaffold_1462	186733	189019	K425_238		similar to GIP_L1_00710, GFP_L7_0750 and GFP_L7_0760	hyaluronidase
Mdem_scaffold_1462	191489	199383	K425_237		similar to GFP_L7_0740 and GIP_L1_00700	N-myristoyltransferase, putative
Mdem_scaffold_1462	201614	203551	K425_236		similar to GFP_L7_0730 and GIP_L1_00690	conserved uncharacterized protein
Mdem_scaffold_1462	203940	204011	K425_209			tRNA-Ser
**Mdem_scaffold_1462**	**207985**	**208806**	**K425_216**	**orph-P1**		**conserved hypothetical protein**
**Mdem_scaffold_1462**	**217931**	**218686**	**K425_217**	**orph-K1-1**		**conserved hypothetical protein**
**Mdem_scaffold_1462**	**220695**	**222273**	**K425_235**	**orph-K1-2**		**conserved hypothetical protein**
**Mdem_scaffold_1462**	**223842**	**224575**	**K425_218**	**orph-K1-3**	**similar to orph-K1**	**hypothetical protein**
**Mdem_scaffold_1462**	**225913**	**230837**	**K425_234**	**orph-K1-4**		**hypothetical protein**
**Mdem_scaffold_1462**	**233190**	**238900**	**K425_219**	**orph-K2**		**hypothetical protein**
**Mdem_scaffold_1462**	**237108**	**240824**	**K425_233**	**orph-K3**		**hypothetical protein**
**Mdem_scaffold_1462**	**240720**	**243546**	**K425_232**	**orph-K4**		**hypothetical protein**
**Mdem_scaffold_1462**	**240801**	**241109**	**K425_220**	**orph-K1**		**hypothetical protein**
**Mdem_scaffold_1462**	**245225**	**245491**	**K425_221**	**orph-Q1**	**similar to orph-M3**	**conserved hypothetical protein**
**Mdem_scaffold_1462**	**245737**	**246360**	**K425_222**	**orph-Q2**		**conserved hypothetical protein**
**Mdem_scaffold_1462**	**248051**	**250525**	**K425_231**	**orph-Q3**		**conserved hypothetical protein**
**Mdem_scaffold_1462**	**250590**	**251467**	**K425_223**	**orph-Q4**		**conserved hypothetical protein Segment Q family**
**Mdem_scaffold_1462**	**252823**	**254053**	**K425_224**	**orph-Q5**		**conserved hypothetical protein Segment Q family**
**Mdem_scaffold_1462**	**255114**	**257123**	**K425_230**	**orph-Q6**		**conserved hypothetical protein**
**Mdem_scaffold_1462**	**257659**	**258244**	**K425_229**	**orph-Q7**		**conserved hypothetical protein**
**Mdem_scaffold_1462**	**259642**	**260432**	**K425_225**	**orph-D5**		**conserved hypothetical protein**
**Mdem_scaffold_1462**	**260469**	**261202**	**K425_226**	**orph-D6**		**conserved hypothetical protein**
**Mdem_scaffold_1462**	**263057**	**265266**	**K425_228**	**orph-D7**		**conserved hypothetical protein**
**Mdem_scaffold_1462**	**264392**	**265428**	**K425_227**	**orph-D8**		**conserved hypothetical protein**
**Mdem_scaffold_1559**	**1816**	**4332**	**K425_415**	**odv-e66-5**		**putative envelope protein ODV-E66-5**
**Mdem_scaffold_1559**	**5445**	**7426**	**K425_410**	**odv-e66-6**		**putative envelope protein ODV-E66-6**
**Mdem_scaffold_1559**	**8344**	**11093**	**K425_411**	**odv-e66-7**		**putative envelope protein ODV-E66-7**
Mdem_scaffold_1559	12935	15858	K425_412		similar to GIP_L7_0080, GFP_L5_0240, GIP_L2_0090, GFP_L5_0230, GFP_L5_0070, GFP_L5_0090, GIP_L2_0100, GIP_L7_0040, GIP_L7_0010, and GFP_L5_0190	conserved uncharacterized protein family 7
**Mdem_scaffold_1559**	**17598**	**20694**	**K425_414**	**odv-e66-8**		**putative envelope protein ODV-E66-8, partial**
Mdem_scaffold_1559	31649	43292	K425_413			Homeobox protein cut
**Mdem_scaffold_1669**	**58753**	**60347**	**K425_475**		**similar to 30b from Chelonus inanitus and Cotesia congregata hypothetical protein CBL47423**	**conserved uncharacterized protein**
Mdem_scaffold_1798	2919	3935	K425_519			RNA-binding protein MEX3B-like protein
Mdem_scaffold_1798	28732	30994	K425_518			RNA-binding protein MEX3B-like protein
Mdem_scaffold_1798	38067	40091	K425_476			Protein tyrosine phosphatase-like protein ptplad1
Mdem_scaffold_1798	40104	42586	K425_477			aldehyde dehydrogenase, mitochondrial-like protein
Mdem_scaffold_1798	42844	45926	K425_517			Myotubularin-associated-like protein
Mdem_scaffold_1798	46207	49593	K425_478			Meiotic recombination protein REC8-like protein
Mdem_scaffold_1798	49818	51296	K425_516			hypothetical protein

Mdem_scaffold_1798	51611	55330	K425_479			ATP-dependent protease La
Mdem_scaffold_1798	55405	56357	K425_515			gonadal protein gdl-like protein
Mdem_scaffold_1798	56358	57580	K425_480			28S ribosomal protein S15, mitochondrial-like protein
Mdem_scaffold_1798	57630	59348	K425_514			Apoptosis antagonizing transcription factor domain-containing protein
Mdem_scaffold_1798	62069	69334	K425_481			fantom-like protein
Mdem_scaffold_1798	64828	66854	K425_513			F-actin capping protein alpha subunit-like protein
Mdem_scaffold_1798	72214	74186	K425_512			hypothetical protein
Mdem_scaffold_1798	81045	109011	K425_482			Muscle M-line assembly protein unc-89-like protein
Mdem_scaffold_1798	83307	83828	K425_511			hypothetical protein
Mdem_scaffold_1798	109320	110973	K425_510			NEDD8-conjugating enzyme UBE2F-like protein
Mdem_scaffold_1798	110134	112786	K425_483			vacuolar protein-sorting-associated protein 25-like protein
Mdem_scaffold_1798	112086	117690	K425_509			excitatory amino acid transporter 4-like protein
Mdem_scaffold_1798	121028	122385	K425_508			Cyclin-dependent kinase subunit 30A
Mdem_scaffold_1798	122467	130324	K425_484			UDP-N-acetylglucosamine-dolichyl-phosphate N-acetylglucosaminephosphotransferase-like protein
Mdem_scaffold_1798	123740	126311	K425_507			beta-1,4-mannosyl-glycoprotein 4-beta-N-acetylglucosaminyltransferase-like protein
Mdem_scaffold_1798	127828	130928	K425_506			mitochondrial import receptor subunit TOM70-like protein
Mdem_scaffold_1798	130558	132523	K425_485			NuA4, Histone acetyltransferase subunit NuA4 domain-containing protein
Mdem_scaffold_1798	132826	133905	K425_505			Insect cuticle domain-containing protein
Mdem_scaffold_1798	134350	137545	K425_486			gonadotropin-releasing hormone receptor-like protein
Mdem_scaffold_1798	138401	144648	K425_487			thrombospondin-like protein
Mdem_scaffold_1798	145788	156029	K425_504			c-Maf-inducing-like protein
Mdem_scaffold_1798	159491	166608	K425_503			Metallopeptidase family M24 protein
Mdem_scaffold_1798	170198	171151	K425_502			Glycogen synthase kinase-3 binding domain-containing protein
Mdem_scaffold_1798	171337	175599	K425_501			HAUS augmin-like protein complex subunit 3-like protein
Mdem_scaffold_1798	178132	180631	K425_500			Immunoglobulin domain-containing protein
Mdem_scaffold_1798	277674	294741	K425_488			Immunoglobulin domain-containing protein
**Mdem_scaffold_1798**	**306628**	**309224**	**K425_499**		**present in Microplitis demolitor virus particles**	**putative viral envelope protein VP91**
Mdem_scaffold_1798	311685	320864	K425_489			gonadotropin-releasing hormone receptor-like protein
Mdem_scaffold_1798	317759	318903	K425_496			hypothetical protein
Mdem_scaffold_1798	322759	323903	K425_497			hypothetical protein
Mdem_scaffold_1798	325930	329706	K425_498			conserved uncharacterized protein
Mdem_scaffold_1798	333705	336414	K425_495			conserved uncharacterized protein
Mdem_scaffold_1798	338270	340870	K425_494			cactin-like protein
Mdem_scaffold_1798	341209	343114	K425_490			2-oxoisovalerate dehydrogenase subunit alpha, mitochondrial-like protein
Mdem_scaffold_1798	343115	344039	K425_493			mitochondrial ribosomal protein L17, isoform A
Mdem_scaffold_1798	344239	345862	K425_491			loquacious-like protein
Mdem_scaffold_1798	346404	362649	K425_492			putative dynein heavy chain 5, axonemal-like protein
Mdem_scaffold_1887	12001	23176	K425_520			hypothetical protein
Mdem_scaffold_1887	25068	26177	K425_521			hypothetical protein
Mdem_scaffold_1887	36855	38594	K425_522			putative transposase
Mdem_scaffold_1887	40268	41090	K425_525			conserved uncharacterized protein
**Mdem_scaffold_1887**	**59162**	**60302**	**K425_524**		**similar to vlf-1**	**reverse transcriptase domain-containing protein**
**Mdem_scaffold_1887**	**70283**	**72349**	**K425_523**	**vlf-1b-2**	**synonym HzNVorf140-like, present in Microplitis demolitor virus particles**	**very late factor 1b-2, bracovirus particle protein**
Mdem_scaffold_2026	4368	12524	K425_560			conserved uncharacterized protein
Mdem_scaffold_2026	19587	23526	K425_559			conserved uncharacterized protein
**Mdem_scaffold_2026**	**24788**	**26162**	**K425_555**	**ac92-like-1**	**similar to HzNVorf13**	**ac92-like protein, putative sulfhydryl oxidase-1**
Mdem_scaffold_2026	30620	31180	K425_556			conserved uncharacterized protein
Mdem_scaffold_2026	31572	34637	K425_558			hypothetical protein
Mdem_scaffold_2026	49392	50018	K425_557			conserved uncharacterized protein
Mdem_scaffold_2026	49580	49677	K425_554			tRNA-seC
**Mdem_scaffold_3298**	**954**	**2437**	**K425_948**	**orph-D9**		**conserved hypothetical protein**
**Mdem_scaffold_3298**	**4992**	**9649**	**K425_947**	**orph-B1**		**conserved hypothetical protein family 5, BEN domain-containing**
**Mdem_scaffold_3298**	**11966**	**18742**	**K425_945**	**orph-A3**	**similar to orph-B1**	**conserved hypothetical protein family 5, BEN domain-containing**
**Mdem_scaffold_3298**	**21324**	**22288**	**K425_946**	**orph-A4**		**conserved hypothetical protein**
**Mdem_scaffold_3298**	**23124**	**23642**	**K425_944**	**orph-A1**		**conserved hypothetical protein**
**Mdem_scaffold_3298**	**23788**	**24165**	**K425_943**	**orph-A2**		**conserved hypothetical protein**
**Mdem_scaffold_3298**	**29198**	**30296**	**K425_942**	**orph-L1**		**conserved hypothetical protein**
**Mdem_scaffold_3298**	**38097**	**39571**	**K425_940**	**orph-L4**		**hypothetical protein**
**Mdem_scaffold_3298**	**36359**	**37460**	**K425_941**	**orph-L3**		**hypothetical protein**
**Mdem_scaffold_3298**	**44562**	**44916**	**K425_916**	**orph-F2**		**hypothetical protein**
**Mdem_scaffold_3298**	**45398**	**45901**	**K425_939**	**ank-F5**		**viral ankyrin**
**Mdem_scaffold_3298**	**46180**	**46674**	**K425_938**	**ank-F4**		**viral ankyrin**
**Mdem_scaffold_3298**	**48028**	**48393**	**K425_917**	**orph-F3**		**hypothetical protein**
**Mdem_scaffold_3298**	**48681**	**49913**	**K425_918**	**orph-F4**		**conserved hypothetical protein**
**Mdem_scaffold_3298**	**50911**	**51318**	**K425_937**	**orph-F1**		**conserved hypothetical protein**
**Mdem_scaffold_3298**	**52329**	**54091**	**K425_936**	**orph-F5**		**hypothetical protein**
**Mdem_scaffold_3298**	**54754**	**55109**	**K425_919**	**orph-I2**		**hypothetical protein**
**Mdem_scaffold_3298**	**56545**	**56904**	**K425_920**	**orph-I3**		**hypothetical protein**
**Mdem_scaffold_3298**	**57350**	**57853**	**K425_935**	**ank-I1**		**viral ankyrin**
**Mdem_scaffold_3298**	**61373**	**64088**	**K425_921**	**orph-I4**		**conserved hypothetical protein**
**Mdem_scaffold_3298**	**66378**	**66707**	**K425_934**	**orph-I1**		**conserved hypothetical protein**
**Mdem_scaffold_3298**	**70169**	**70501**	**K425_922**	**orph-M3**		**conserved hypothetical protein**
**Mdem_scaffold_3298**	**70749**	**71066**	**K425_923**	**orph-M2**		**conserved hypothetical protein**
**Mdem_scaffold_3298**	**73227**	**73562**	**K425_933**	**orph-M1**		**conserved hypothetical protein**
**Mdem_scaffold_3298**	**74907**	**75940**	**K425_924**	**orph-M6**		**conserved hypothetical protein**
**Mdem_scaffold_3298**	**77934**	**78899**	**K425_932**	**orph-M5**		**conserved hypothetical protein**
**Mdem_scaffold_3298**	**82328**	**82660**	**K425_931**	**orph-M4**		**conserved hypothetical protein**

**Mdem_scaffold_3298**	**82915**	**83274**	**K425_930**	**orph-M7**		**conserved hypothetical protein**
**Mdem_scaffold_3298**	**89617**	**90123**	**K425_929**	**ank-G4**	**synonym IkB-G2**	**viral ankyrin**
**Mdem_scaffold_3298**	**90432**	**90962**	**K425_928**	**ank-G3**	**synonym IkB-G1**	**viral ankyrin**
**Mdem_scaffold_3298**	**93653**	**94503**	**K425_925**	**orph-G3**		**conserved hypothetical protein**
**Mdem_scaffold_3298**	**95306**	**96211**	**K425_926**	**orph-G2**		**conserved hypothetical protein**
**Mdem_scaffold_3298**	**101085**	**103666**	**K425_927**	**glc1.8**		**Glc1.8**
Mdem_scaffold_3350	6409	8178	K425_975			hypothetical protein
Mdem_scaffold_3350	10030	11101	K425_965		similar to GIP_L2_0080	conserved hypothetical protein
Mdem_scaffold_3350	11136	12015	K425_966		similar to GIP_L2_0080	conserved hypothetical protein
Mdem_scaffold_3350	13388	13738	K425_967			sesquipedalian-1-like protein
**Mdem_scaffold_3350**	**15063**	**16649**	**K425_974**			**Egf-like protein**
**Mdem_scaffold_3350**	**16888**	**19044**	**K425_973**			**Glc-like protein**
**Mdem_scaffold_3350**	**21439**	**23377**	**K425_972**			**Egf-like protein**
Mdem_scaffold_3350	24255	24957	K425_971			hypothetical protein
Mdem_scaffold_3350	25647	26498	K425_970		weak similarity to Cc50C22.5	Egf-like protein
**Mdem_scaffold_3350**	**28694**	**29791**	**K425_969**			**conserved hypothetical protein**
Mdem_scaffold_3350	30278	32157	K425_968			conserved hypothetical protein
