# Supplementary material for: Widespread Genome Reorganization of an Obligate Virus Mutualist
Source: PLoS Genet. 2014 Sep 18;10(9):e1004660. doi: 10.1371/journal.pgen.1004660 (PMC4169385; doi:10.1371/journal.pgen.1004660)
Supplement: Table S2 — Genes or gene families shared between nudivirus-like and proviral segment regions of the genome. (PDF) [file pgen.1004660.s006.pdf]

Links between nudivirus-like regions and regions flanking proviral segments

BVs (M. demolitor specifically)

Scaffold	Segment-linked genes Md	Nudivirus-linked genes	Nearest separating distance	Note
Mdem_scaffold_0157	Segment T	HzNVorf93-like	5.4 kb	A scaffolds that contains Segment T also has a nudivirus-like gene

BVs

Scaffold	Segment-linked genes Md	Segment linked genes other BVs	Nudivirus-linked genes	Nearest separating distance	Note
Mdem_scaffold_0938	K425_1716, K425_1718, K425_1728	Glyptapanteles Locus 3 (Desjardins et al. 2008): GFP_L6_0020, GFP_L6_0170, and GFP_L6_0160; GIP_L3_0040 and GIP_L3_0210	p47	30.5 kb	Scaffold 938 possesses genes that are similar to those flanking Segments 22 and 23 in Glyptapanteles species, and also carries a nudivirus-like gene p47
Mdem_scaffold_1213	K425_190	Glyptapanteles flavicoxis Locus 7 (Desjardins et al. 2008): GFP_L5_0170	vlf-1b-1	23.6 kb	Scaffold 1213 has a gene similar to a gene flanking Segments 27 and 28 in Glyptapanteles flavicoxis, and also carries vlf-1b-1
Mdem_scaffold_0019	K425_529, K425_534, K425_544	Glyptapanteles flavicoxis Locus 7 (Desjardins et al. 2008): GFP_L5_0170 and GFP_L5_0200	int-2	24.2 kb	Scaffold 19 has possesses genes similar to those flanking Segments 27 and 28 in Glyptapanteles flavicoxis, and also carries int-2
Mdem_scaffold_0275	K425_820 (conserved uncharacterized protein family 7)	Glyptapanteles flavicoxis Locus 7 (Desjardins et al. 2008): GFP_L5_0070, GFP_L5_0090, GFP_L5_0190, GFP_L5_0230, GFP_L5_0240; Glyptapanteles indiensis Locus 6, Locus 7: GIP_L2_0090, GIP_L2_0100, GIP_L7_0040, GIP_L7_0010, GIP_L7_0080	several nudivirus-like genes, closest is helicase	58.5 kb	A conserved uncharacterized gene family member in *M. demolitor* is present in several *Glyptapanteles* loci containing viral segments (Segment 27 and 28 in Gf, Segment 26 in Gi) and is on a Md scaffold that contains several nudivirus-like genes
Mdem_scaffold_1559	K425_412 (conserved uncharacterized protein family 7)	Glyptapanteles flavicoxis Locus 7 (Desjardins et al. 2008): GFP_L5_0070, GFP_L5_0090, GFP_L5_0190, GFP_L5_0230, GFP_L5_0240; Glyptapanteles indiensis Locus 6, Locus 7: GIP_L2_0090, GIP_L2_0100, GIP_L7_0040, GIP_L7_0010, GIP_L7_0080	cluster of odv-e66 genes	1.7 kb	A conserved uncharacterized gene family member in *M. demolitor* is present in several *Glyptapanteles* loci containing viral segments (Segment 27 and 28 in Gf, Segment 26 in Gi) and is on a Md scaffold that contains a cluster of odv-e66 genes
Mdem_scaffold_0004	K425_1388 (conserved uncharacterized protein family 7)	Glyptapanteles flavicoxis Locus 7 (Desjardins et al. 2008): GFP_L5_0070, GFP_L5_0090, GFP_L5_0190, GFP_L5_0230, GFP_L5_0240; Glyptapanteles indiensis Locus 6, Locus 7: GIP_L2_0090, GIP_L2_0100, GIP_L7_0040, GIP_L7_0010, GIP_L7_0080	several nudivirus-like genes, closest is odv-e66 cluster	197 kb	A conserved uncharacterized gene family member in *M. demolitor* is present in several *Glyptapanteles* loci containing viral segments (Segment 27 and 28 in Gf, Segment 26 in Gi) and is on a Md scaffold that contains a cluster of odv-e66 genes
Mdem_scaffold_0040	K425_1276	Glyptapanteles indiensis Locus 2 (Desjardins et al. 2008): GIP_L8_0090 and GIP_L8_0100	cluster of pif-5 genes	190 kb	A 5' nucleotidase gene is similar to genes flanking segments 9-21 of the macrolocus in *G. indiensis.* This gene is located on a scaffold with a cluster of pif-5 genes.

Links between nudivirus-like regions and virulence genes on proviral segments

BVs

Scaffold	Segment-linked genes Md	Segment linked genes other BVs	Nudivirus-linked genes	Nearest separating distance	Note
Mdem_scaffold_0004	K425_1471	hypothetical protein Cotesia conregata bracovirus CcBV_23.4, GIP_L1_00360, and GFP_L7_0430 Ribonuclease T2	several nudivirus-like genes, closest is odv-e66 cluster	462 kb	A gene similar to BV virulence ribonuclease T2 genes is on a scaffold that also contains several nudivirus-like genes
Mdem_scaffold_0435	K425_1354, K425_1355	hypothetical protein Cotesia conregata bracovirus CcBV_2.3	ac92-like gene	2.2 kb	A gene similar to a BV virulence gene from CcBV is on a scaffold that also contains an ac92-like gene

GfVs (Glypta fumiferanae ichnovirus)

Scaffold	Segment-linked genes Md	Segment linked genes other GVs	Nudivirus-linked genes	Nearest separating distance	Note
Mdem_scaffold_0116	K425_142, K425_180	aminopeptidase-like Glypta fumiferanae ichnovirus GfV-B16-ORF1 protein, Glyptapanteles flavicoxis hypothetical protein, GFP_L5_0170, Chelonus inanitus GfV-B16-ORF1-like protein	several nudivirus-like genes, closest is pif-4	15.7 kb	A gene similar to Glypta fumiferance IV, Glyptapanteles flavicoxis BV, and Chelonus inanitus BV is present on a scaffold that also contains several nudivirus-like genes
Mdem_scaffold_0407	K425_1082	aminopeptidase-like Glypta fumiferanae ichnovirus GfV-B16-ORF1 protein, Glyptapanteles flavicoxis hypothetical protein, GFP_L5_0170, Chelonus inanitus GfV-B16-ORF1-like protein	lef-4	316 kb	A gene similar to Glypta fumiferance IV, Glyptapanteles flavicoxis BV, and Chelonus inanitus BV is present on a scaffold that also contains lef-4

Gene families present in M. demolitor that link nudivirus-like and segment containing scaffolds

Gene family name	Present on nudivirus-like scaffolds	Present on segment-containing scaffolds
conserved uncharacterized protein family 3, similar to venom protein 2 from Microctonus hyperodae	Mdem_scaffold_0275, Mdem_scaffold_0331, Mdem_scaffold_0004, Mdem_scaffold_0040, Mdem_scaffold_0407, Mdem_scaffold_0919	Mdem_scaffold_0157
cytochrome P450 domain-containing protein family	Mdem_scaffold_0004, Mdem_scaffold_0275, Mdem_scaffold_1154	Mdem_scaffold_0025
EB module-containing protein family	Mdem_scaffold_0004, Mdem_scaffold_0275, Mdem_scaffold_0407, Mdem_scaffold_0435, Mdem_scaffold_0528	Mdem_scaffold_0025
ankyrin-repeat containing protein	Mdem_scaffold_0004	Mdem_scaffold_0014, Mdem_scaffold_0025
Endoribonuclease XendoU domain-containing protein family	Mdem_scaffold_0407	Mdem_scaffold_0025
Flavin-containing monooxygenase-like family	Mdem_scaffold_0275	Mdem_scaffold_0014
protein tyrosine kinase family	Mdem_scaffold_0407	Mdem_scaffold_0014, Mdem_scaffold_0412
Haloacid dehalogenase-like hydrolase domain-containing protein family	Mdem_scaffold_0407	Mdem_scaffold_0025
Immunoglobulin domain-containing protein family	Mdem_scaffold_0275, Mdem_scaffold_1798	Mdem_scaffold_0014
P-type ATPase of unknown pump specificity (type V) family	Mdem_scaffold_0067	Mdem_scaffold_1462
Phosphatidylinositol-specific phospholipase C, X domain-containing protein family	Mdem_scaffold_0275, Mdem_scaffold_0040	Mdem_scaffold_0157
acid phosphatase-like protein family	Mdem_scaffold_0040	Mdem_scaffold_1462
kelch-like protein family	Mdem_scaffold_0275	Mdem_scaffold_0014
kinesin-like protein KIF11 family	Mdem_scaffold_0040, Mdem_scaffold_1154	Mdem_scaffold_0014
scavenger receptor class B member-like family	Mdem_scaffold_1154	Mdem_scaffold_0025
ubiquitin thioesterase trabid-like protein	Mdem_scaffold_0407	Mdem_scaffold_0025
transporter, solute:sodium symporter (SSS) family	Mdem_scaffold_0404, Mdem_scaffold_0407	Mdem_scaffold_0025
lipase domain-containing protein family	Mdem_scaffold_0240	Mdem_scaffold_0025

Gene families present in M. demolitor that link segment containing scaffolds

**Gene family name**	**Present on segment-containing scaffolds**
Histone proteins	Mdem_scaffold_0014, Mdem_scaffold_0157, Mdem_scaffold_0446, Mdem_scaffold_1462

[only includes those not already listed above]

Gene families present in M. demolitor that link nudivirus-like scaffolds

Gene family name	Present on nudivirus-like scaffolds
conserved uncharacterized protein family 1	Mdem_scaffold_0919, Mdem_scaffold_1213
conserved uncharacterized protein fam	Mdem_scaffold_0116, Mdem_scaffold_0407
conserved uncharacterized protein fam	Mdem_scaffold_0275, Mdem_scaffold_0919
alpha-tubulin-like family	Mdem_scaffold_0275, Mdem_scaffold_0528, Mdem_scaffold_0919,
annexin-like family	Mdem_scaffold_0040, Mdem_scaffold_0275
another transcription unit-like family	Mdem_scaffold_0004, Mdem_scaffold_0331
BTB/POZ domain containing protein fa	Mdem_scaffold_0275, Mdem_scaffold_0919,
disintegrin and metalloproteinase dom	Mdem_scaffold_0116, Mdem_scaffold_0407
glucose dehydrogenase acceptor-like f	Mdem_scaffold_0004, Mdem_scaffold_0407, Mdem_scaffold_1154
lipase 3-like family	Mdem_scaffold_0004, Mdem_scaffold_0407
MFS sugar transporter domain-contain	Mdem_scaffold_0004, Mdem_scaffold_0938
neprilysin-like family	Mdem_scaffold_0067, Mdem_scaffold_0938, Mdem_scaffold_1154
clavesin-2-like family	Mdem_scaffold_0004, Mdem_scaffold_0407

[only includes those not already listed above]
