# Supplementary material for: Widespread Genome Reorganization of an Obligate Virus Mutualist
Source: PLoS Genet. 2014 Sep 18;10(9):e1004660. doi: 10.1371/journal.pgen.1004660 (PMC4169385; doi:10.1371/journal.pgen.1004660)
Supplement: Table S3 — PCR primers used in this study. (PDF) [file pgen.1004660.s007.pdf]

Segment	Primer name	Sequence 5’-3’	Expected product (bp)
Segment P	P-F	AGGGATCCAATTGTTTATAATTTCC	705
	P-R	CGTTATGTATGAAATTAGCTTCTCAA	
Segment K1	K1_F	TTGATTATTTTTATTGGTCTCAGAAA	718
	K1_R	GTAATGATTGTCAACTCGGAAAAA	
Segment K	K-F	TGCACTAGGTATGATGGTCAGC	666
	K-R	ATTCAACCGAGGCTTGGACTA	
Segment Q	Q-F	TGCCTAGTAGGGGTCGAGAA	418
	Q-R	CCAATTCAATGGACGTTTGG	
Segment D	D-F	CCAGAGGCTACCAGGAATGA	418
	D-R	GTGCGGACCTTCAGCAACTA	
Segment S	S-F	TGTTATCTAAGAGGCATGCTATGG	404
	S-R	TGAACATTTACGCTGACAAGC	
Segment T	T-F	AACGAATGACGATCGGTCTG	400
	T-R	CTAGCACTGATTGCCAGGTC	
Segment U	U-F	GATTTTGAACCGTACAGGGATT	403
	U-R	GAGCCCATCCCAAAAAGATA	
Segment V	V-F	CATGGCGACGTGATGTCTAC	475
	V-R	TATCATCGGTCGGTTTTTCG	
Segment W	W-F	TACCTCGACTAGCGGTGCTT	437
	W-R	GCGAACGGTCACTACTGAAA	
Segment X	X-F	GAAAATTTCGATTTGGAGACCA	428
	X-R	GCCAGATAGTTTGTCGCACTT	
Segment R	R-F	TCATCTGTTTTGCGGACATT	439
	R-R	TGGATATGATCATGATGCTTAATTT	
Segment A	A-F	ATTTGCGCCGGTATGAAGTA	577
	A-R	ACATAGAGGCGCCAAACAAG	
Segment B	B-F	TGCCGGAAACTTTTCTAACG	631
	B-R	GGTGCTGAATCCGTTCATTT	
